# Supplementary material for: A software tool and strategy for peptidoglycomics, the high-resolution analysis of bacterial peptidoglycans via LC-MS/MS
Source: Commun Chem. 2025 Mar 26;8:91. doi: 10.1038/s42004-025-01490-6 (PMC11937551; doi:10.1038/s42004-025-01490-6)
Supplement: Supplementary file 2 — Supplementary Information [file 42004_2025_1490_MOESM2_ESM.pdf]

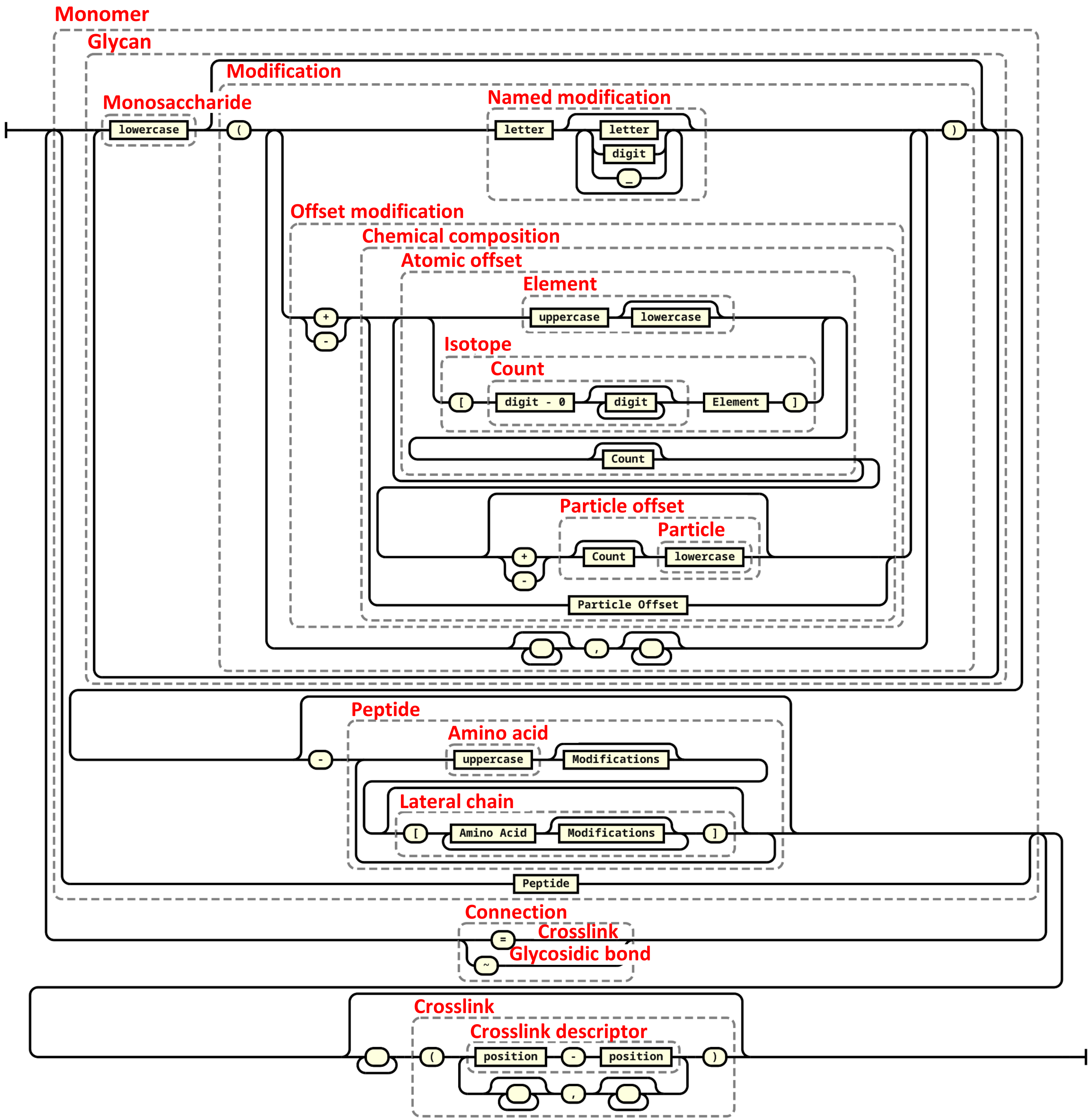

Figure S1: A railroad syntax diagram for PGLang. Any path connecting the starting point at the top left to the ending point in the bottom right — including any loops or skipped sections — describes a syntactically legal PGLang structure. Note, however, that not every syntactically valid structure is semantically valid (i.e. it's possible to describe structures in PGLang that are chemically impossible, but those errors will be caught during a subsequent validation step). Lowercase letters are all ASCII characters 'a'–'z', and uppercase letters are all characters 'A'–'Z'; the "letters" character set is a combination of the two. Digits are ASCII '0'–'9', and positions are '1'–'5'.

Figure S2. Monomers identified by MS/MS in TY samples using Byonic™ software.

Monomer	Max ions expected	*b* ions	*y* ions
**Validated monomers**			
gm-AE	1	1	1
gm-AEJ	2	2	2
gm-AEJA	3	3	3
gm-AEJD		2	3
gm-AEJF		3	3
gm-AEJG		3	3
gm-AEJH		3	3
gm-AEJI		3	3
gm-AEJK		3	3
gm-AEJM		3	3
gm-AEJN		3	3
gm-AEJQ [a]		2	3
gm-AEJS		3	3
gm-AEJT		3	3
gm-AEJV		3	3
gm-AEJW		3	2
gm-AEJY		3	3
gm-AEJAA		4	4
gm-AEJAD		4	3
gm-AEJAE		4	3
gm-AEJAF		4	4
gm-AEJAG [a]		4	3
gm-AEJAH	4	3	4
gm-AEJAI		4	4
gm-AEJAK		3	4
gm-AEJAM		4	4
gm-AEJAQ		4	4
gm-AEJAR		3	4
gm-AEJAW		4	4
**Non validated monomers**			
gm-AEJR	3	**1**	3
gm-AEJQ [a]		**2**	3
gm-AEJDK		**2**	3
gm-AEJMS		**2**	3
gm-AEJGA [b]		3	4
gm-AEJAN [b]	4	3	3
gm-AEJAY [b]		4	4
gm-AEJQE [c]		3	3
gm-AEJEQ [c]		3	4
gm-AEJWW [c]		4	3

[a] Exact mass coincidence
gm-AEJQ = gm-AEJQ 998.429165 Da
[b] only one MS/SM spectrum
[c] mass coincidences (EQ=QE=ADA; WW=JEA)

VALIDATED SPECTRA

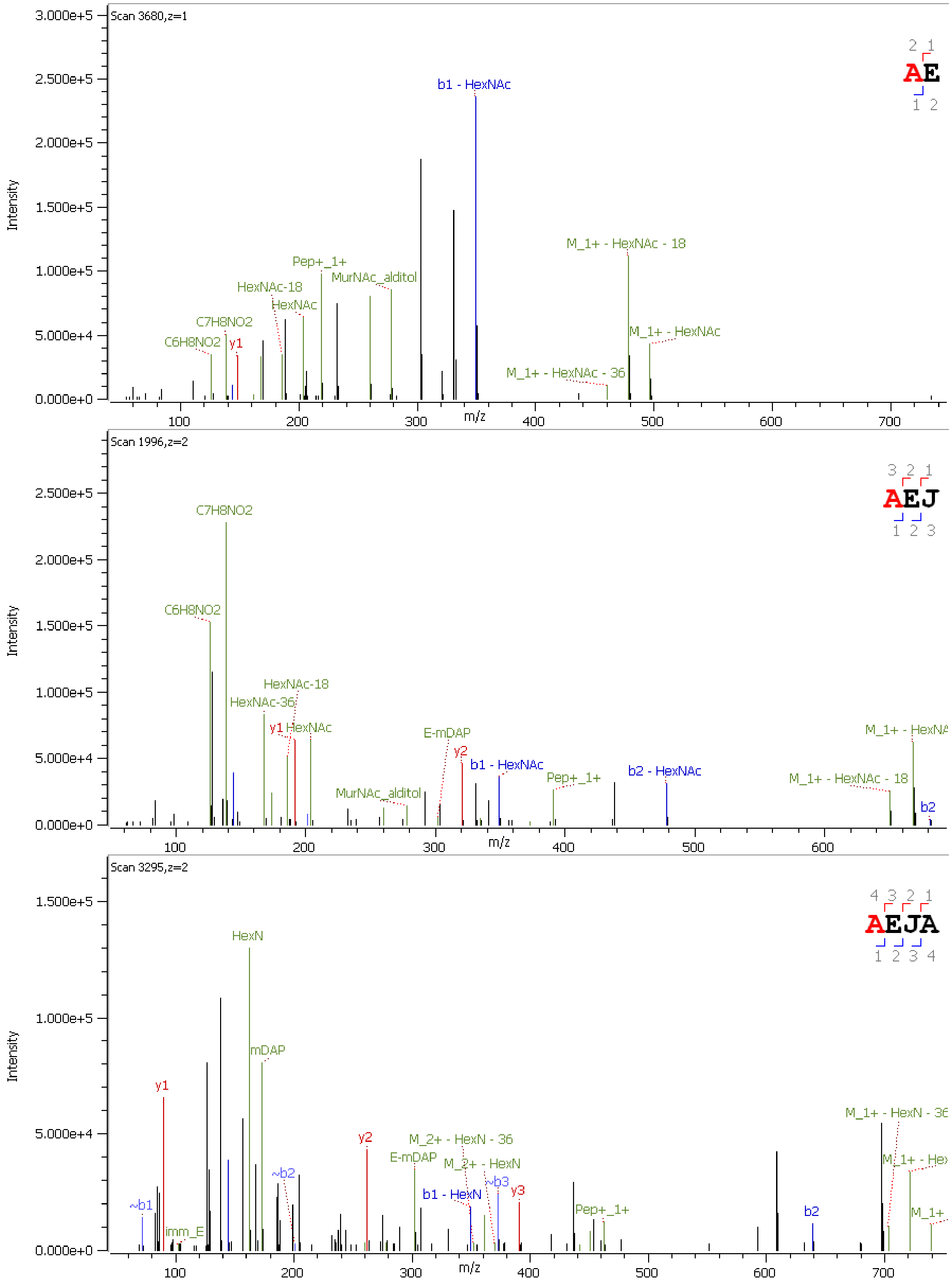

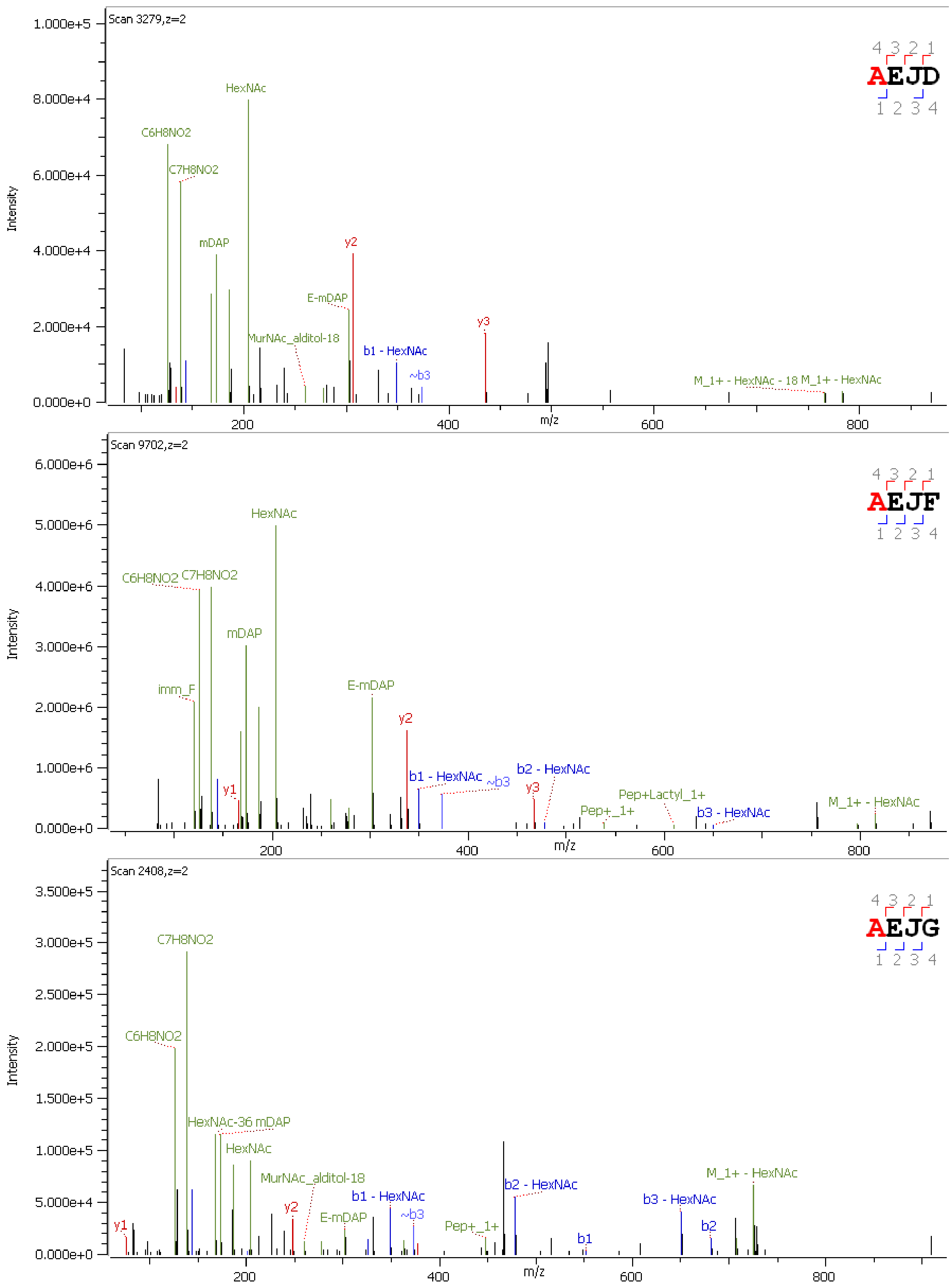

Scan 3279,z=2
AEJD
4 3 2 1
1 2 3 4
Intensity
HexNAc
C6H8NO2
C7H8NO2
mDAP
y2
E-mDAP
y3
MurNAc_alditol-18
b1 - HexNAc
~b3
M_1+ - HexNAc - 18 M_1+ - HexNAc
m/z
Scan 9702,z=2
AEJF
HexNAc
C6H8NO2 C7H8NO2
mDAP
imm_F
E-mDAP
y2
b1 - HexNAc
~b3
b2 - HexNAc
y1
y3
Pep+Lactyl_1+
Pep+_1+
b3 - HexNAc
M_1+ - HexNAc
Scan 2408,z=2
AEJG
C7H8NO2
C6H8NO2
HexNAc-36 mDAP
HexNAc
MurNAc_alditol-18
b1 - HexNAc
~b3
E-mDAP
y1
y2
Pep+_1+
b2 - HexNAc
b1
b3 - HexNAc
b2
M_1+ - HexNAc

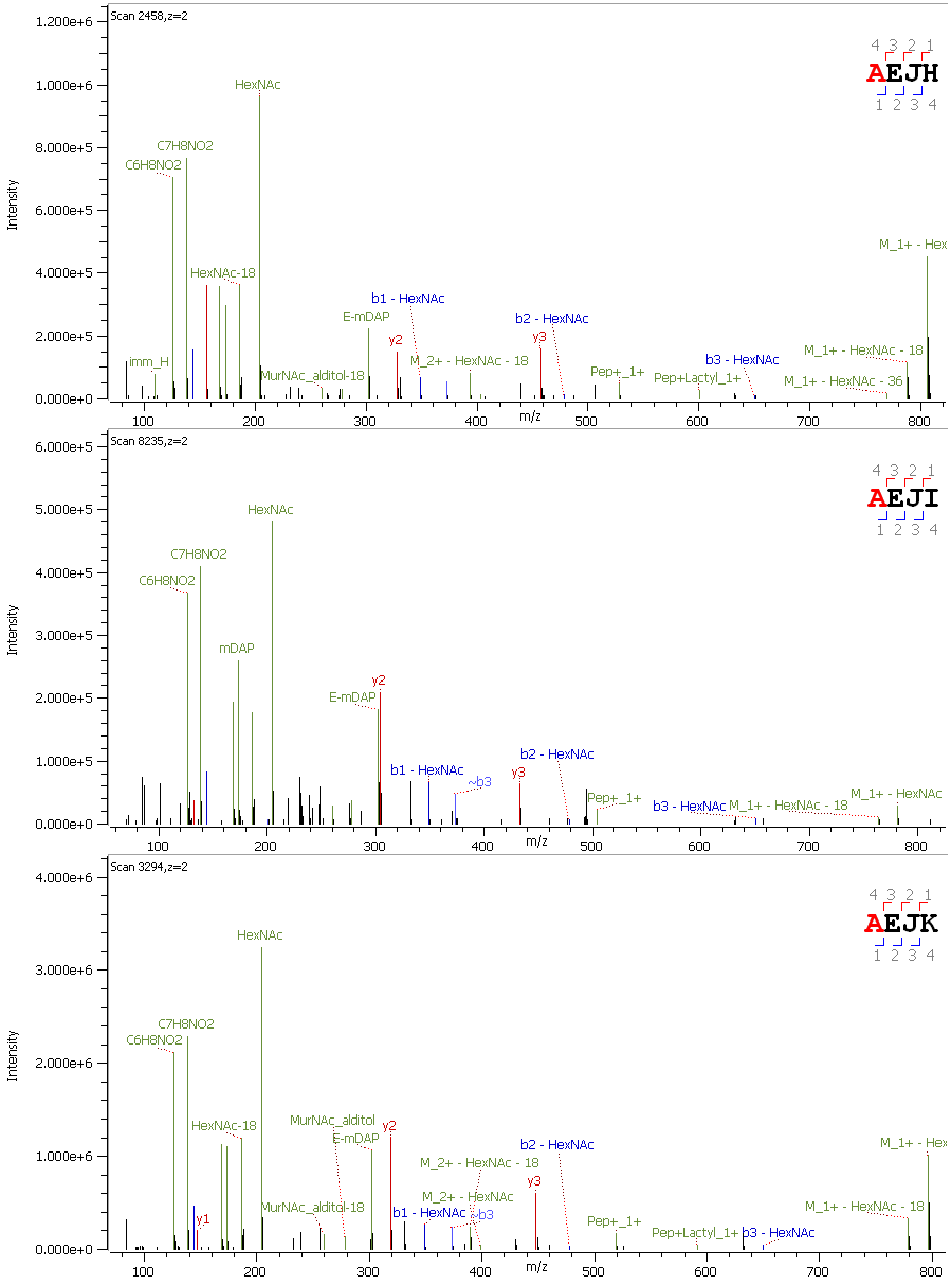
Scan 2458,z=2
AEJH
Intensity
m/z
HexNAc
C7H8NO2
C6H8NO2
HexNAc-18
imm_H
MurNAc_alditol-18
E-mDAP
b1 - HexNAc
y2
M_2+ - HexNAc - 18
b2 - HexNAc
y3
Pep+_1+
Pep+Lactyl_1+
b3 - HexNAc
M_1+ - HexNAc - 18
M_1+ - HexNAc - 36
M_1+ - Hex
Scan 8235,z=2
AEJI
HexNAc
C7H8NO2
C6H8NO2
mDAP
y2
E-mDAP
b1 - HexNAc
~b3
y3
b2 - HexNAc
Pep+_1+
b3 - HexNAc
M_1+ - HexNAc - 18
M_1+ - HexNAc
Scan 3294,z=2
AEJK
HexNAc
C7H8NO2
C6H8NO2
HexNAc-18
MurNAc_alditol
y2
E-mDAP
y1
MurNAc_alditol-18
b1 - HexNAc
~b3
M_2+ - HexNAc - 18
M_2+ - HexNAc
b2 - HexNAc
y3
Pep+_1+
Pep+Lactyl_1+
b3 - HexNAc
M_1+ - HexNAc - 18
M_1+ - Hex

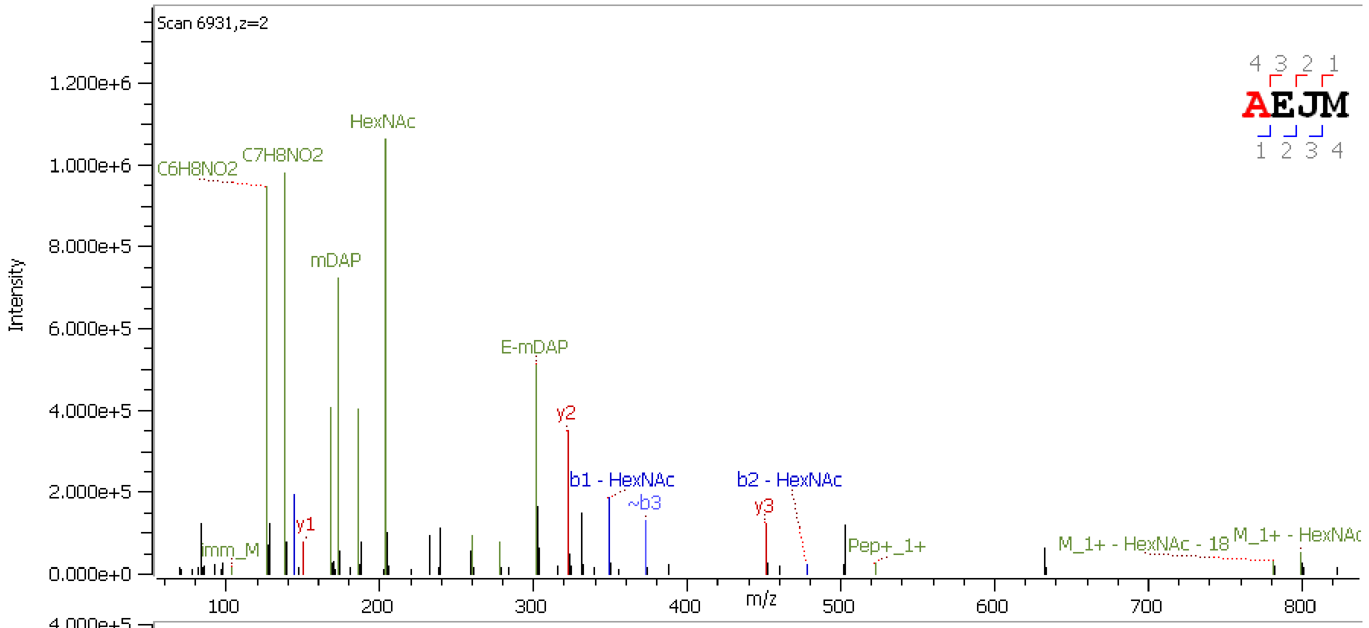
Scan 6931,z=2
AEJM
4 3 2 1
1 2 3 4
Intensity
m/z
HexNAc
C6H8NO2
C7H8NO2
mDAP
E-mDAP
y2
y1
y3
imm_M
b1 - HexNAc
~b3
b2 - HexNAc
Pep+_1+
M_1+ - HexNAc - 18
M_1+ - HexNAc
0.000e+0
2.000e+5
4.000e+5
6.000e+5
8.000e+5
1.000e+6
1.200e+6
100
200
300
400
500
600
700
800

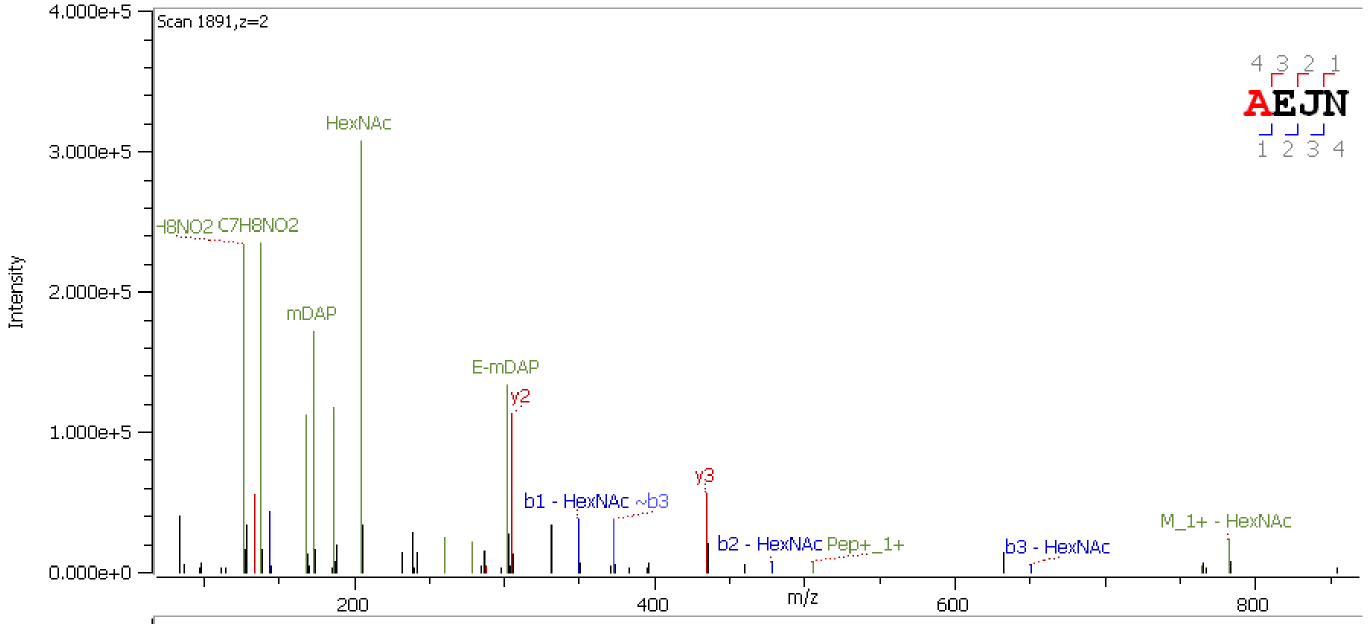
Scan 1891,z=2
AEJN
4 3 2 1
1 2 3 4
Intensity
m/z
HexNAc
C7H8NO2
mDAP
E-mDAP
y2
y3
b1 - HexNAc
~b3
b2 - HexNAc
Pep+_1+
b3 - HexNAc
M_1+ - HexNAc
0.000e+0
1.000e+5
2.000e+5
3.000e+5
4.000e+5
200
400
600
800

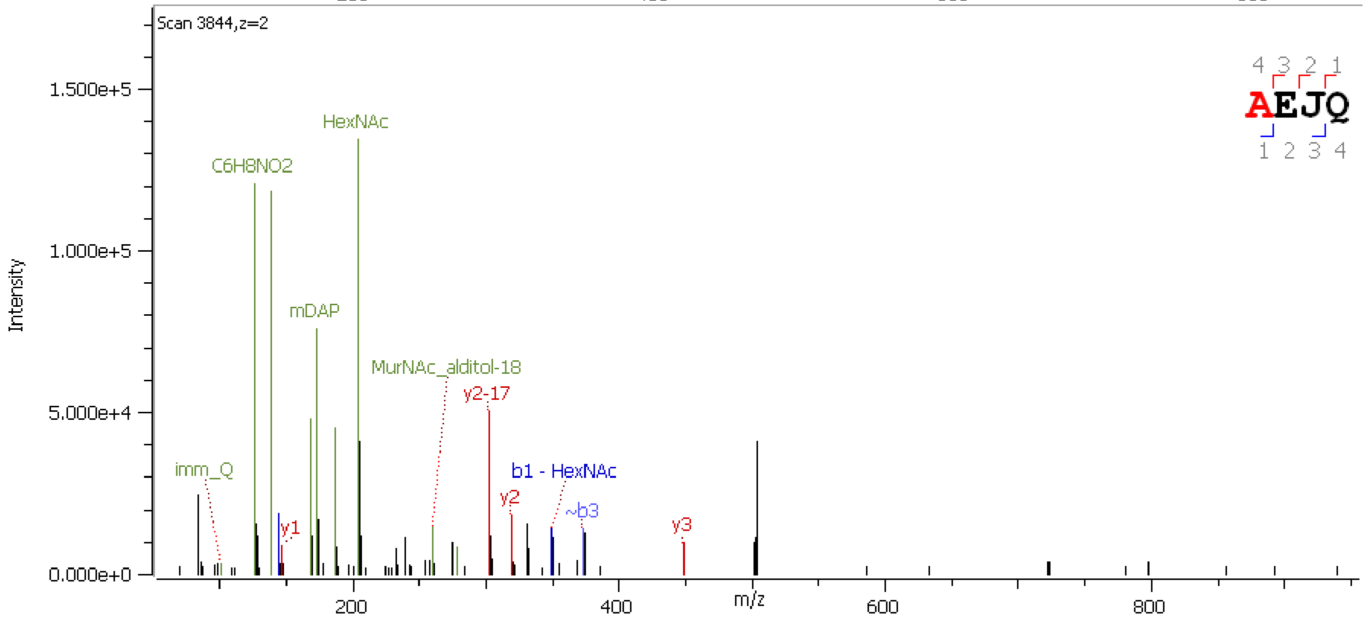
Scan 3844,z=2
AEJQ
4 3 2 1
1 2 3 4
Intensity
m/z
HexNAc
C6H8NO2
mDAP
MurNAc_alditol-18
y2-17
imm_Q
b1 - HexNAc
y2
~b3
y1
y3
0.000e+0
5.000e+4
1.000e+5
1.500e+5
200
400
600
800

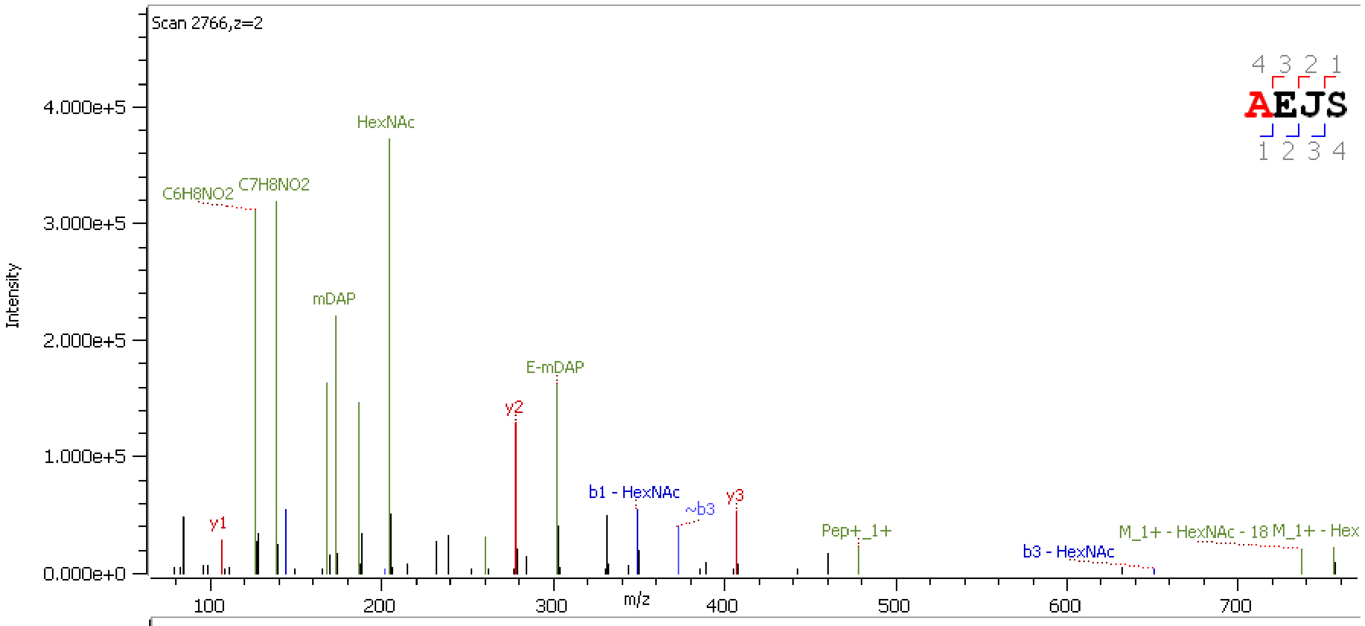
Scan 2766,z=2
Intensity
4.000e+5
3.000e+5
2.000e+5
1.000e+5
0.000e+0
m/z
100
200
300
400
500
600
700
HexNAc
C6H8NO2
C7H8NO2
mDAP
E-mDAP
y2
y1
b1 - HexNAc
~b3
y3
Pep+_1+
b3 - HexNAc
M_1+ - HexNAc - 18
M_1+ - Hex
4 3 2 1
AEJS
1 2 3 4

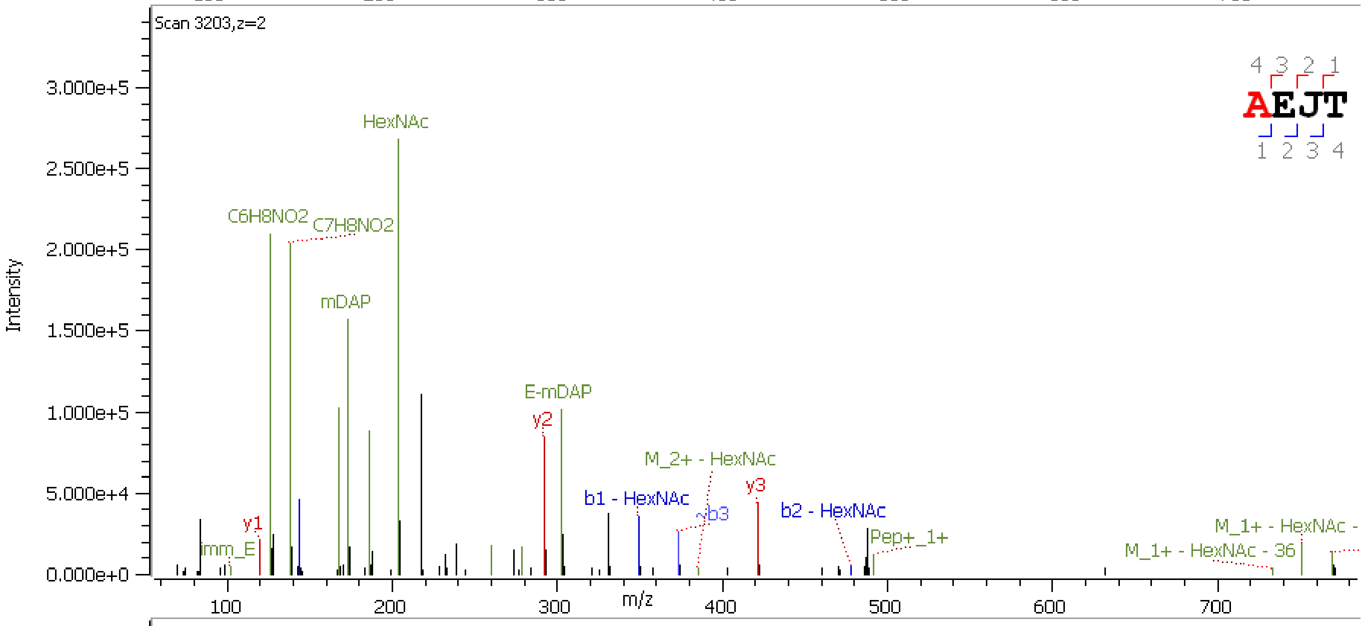
Scan 3203,z=2
Intensity
3.000e+5
2.500e+5
2.000e+5
1.500e+5
1.000e+5
5.000e+4
0.000e+0
m/z
100
200
300
400
500
600
700
HexNAc
C6H8NO2
C7H8NO2
mDAP
E-mDAP
y2
M_2+ - HexNAc
y3
b1 - HexNAc
~b3
b2 - HexNAc
Pep+_1+
y1
imm_E
M_1+ - HexNAc -
M_1+ - HexNAc - 36
4 3 2 1
AEJT
1 2 3 4

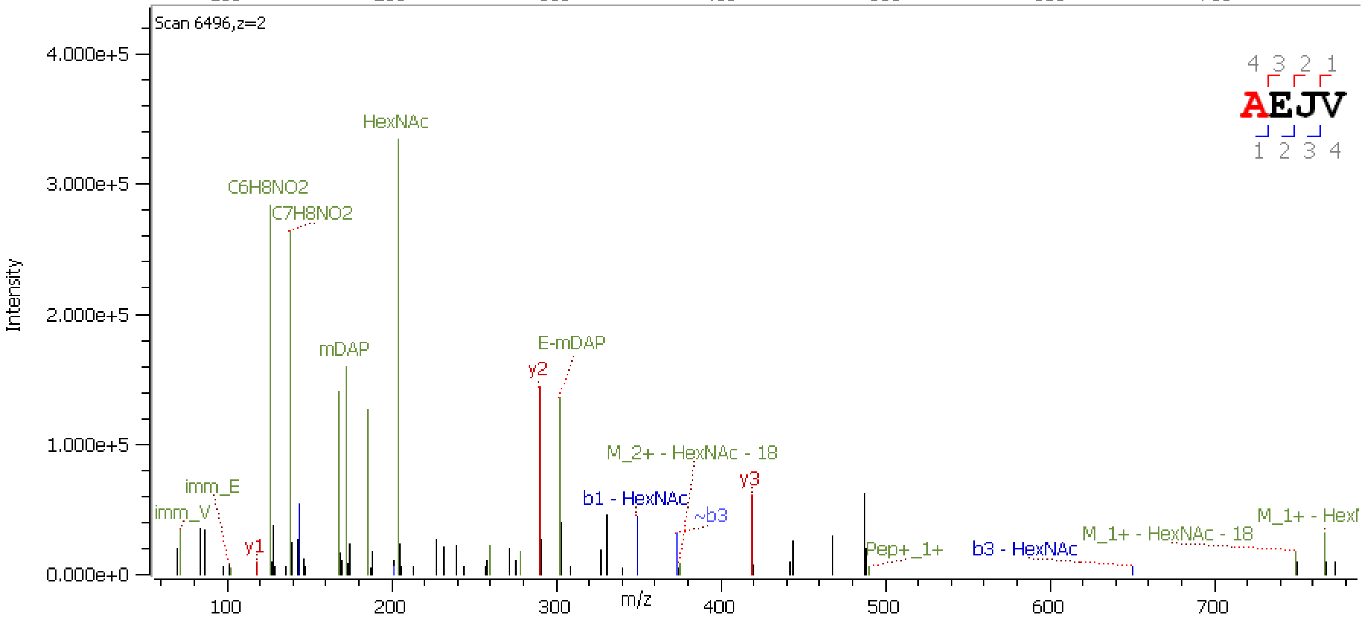
Scan 6496,z=2
Intensity
4.000e+5
3.000e+5
2.000e+5
1.000e+5
0.000e+0
m/z
100
200
300
400
500
600
700
HexNAc
C6H8NO2
C7H8NO2
mDAP
E-mDAP
y2
M_2+ - HexNAc - 18
y3
b1 - HexNAc
~b3
imm_E
imm_V
y1
Pep+_1+
b3 - HexNAc
M_1+ - HexNAc - 18
4 3 2 1
AEJV
1 2 3 4

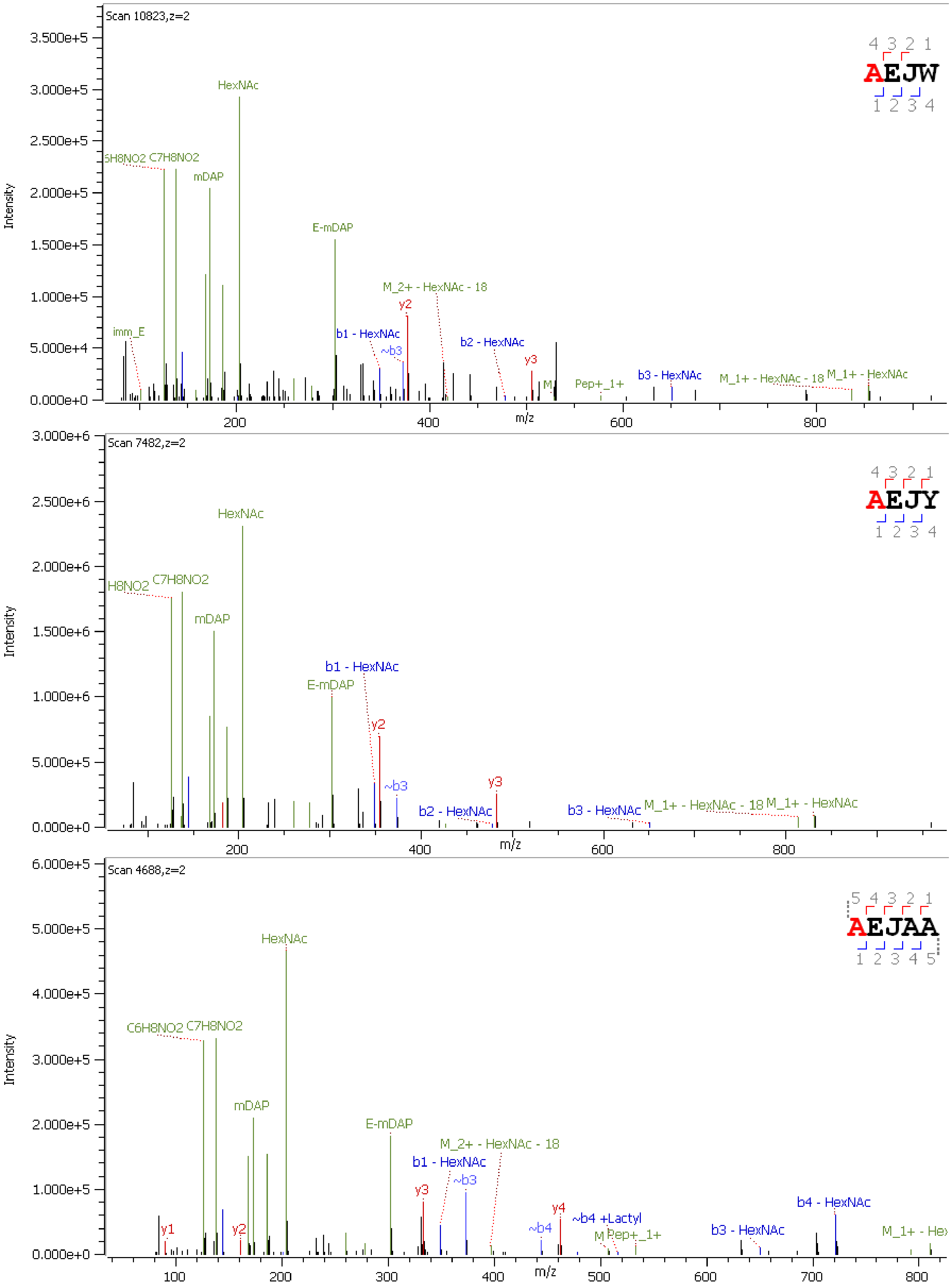

Scan 10823,z=2
Intensity
m/z
HexNAc
C7H8NO2
mDAP
E-mDAP
M_2+ - HexNAc - 18
y2
b1 - HexNAc
~b3
b2 - HexNAc
y3
imm_E
Pep+_1+
b3 - HexNAc
M_1+ - HexNAc - 18
M_1+ - HexNAc
AEJW
Scan 7482,z=2
Intensity
m/z
HexNAc
H8NO2
C7H8NO2
mDAP
b1 - HexNAc
E-mDAP
y2
~b3
y3
b2 - HexNAc
b3 - HexNAc
M_1+ - HexNAc - 18
M_1+ - HexNAc
AEJY
Scan 4688,z=2
Intensity
m/z
HexNAc
C6H8NO2
C7H8NO2
mDAP
E-mDAP
M_2+ - HexNAc - 18
b1 - HexNAc
~b3
y3
y1
y2
y4
~b4
~b4 +Lactyl
Pep+_1+
b3 - HexNAc
b4 - HexNAc
AEJAA

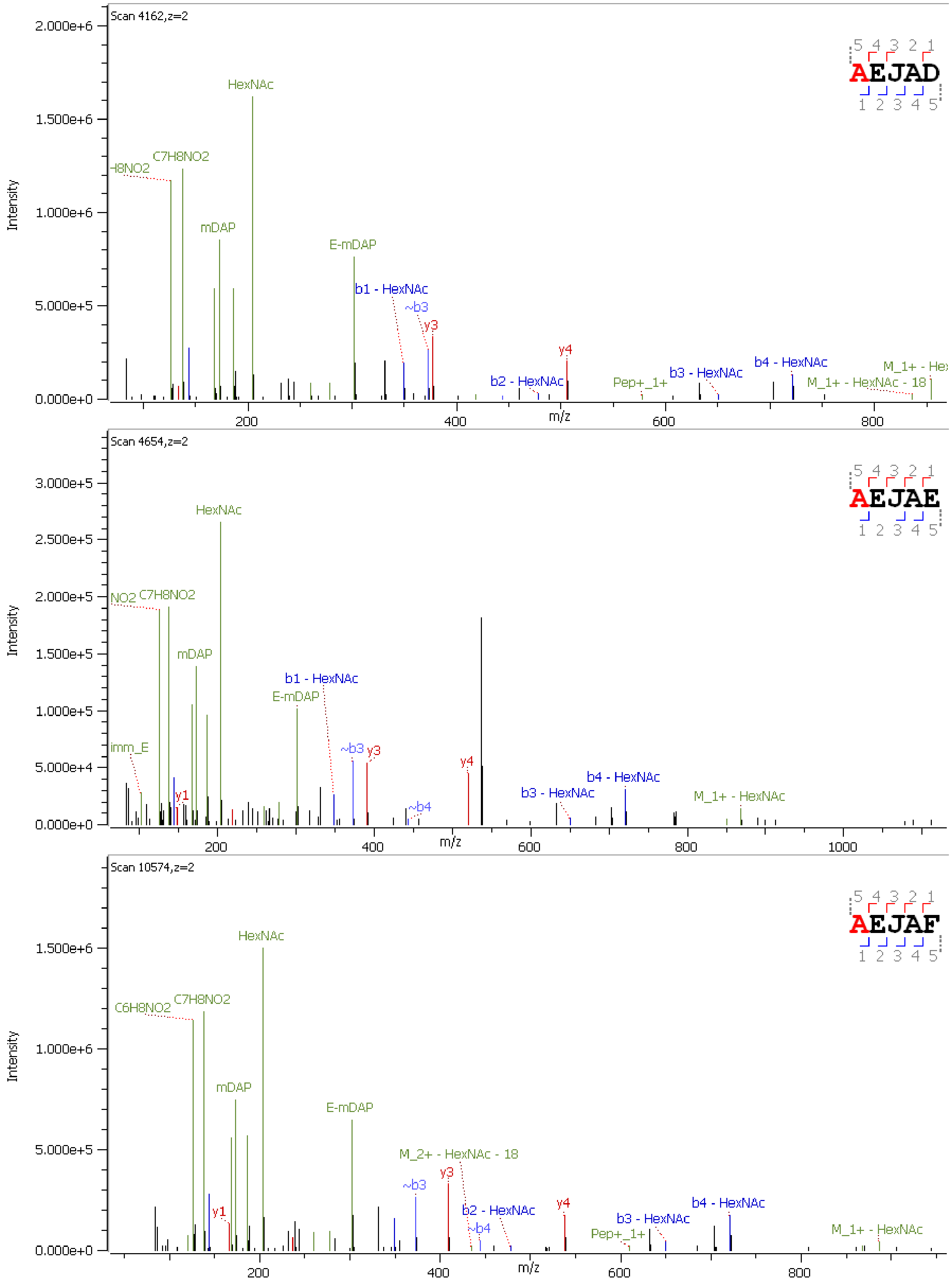

Scan 4162,z=2
AEJAD
HexNAc
C7H8NO2
mDAP
E-mDAP
b1 - HexNAc
~b3
y3
y4
b2 - HexNAc
Pep+_1+
b3 - HexNAc
b4 - HexNAc
M_1+ - HexNAc - 18
Intensity
m/z
Scan 4654,z=2
AEJAE
HexNAc
C7H8NO2
mDAP
b1 - HexNAc
E-mDAP
imm_E
~b3
y3
y1
y4
~b4
b3 - HexNAc
b4 - HexNAc
M_1+ - HexNAc
Intensity
m/z
Scan 10574,z=2
AEJAF
HexNAc
C6H8NO2
C7H8NO2
mDAP
E-mDAP
M_2+ - HexNAc - 18
y3
~b3
y1
y4
b2 - HexNAc
~b4
b3 - HexNAc
b4 - HexNAc
Pep+_1+
M_1+ - HexNAc
Intensity
m/z

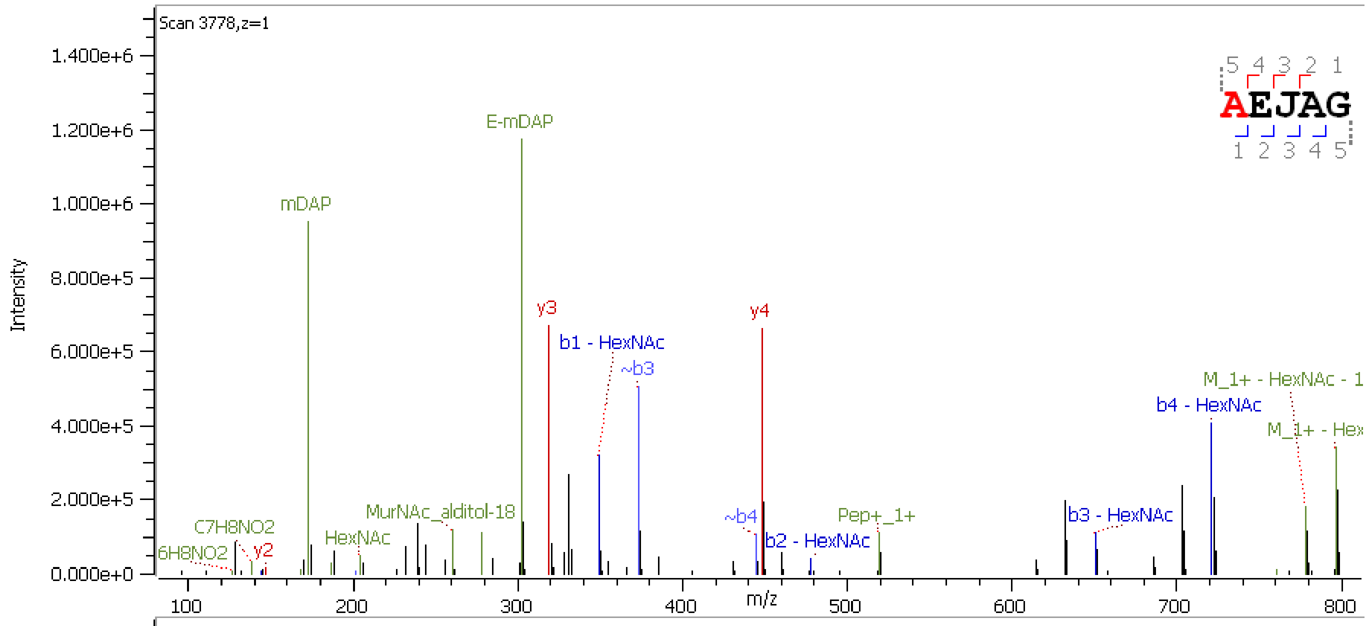
Scan 3778,z=1
AEJAG
Intensity
m/z
E-mDAP
mDAP
y3
b1 - HexNAc
~b3
y4
M_1+ - HexNAc - 1
b4 - HexNAc
M_1+ - Hex
C7H8NO2
6H8NO2
y2
HexNAc
MurNAc_alditol-18
~b4
b2 - HexNAc
Pep+_1+
b3 - HexNAc
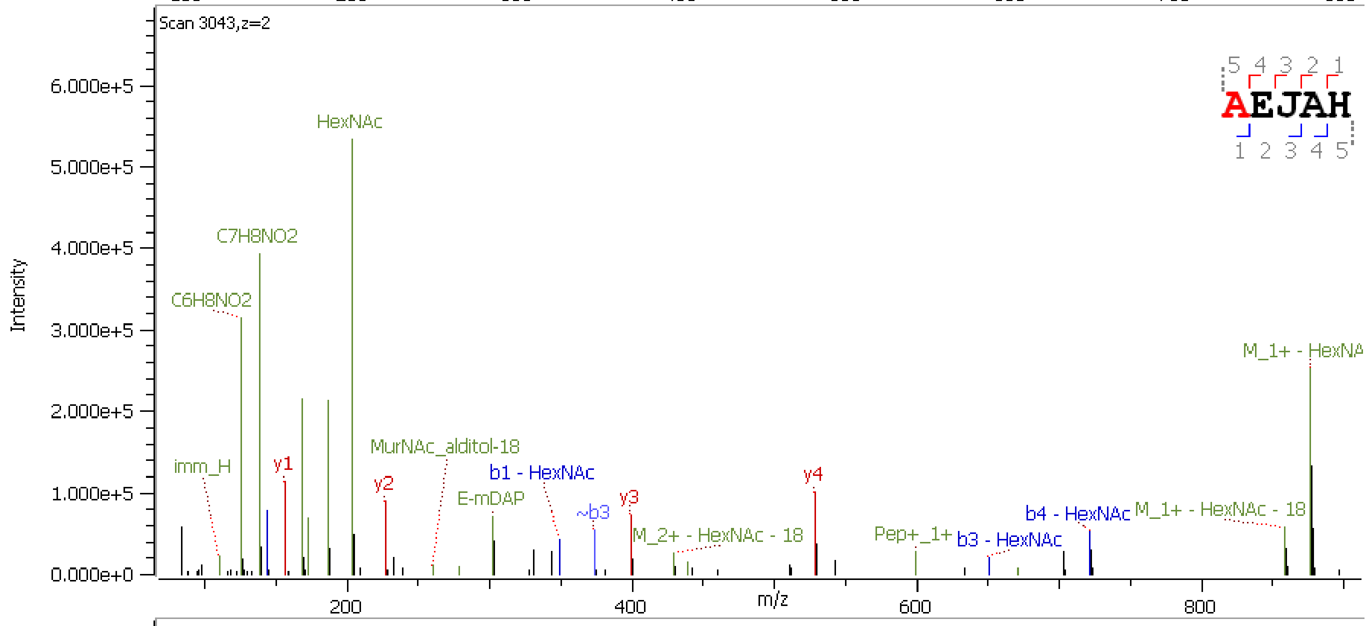
Scan 3043,z=2
AEJAH
Intensity
m/z
HexNAc
C7H8NO2
C6H8NO2
M_1+ - HexNA
imm_H
y1
y2
MurNAc_alditol-18
b1 - HexNAc
E-mDAP
~b3
y3
y4
M_2+ - HexNAc - 18
Pep+_1+
b3 - HexNAc
b4 - HexNAc
M_1+ - HexNAc - 18
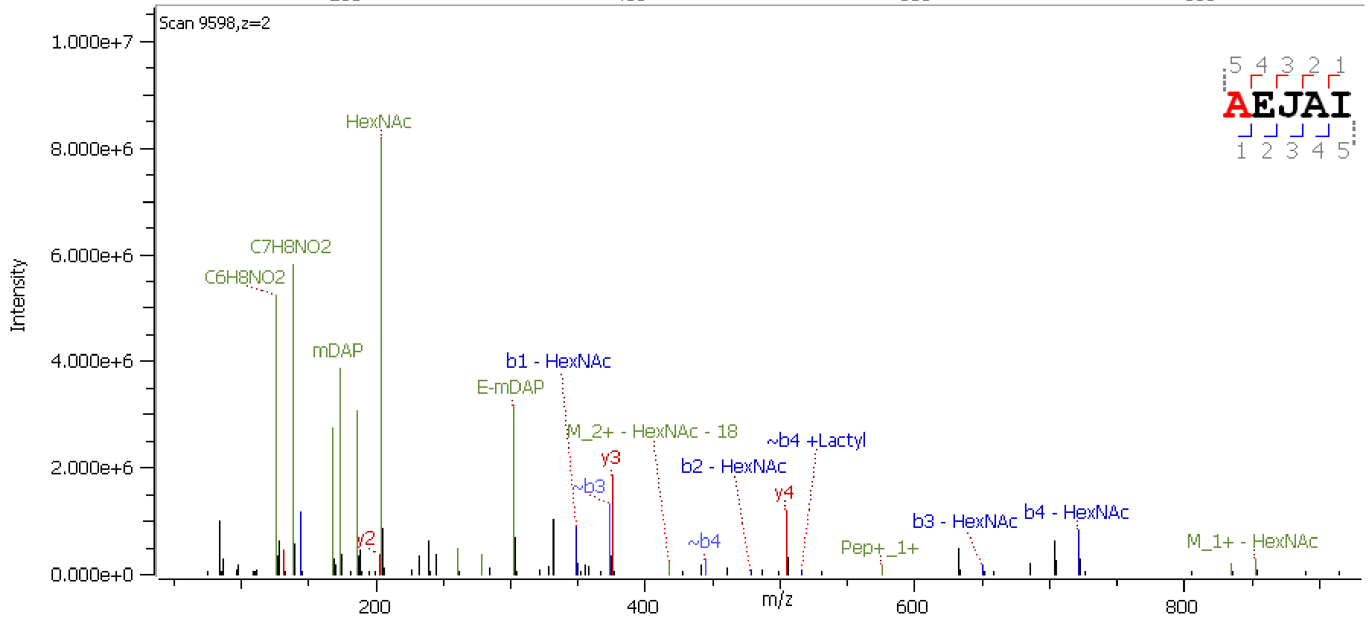
Scan 9598,z=2
AEJAI
Intensity
m/z
HexNAc
C7H8NO2
C6H8NO2
mDAP
b1 - HexNAc
E-mDAP
M_2+ - HexNAc - 18
~b4 +Lactyl
y3
b2 - HexNAc
y4
~b3
y2
~b4
Pep+_1+
b3 - HexNAc
b4 - HexNAc
M_1+ - HexNAc

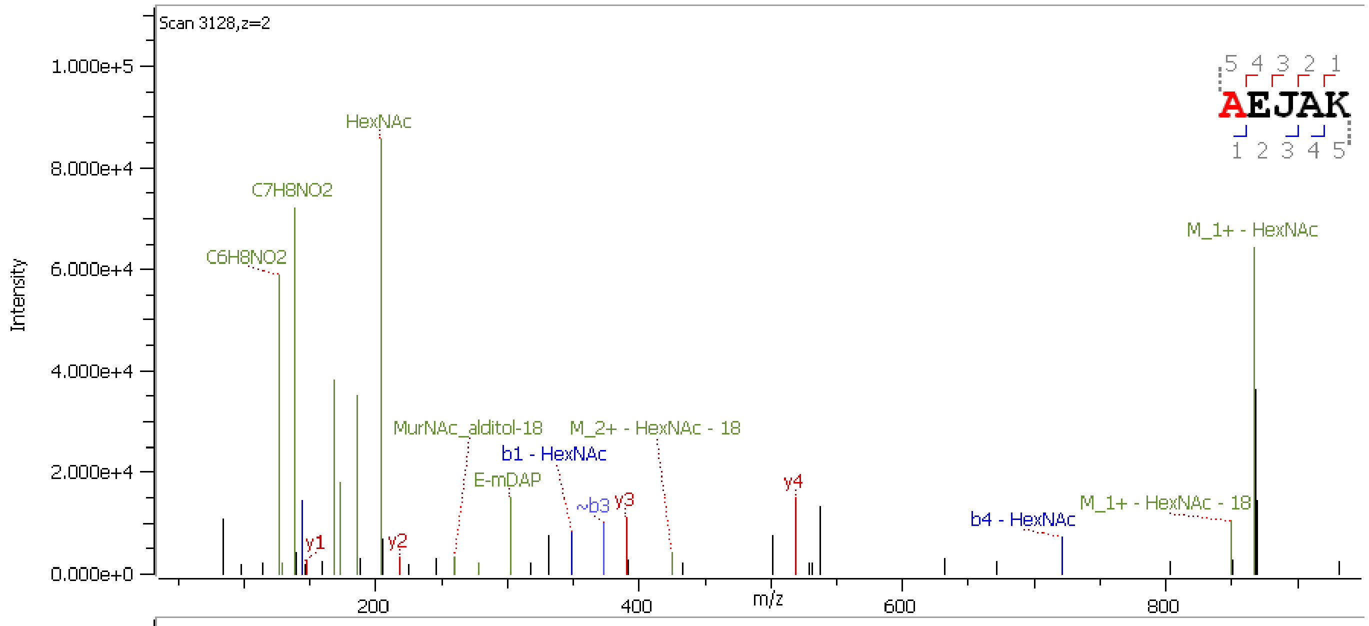
Scan 3128,z=2
Intensity
1.000e+5
8.000e+4
6.000e+4
4.000e+4
2.000e+4
0.000e+0
m/z
200
400
600
800
AEJAK
5 4 3 2 1
1 2 3 4 5
HexNAc
C7H8NO2
C6H8NO2
M_1+ - HexNAc
MurNAc_alditol-18
M_2+ - HexNAc - 18
b1 - HexNAc
E-mDAP
~b3
y3
y4
M_1+ - HexNAc - 18
b4 - HexNAc
y1
y2

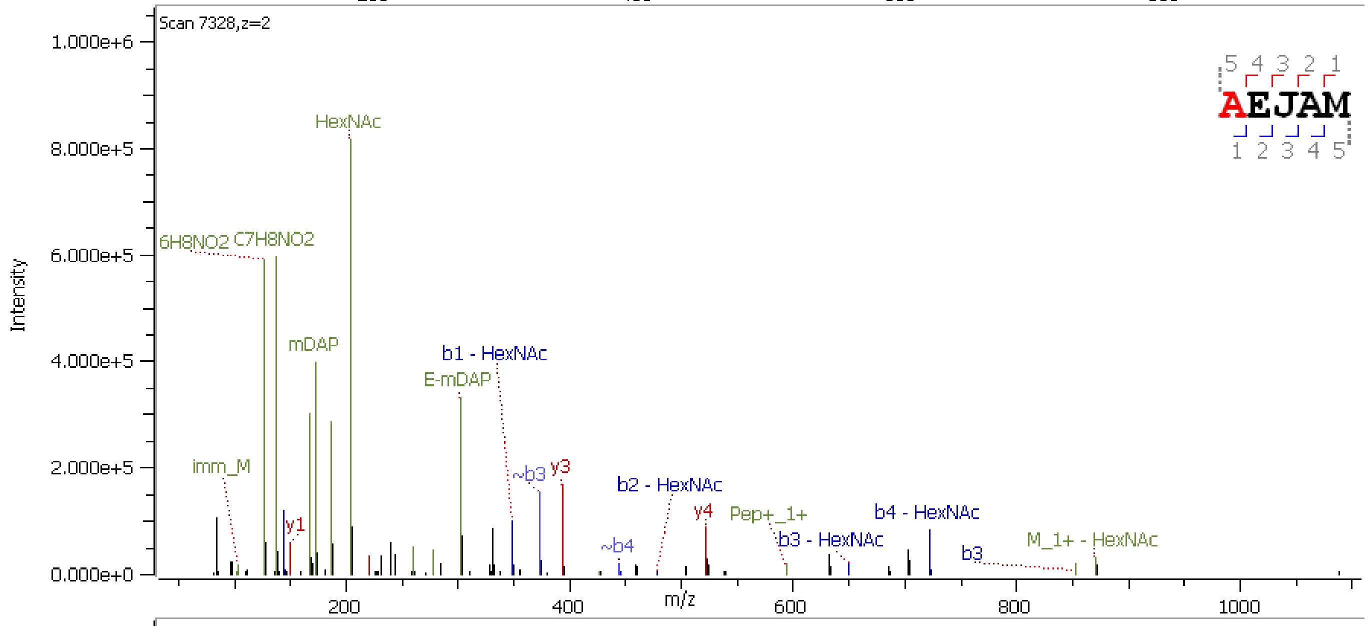
Scan 7328,z=2
Intensity
1.000e+6
8.000e+5
6.000e+5
4.000e+5
2.000e+5
0.000e+0
m/z
200
400
600
800
1000
AEJAM
5 4 3 2 1
1 2 3 4 5
HexNAc
6H8NO2
C7H8NO2
mDAP
b1 - HexNAc
E-mDAP
imm_M
~b3
y3
b2 - HexNAc
y4
Pep+_1+
b4 - HexNAc
y1
~b4
b3 - HexNAc
b3
M_1+ - HexNAc

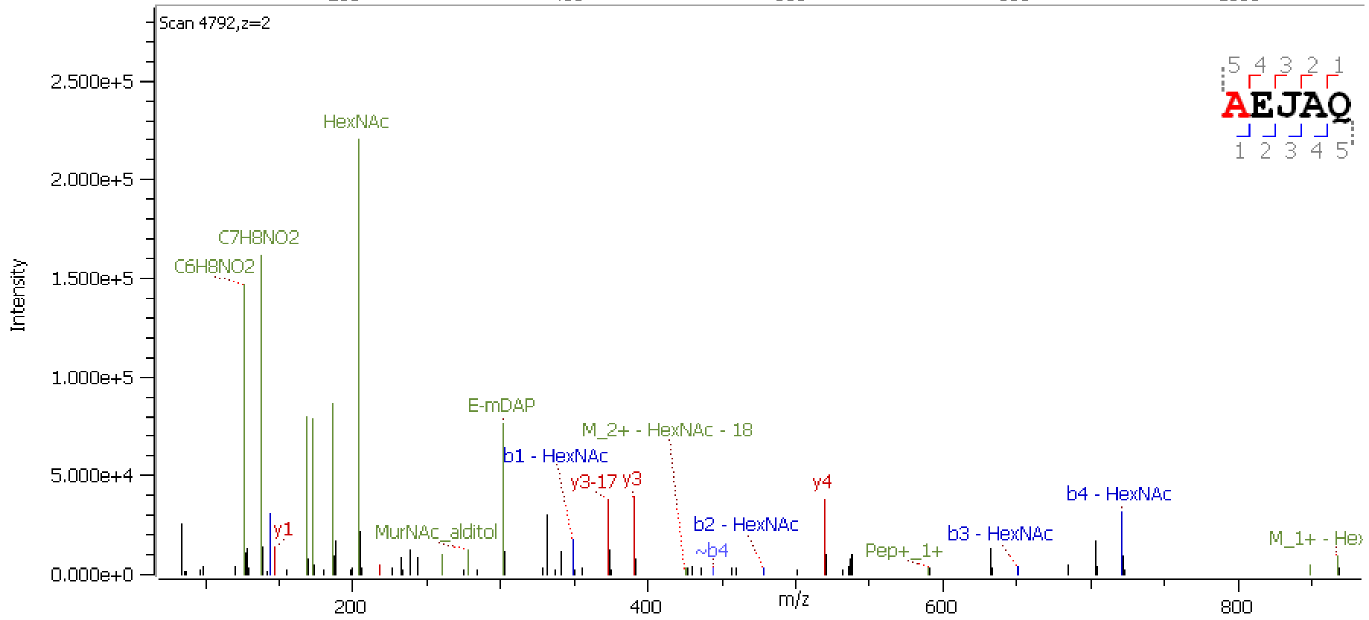
Scan 4792,z=2
Intensity
2.500e+5
2.000e+5
1.500e+5
1.000e+5
5.000e+4
0.000e+0
m/z
200
400
600
800
AEJAQ
5 4 3 2 1
1 2 3 4 5
HexNAc
C7H8NO2
C6H8NO2
E-mDAP
M_2+ - HexNAc - 18
b1 - HexNAc
y3-17
y3
y4
b4 - HexNAc
y1
MurNAc_alditol
b2 - HexNAc
~b4
b3 - HexNAc
Pep+_1+

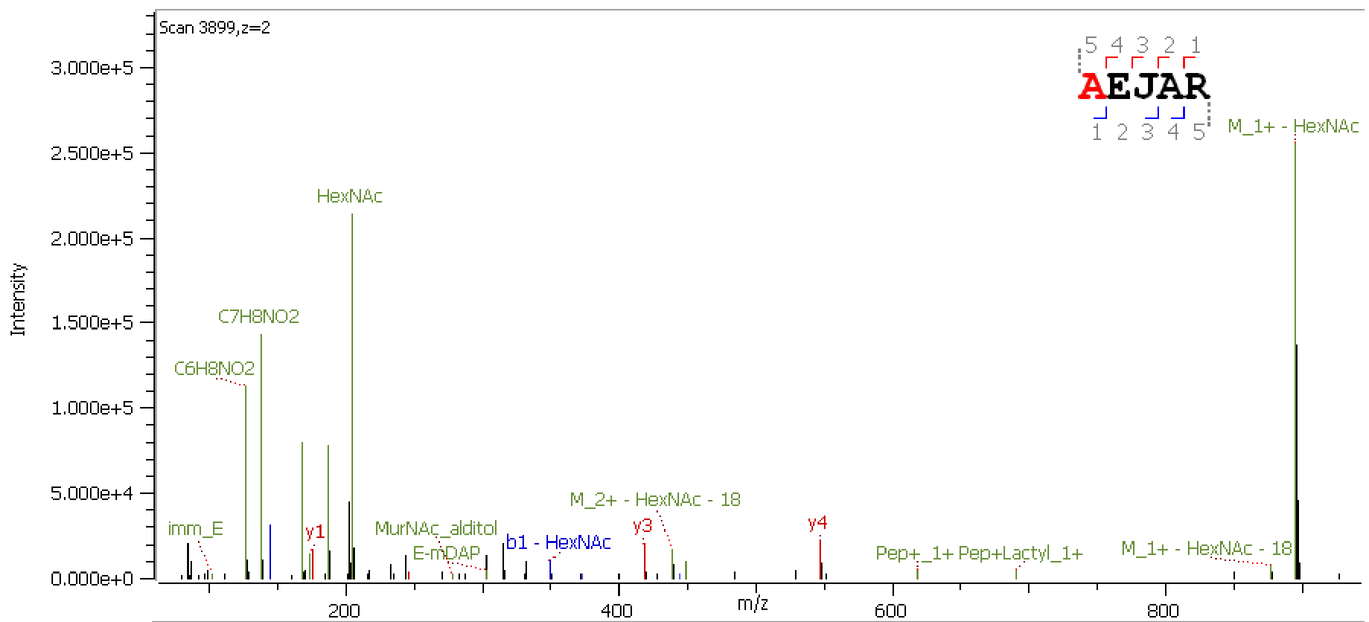
Scan 3899,z=2
AEJAR
Intensity
m/z
HexNAc
C7H8NO2
C6H8NO2
imm_E
y1
MurNAc_alditol
E-mDAP
b1 - HexNAc
M_2+ - HexNAc - 18
y3
y4
Pep+_1+ Pep+Lactyl_1+
M_1+ - HexNAc - 18
M_1+ - HexNAc

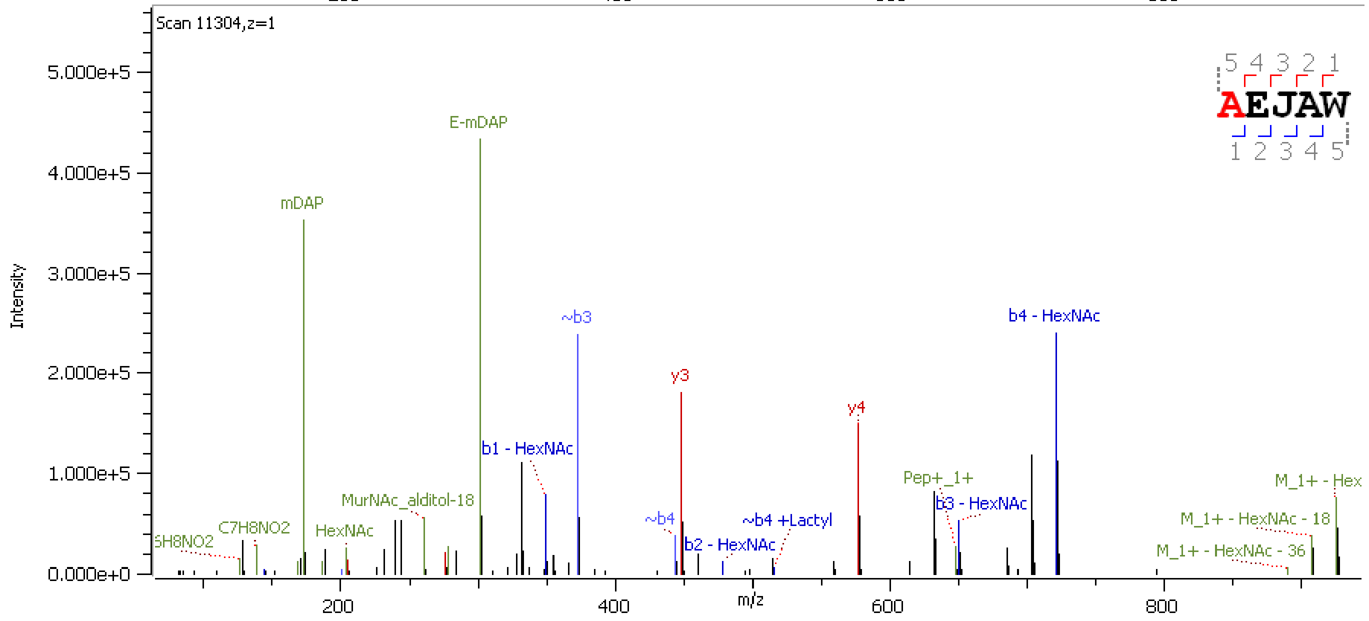
Scan 11304,z=1
AEJAW
Intensity
m/z
E-mDAP
mDAP
~b3
b4 - HexNAc
y3
y4
b1 - HexNAc
MurNAc_alditol-18
C7H8NO2
HexNAc
~b4
b2 - HexNAc
~b4 +Lactyl
Pep+_1+
b3 - HexNAc
M_1+ - Hex
M_1+ - HexNAc - 18
M_1+ - HexNAc - 36

NON Validated MS/MS spectra:

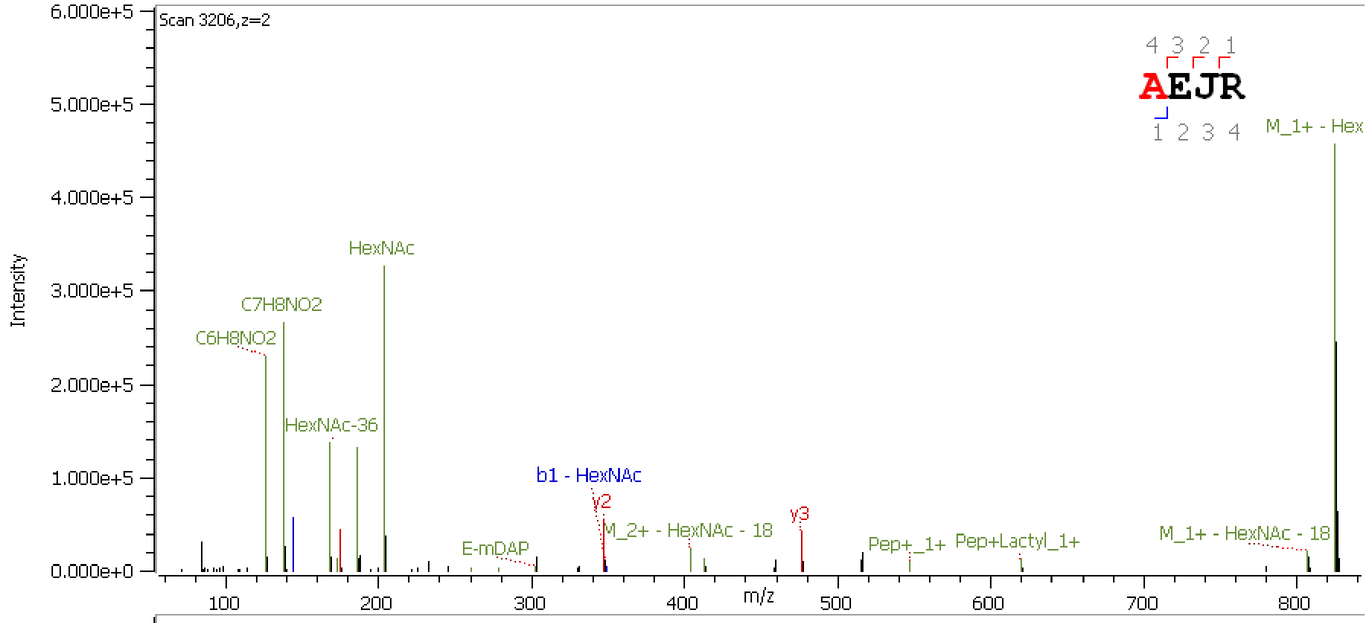

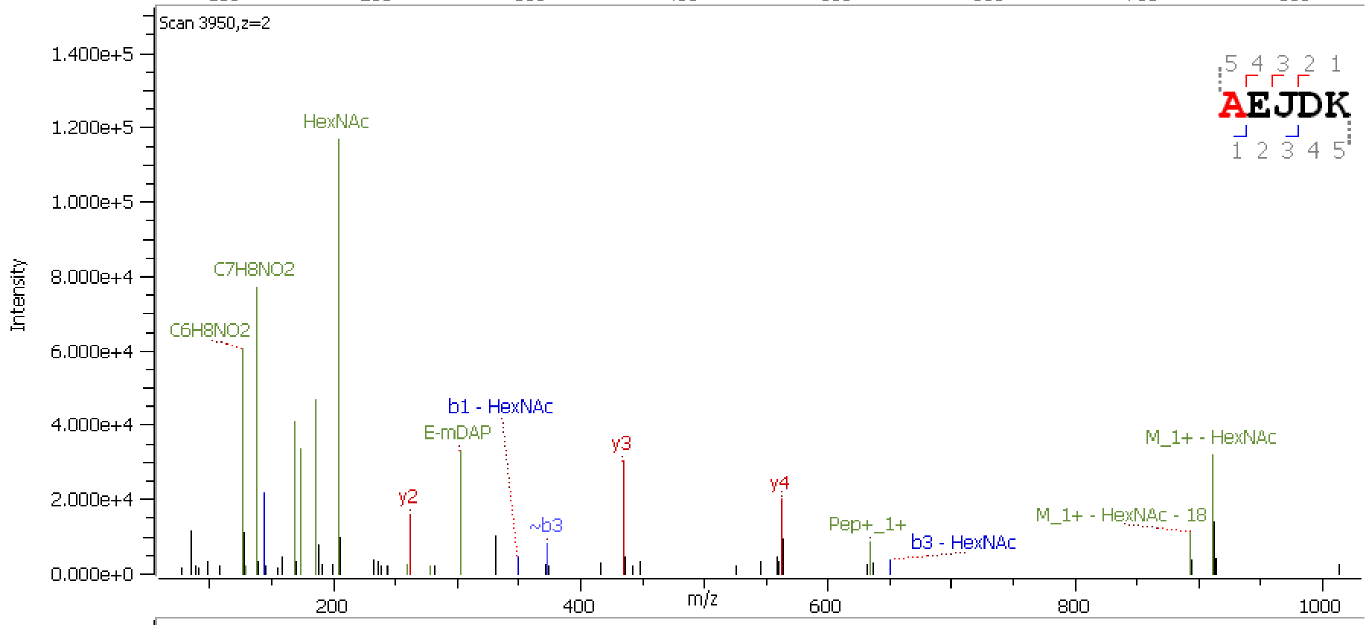

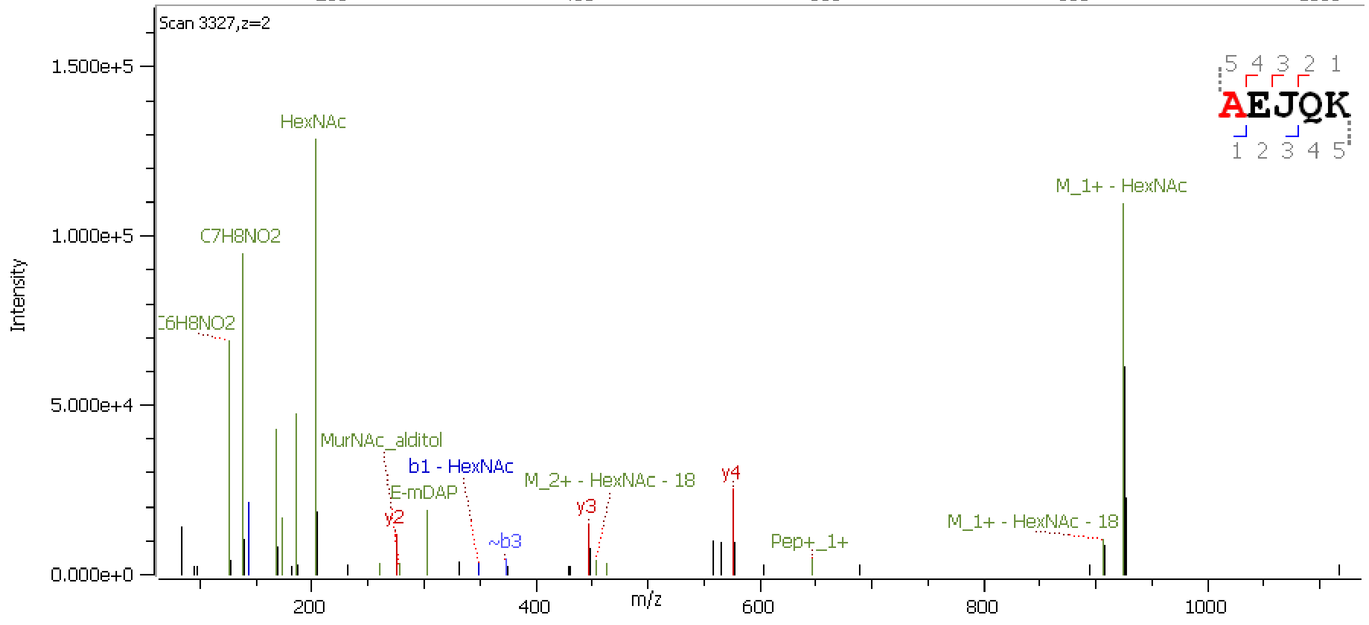

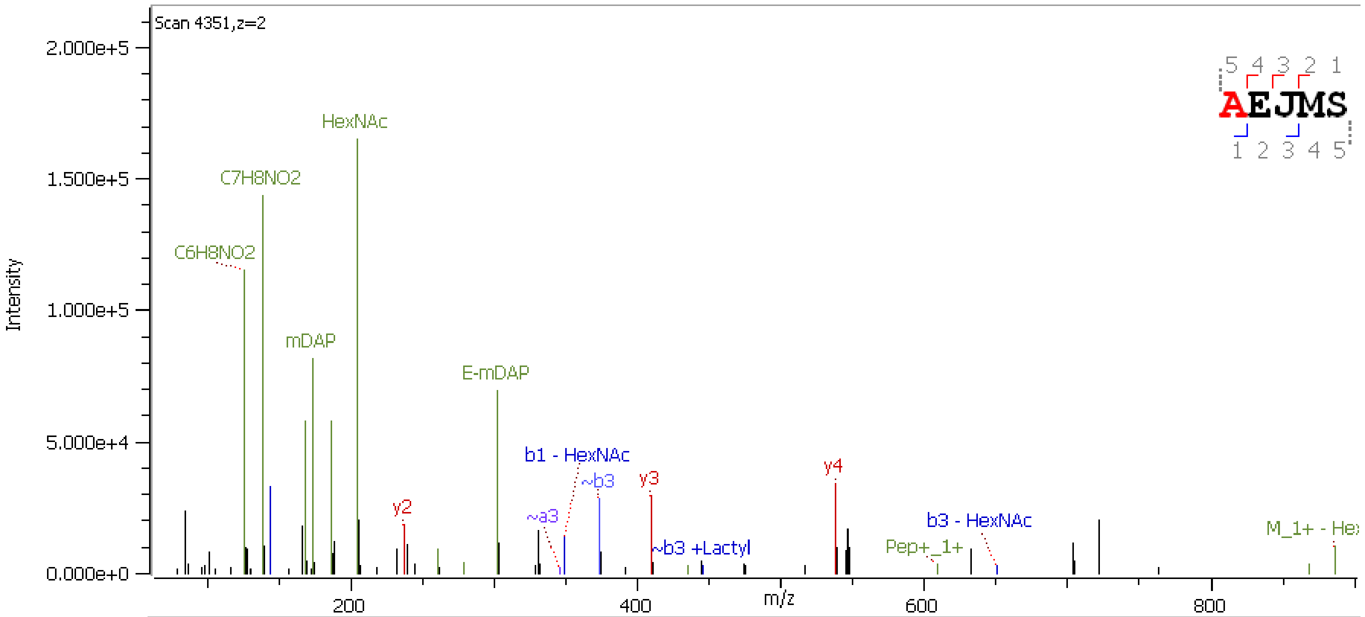
Scan 4351,z=2
Intensity
2.000e+5
1.500e+5
1.000e+5
5.000e+4
0.000e+0
HexNAc
C7H8NO2
C6H8NO2
mDAP
E-mDAP
b1 - HexNAc
~a3
~b3
y2
y3
y4
~b3 +Lactyl
b3 - HexNAc
Pep+_1+
M_1+ - Hex
200
400
m/z
600
800
5 4 3 2 1
AEJMS
1 2 3 4 5

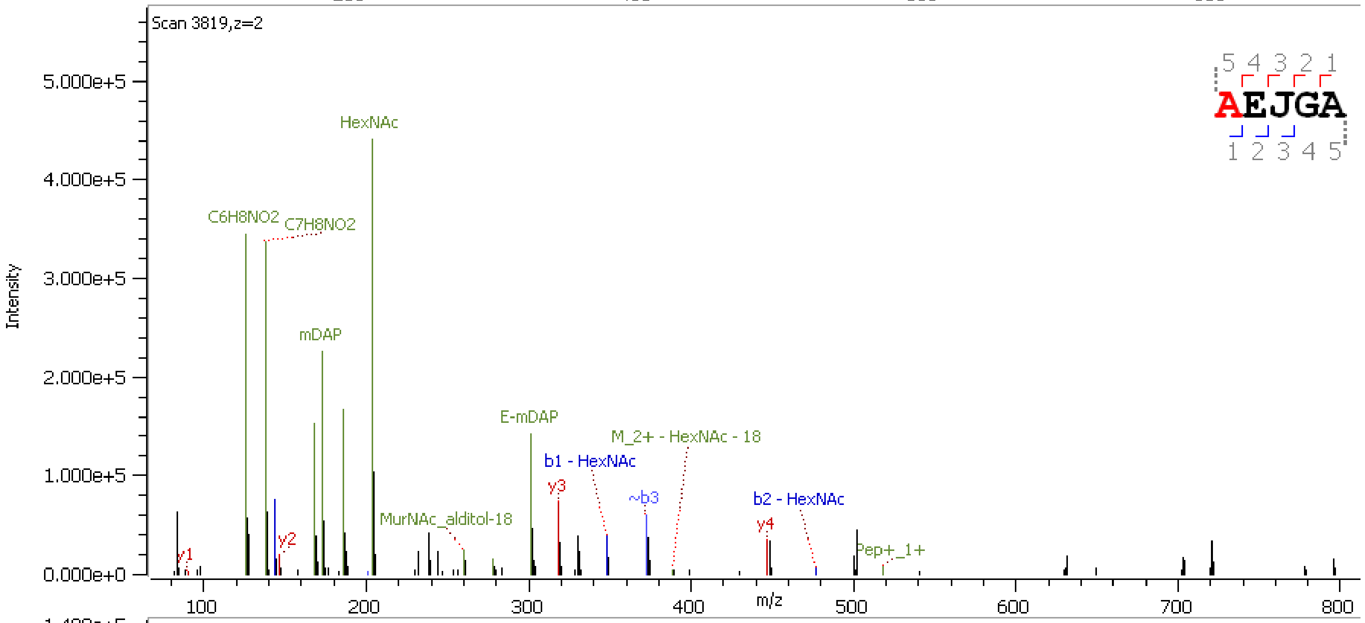
Scan 3819,z=2
Intensity
5.000e+5
4.000e+5
3.000e+5
2.000e+5
1.000e+5
0.000e+0
HexNAc
C6H8NO2
C7H8NO2
mDAP
E-mDAP
M_2+ - HexNAc - 18
b1 - HexNAc
y3
~b3
b2 - HexNAc
y4
MurNAc_alditol-18
y1
y2
Pep+_1+
100
200
300
400
m/z
500
600
700
800
5 4 3 2 1
AEJGA
1 2 3 4 5

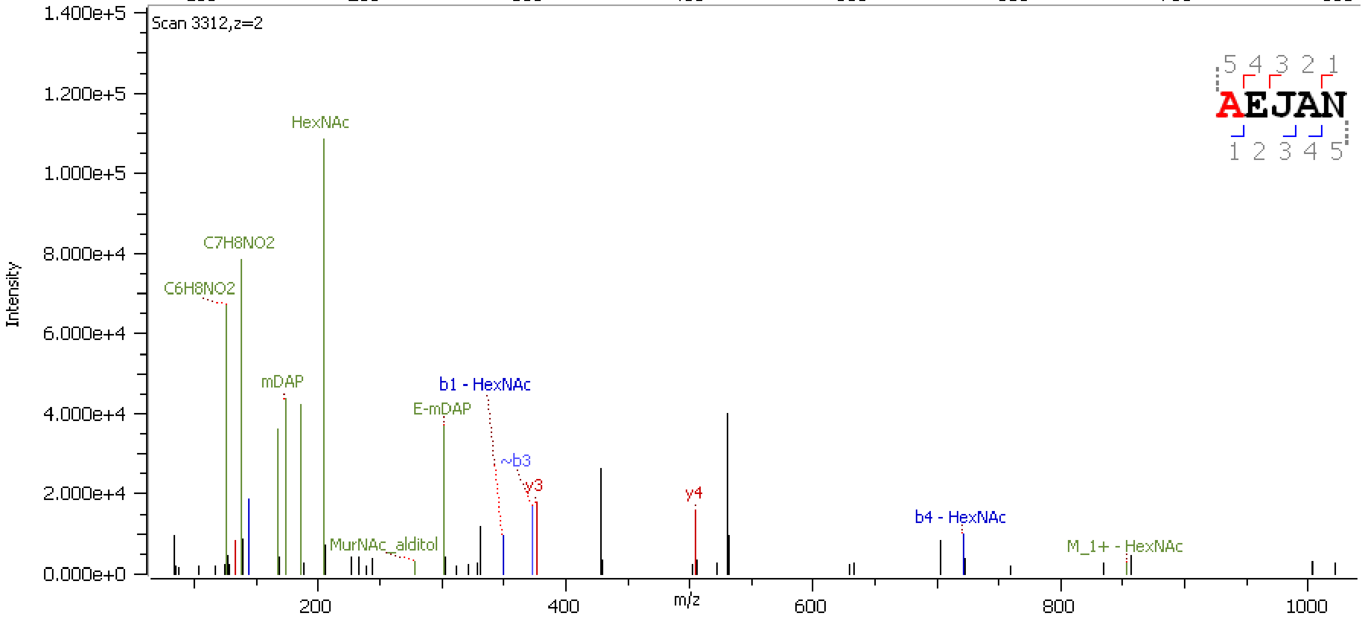
Scan 3312,z=2
Intensity
1.400e+5
1.200e+5
1.000e+5
8.000e+4
6.000e+4
4.000e+4
2.000e+4
0.000e+0
HexNAc
C7H8NO2
C6H8NO2
mDAP
b1 - HexNAc
E-mDAP
~b3
y3
y4
b4 - HexNAc
MurNAc_alditol
M_1+ - HexNAc
200
400
m/z
600
800
1000
5 4 3 2 1
AEJAN
1 2 3 4 5

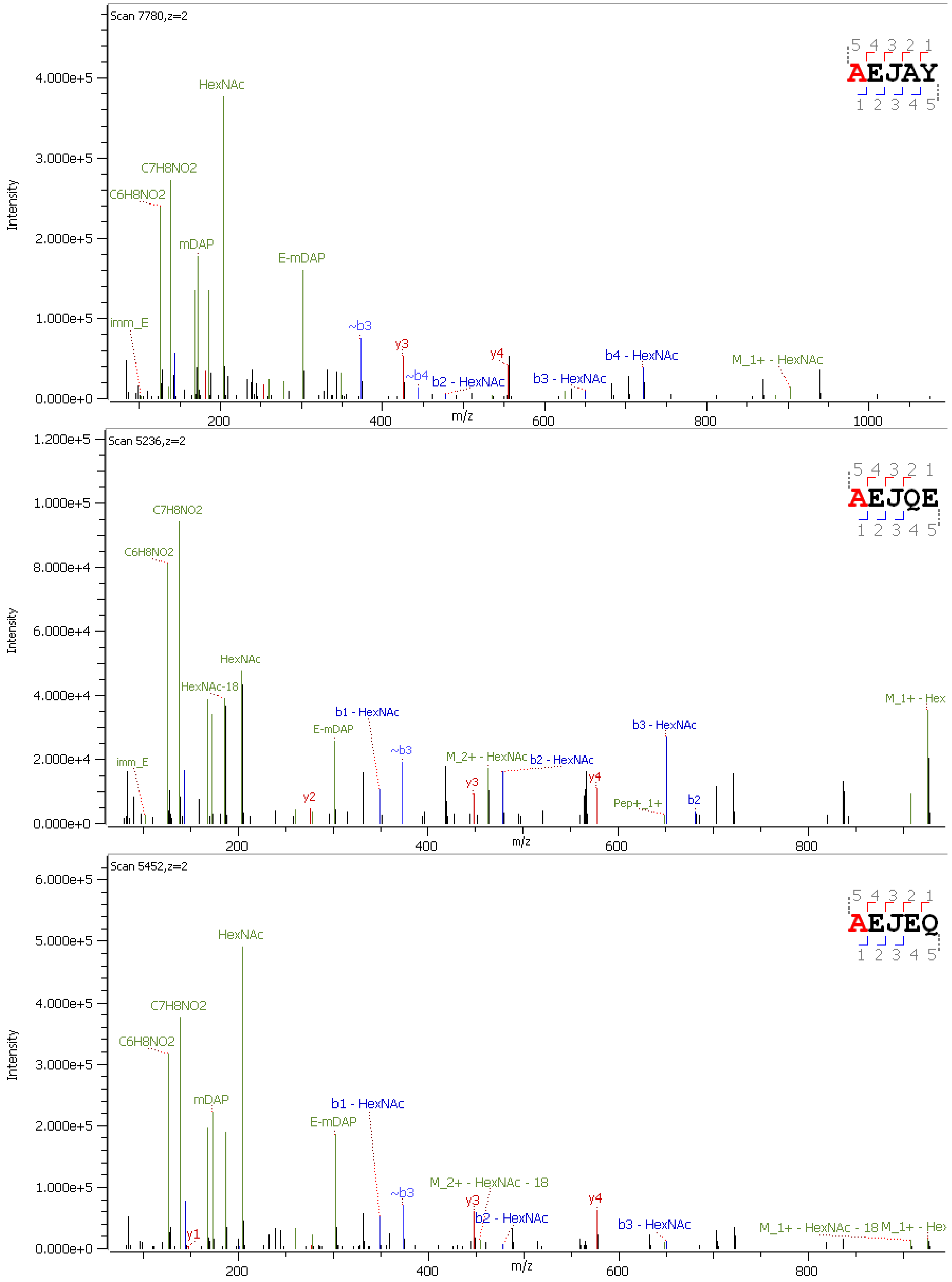
Scan 7780,z=2
AEJAY
Intensity
m/z
HexNAc
C7H8NO2
C6H8NO2
mDAP
E-mDAP
imm_E
~b3
y3
~b4
b2 - HexNAc
y4
b3 - HexNAc
b4 - HexNAc
M_1+ - HexNAc
Scan 5236,z=2
AEJQE
C7H8NO2
C6H8NO2
HexNAc
HexNAc-18
b1 - HexNAc
E-mDAP
~b3
imm_E
y2
M_2+ - HexNAc
b2 - HexNAc
y3
y4
b3 - HexNAc
Pep+_1+
b2
M_1+ - Hex
Scan 5452,z=2
AEJEQ
HexNAc
C7H8NO2
C6H8NO2
mDAP
b1 - HexNAc
E-mDAP
M_2+ - HexNAc - 18
~b3
y3
b2 - HexNAc
y4
b3 - HexNAc
y1
M_1+ - HexNAc - 18
M_1+ - Hex

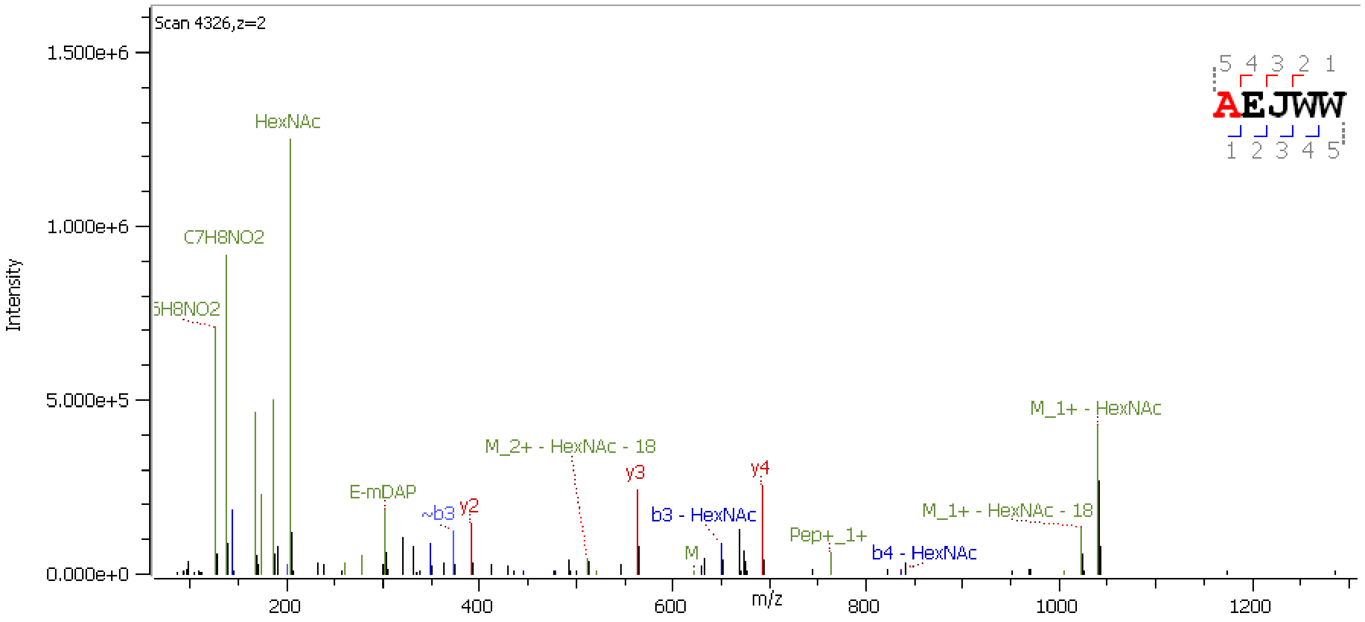

Scan 4326,z=2
Intensity
1.500e+6
1.000e+6
5.000e+5
0.000e+0
HexNAc
C7H8NO2
5H8NO2
E-mDAP
~b3
y2
M_2+ - HexNAc - 18
y3
b3 - HexNAc
M
y4
Pep+_1+
b4 - HexNAc
M_1+ - HexNAc - 18
M_1+ - HexNAc
5 4 3 2 1
AEJWW
1 2 3 4 5
200
400
600
800
1000
1200
m/z

Figure S3. Modification analysis. A, AnhydroMurNAc residues; B, DeAcetylation; C, O-acetylation; D, Amidation; E, Extra gm-; F, Loss of g

A

AnhydroMurNAc (Anh)

Structure	Intensity	% Anh	RT (min)	ΔT (min)*	Comments
gm-AEJA|1<br>gm-AEJA (Anh) |1	1.07E+09<br>3.71E+07	3.5%	9.50 ± 0.03<br>16.12 ± 0.00	6.62	Validated with 1+ and 2+ ions
gm-AEJG|1<br>gm-AEJG (Anh) |1	2.43E+08<br>1.76E+07	7.2%	6.85 ± 0.04<br>14.27 ± 0.01	7.42	Validated with 1+ and 2+ ions
gm-AEJ|1<br>gm-AEJ (Anh) |1	1.64E+08<br>7.90E+07	48.0%	5.48 ± 0.03<br>13.58 ± 0.00	8.09	Validated with 1+ and 2+ ions
gm-AEJAI|1<br>gm-AEJAI (Anh) |1	1.54E+08<br>8.83E+05	0.6%	21.40 ± 0.00<br>28.39 ± 0.02	6.99	No MS/MS data available
gm-AEJF|1<br>gm-AEJF (Anh) |1	1.48E+08<br>4.45E+06	3.0%	21.62 ± 0.00<br>28.70 ± 0.00	7.08	No MS/MS data available
gm-AEJAF (Anh)|1	5.00E+07	0.0%	15.4 ± 0.00	-	Not present
gm-AEJAA|1<br>gm-AEJAA (Anh) |1	6.55E+07<br>5.87E+05	0.9%	10.86 ± 0.02<br>17.26 ± 0.02	6.40	Only present in 2 of 3 datasets
gm-AEJK|1<br>gm-AEJK (Anh) |1	4.60E+07<br>4.32E+06	9.4%	7.71 ± 0.06<br>14.12 ± 0.01	6.41	No MS/MS data available
gm-AEJAG|1,gm-AEJQ|1<br>gm-AEJAG (Anh) |1,gm-AEJQ (Anh) |1	4.61E+07<br>6.53E+05	1.4%	8.80 ± 0.05<br>19.95 ± 6.37	11.15	No signature ions found.
gm-AEJAD|1<br>gm-AEJAD (Anh) |1	2.52E+07<br>2.94E+06	11.7%	9.61 ± 0.04<br>15.82 ± 0.01	6.21	No MS/MS data available

** ΔT is defined as the difference (in min) between the average RT of the unmodified and modified muropeptide

B

DeAcetylation (-Ac)

Structure	Intensity	% (-Ac)	RT (min)	ΔT (min)*	Comments
gm-AEJA|1<br>gm-AEJA (-Ac) |1	1.07E+09<br>3.44E+06	0.3%	9.5 ± 0.03<br>7.8 ± 0.06	-1.7	No signature ions
gm-AEJG|1<br>gm-AEJG (-Ac) |1	2.43E+08<br>1.24E+06	0.5%	6.8 ± 0.04<br>6.4 ± 2.16	-0.4	No MS/MS data available
gm-AEJ|1<br>gm-AEJ (-Ac) |1	1.64E+08<br>7.78E+05	0.5%	5.5 ± 0.03<br>4.1 ± 0.03	-1.3	No MS/MS data available
gm-AEJAI|1<br>gm-AEJAI (-Ac) |1	1.54E+08<br>6.51E+06	4.2%	21.4 ± 0.00<br>25.9 ± 1.67	4.5	RT not compatible with deacetylation (elutes **later** than unmodified monomer)
gm-AEJF|1	1.48E+08	0.0%	21.6 ± 0.00	0.0	Not present
gm-AEJAA|1	6.55E+07	0.0%	10.9 ± 0.02	0.0	Not present
gm-AEJAF|1<br>gm-AEJAF (-Ac) |1	5.00E+07<br>4.65E+05	0.9%	15.4 ± 0.00<br>26.7 ± 0.00	11.3	Only present in 1 of 3 datasets
gm-AEJAG|1,gm-AEJQ|1	4.61E+07	0.0%	8.8 ± 0.05	0.0	Not present
gm-AEJK|1	4.60E+07	0.0%	7.7 ± 0.06	0.0	Not present
gm-AEJAD|1<br>gm-AEJAD (-Ac) |1	2.52E+07<br>7.91E+05	3.1%	9.6 ± 0.04<br>20.9 ± 0.00	11.2	Only present in 1 of 3 datasets

* ΔT is defined as the difference (in min) between the average RT of the unmodified and modified muropeptide

C

O-Acetylation (+Ac)

Structure	Intensity	% O-Ac	RT (min)	ΔT (min)*	Comments
gm-AEJA|1	1.07E+09	0.0%	9.5 ± 0.0	0.0	Not present
gm-AEJG|1	2.43E+08	1.0%	6.8 ± 0.0	7.4	No MS/MS data available
gm-AEJG (+Ac) |1	2.36E+06		14.3 ± 2.3		
gm-AEJ|1	1.64E+08	0.0%	5.5 ± 0.0	0.0	Not present
gm-AEJAI|1	1.54E+08	0.2%	21.4 ± 0.0	5.1	Only present in 2 of 3 datasets
gm-AEJAI (+Ac) |1	3.36E+05		26.5 ± 0.0		
gm-AEJF|1	1.48E+08	0.0%	21.6 ± 0.0	0.0	Not present
gm-AEJAA|1	6.55E+07	0.0%	10.9 ± 0.0	0.0	Not present
gm-AEJAF|1	5.00E+07	2.0%	15.4 ± 0.0	13.5	Only present in 2 of 3 datasets
gm-AEJAF (+Ac) |1	1.00E+06		28.9 ± 0.0		
gm-AEJAG|1,gm-AEJQ|1	4.61E+07	1.1%	8.8 ± 0.1	-2.6	Only present in 1 of 3 datasets
gm-AEJAG (+Ac) |1,gm-AEJQ (+Ac) |1	5.17E+05		6.2 ± 0.0		
gm-AEJK|1	4.60E+07	0.0%	7.7 ± 0.1	0.0	Not present
gm-AEJAD|1	2.52E+07	2.3%	9.6 ± 0.0	7.3	Only present in 1 of 3 datasets
gm-AEJAD (+Ac) |1	5.73E+05		16.9 ± 0.0		

* ΔT is defined as the difference (in min) between the average RT of the unmodified and modified muropeptide

D

Amidation (Am)

Structure	Intensity	% (+NH$_2$)	RT (min)	ΔT (min)*	Comments
gm-AEJA|1	1.07E+09	0.2%	9.5 ± 0.0	2.2	RT not compatible with amidation (elutes **later** than unmodified monomer)
gm-AEJA (Am) |1	1.90E+06		11.7 ± 0.1		
gm-AEJG|1	2.43E+08	0.2%	6.8 ± 0.0	3.9	RT not compatible with amidation (elutes **later** than unmodified monomer)
gm-AEJG (Am) |1	5.57E+05		10.7 ± 0.0		
gm-AEJ|1	1.64E+08	0.5%	5.5 ± 0.0	-1.0	No MS/MS data available
gm-AEJ (Am) |1	7.82E+05		4.4 ± 0.0		
gm-AEJAI|1	1.54E+08	0.8%	21.4 ± 0.0	4.9	RT not compatible with amidation (elutes **later** than unmodified monomer)
gm-AEJAI (Am) |1	1.26E+06		26.3 ± 0.0		
gm-AEJF|1	1.48E+08	0.3%	21.6 ± 0.0	2.0	RT not compatible with amidation (elutes **later** than unmodified monomer)
gm-AEJF (Am) |1	4.10E+05		23.7 ± 9.1		
gm-AEJAA|1	6.55E+07	3.2%	10.9 ± 0.0	19.3	RT not compatible with amidation (elutes **later** than unmodified monomer)
gm-AEJAA (Am) |1	2.07E+06		30.1 ± 1.6		
gm-AEJAF|1	5.00E+07	0.0%	15.4 ± 0.0	-	No modification found
gm-AEJAG|1,gm-AEJQ|1	4.61E+07	1.5%	8.8 ± 0.1	15.0	RT not compatible with amidation (elutes **later** than unmodified monomer)
gm-AEJAG (Am) |1,gm-AEJQ (Am) |1	6.81E+05		23.8 ± 0.0		
gm-AEJK|1	4.60E+07	2.1%	7.7 ± 0.1	13.0	RT not compatible with amidation (elutes **later** than unmodified monomer)
gm-AEJK (Am) |1	9.45E+05		20.7 ± 0.0		
gm-AEJAD|1	2.52E+07	4.6%	9.6 ± 0.0	-1.9	No MS/MS data available
gm-AEJAD (Am) |1	1.16E+06		7.7 ± 0.0		

* ΔT is defined as the difference (in min) between the average RT of the unmodified and modified muropeptide

E

Extra-gm (gm-)

Structure	Intensity	% Extra gm	RT (min)	ΔT (min)*	Comments
gm-AEJA|1	1.07E+09	0.4%	9.5 ± 0.0	3.1	No convincing signature ions

gm-gm-AEJA|1	4.17E+06		12.6 ± 0.0	[illegible]	No convincing signature ions
gm-AEJG|1<br>gm-gm-AEJG|1	2.43E+08<br>6.03E+05	0.2%	6.8 ± 0.0<br>11.3 ± 0.0	4.5	No MS/MS data available
gm-AEJ|1<br>gm-gm-AEJ|1	1.64E+08<br>7.59E+05	0.5%	5.5 ± 0.0<br>10.8 ± 0.0	5.3	No MS/MS data available
gm-AEJAI|1<br>gm-gm-AEJAI|1	1.54E+08<br>2.12E+07	13.8%	21.4 ± 0.0<br>31.5 ± 0.0	10.1	No MS/MS data available
gm-AEJF|1	1.48E+08	-	21.6 ± 0.1	-	Not present
gm-AEJAA|1	6.55E+07	-	10.9 ± 0.1	-	Not present
gm-AEJAF|1<br>gm-gm-AEJAF|1	5.00E+07<br>6.18E+06	12.4%	15.4 ± 0.0<br>31.4 ± 1.8	16.0	Not validated, too low coverage (13%)
gm-AEJAG|1,gm-AEJQ|1	4.61E+07	-	8.8 ± 0.0	-	Not present
gm-AEJK|1<br>gm-gm-AEJK|1	4.60E+07<br>7.23E+05	1.6%	7.7 ± 0.0<br>17.4 ± 0.0	9.7	No MS/MS data available
gm-AEJAD|1	2.52E+07	-	9.6 ± 0.0	-	Not present

* ΔT is defined as the difference (in min) between the average RT of the unmodified and modified muropeptide

F

Loss of GlcNAc (m-)

Structure	Intensity	Loss of GlcNA	RT (min)	ΔT (min)*	Comments
gm-AEJA|1<br>m-AEJA|1	1.48E+09<br>4.38E+07	3.0%	9.5 ± 0.0<br>8.6 ± 0.1	-0.9	No MS/MS data available
gm-AEJG|1	3.70E+08	-	6.8 ± 0.0	-	Not present
gm-AEJ|1<br>m-AEJ|1	2.65E+08<br>5.09E+05	0.2%	5.5 ± 0.0<br>23.7 ± 0.0	18.2	RT not compatible with loss of GlcNAc (elutes **later** than unmodified monomer)
gm-AEJF|1<br>m-AEJF|1	2.12E+08<br>9.09E+06	4.3%	21.6 ± 0.0<br>28.7 ± 0.0	7.1	RT not compatible with loss of GlcNAc (elutes **later** than unmodified monomer)
gm-AEJAI|1<br>m-AEJAI|1	1.94E+08<br>3.16E+06	1.6%	21.4 ± 0.0<br>16.2 ± 0.0	-5.3	Not validated low coverage (20%)
gm-AEJAA|1	8.89E+07	-	10.9 ± 1.4	-	Not present
gm-AEJAG|1,gm-AEJQ|1	6.32E+07	-	8.8 ± 0.1	-	Not present
gm-AEJK|1<br>m-AEJK|1	5.65E+07<br>6.91E+05	1.2%	7.7 ± 0.1<br>14.1 ± 0.0	6.4	RT not compatible with loss of GlcNAc (elutes **later** than unmodified monomer)
gm-AEJAF|1	5.32E+07	-	18.1 ± 0.0	-	Not present
gm-AEJAD|1	3.07E+07	-	9.6 ± 0.0	-	Not present

* ΔT is defined as the difference (in min) between the average RT of the unmodified and modified muropeptide

Figure S4. Putative beta-barrel proteins encoded by *Rhizobium leguminosarum* bv. *viciae* strain 3841

>pRL90069 Q1M8N4|Q1M8N4_RHIL3 Conserved hypothetical exported protein
MKKILATAFAAVSLTFVGAAAVNAADLGTRTYEEPDLRNGVKIGYLTCDIGGGTGYVLGSSKEADCIFQSTVGNELSDRYTGEMRKLGIDLGFTTRSRLI
WAVFAPTAGYHRGSLAGLYVGATAEATLGAGVGANLLVGGTSGSIHLQTVSLTGQLGLNVAAGSASMTLTAAN

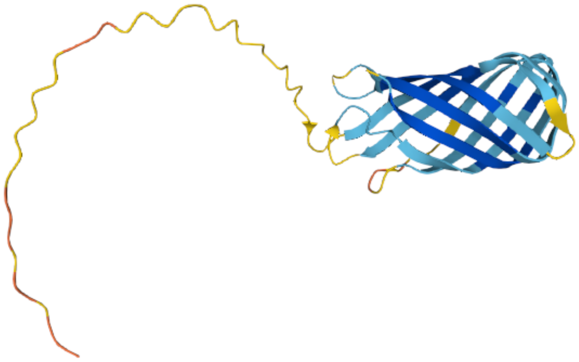

>RL0868 (LpxQ) Q1MKZ0_RHIL3 Lipid A oxidase
MTYALRSSASLLAGIAFLTICSAVSASAEDLQFSIYGGYQTAPHSGVDLSDGTSFTAGWEGKSFGSPPYYGARVTWWLENFNKPNWGISLDYTHDKVYAD
DDTLAKAGWSHFEFTDGLNLITVNGLYRFQDPTRRWTPYLGAGIGVNIPHVEVIRPEGKTWAYEFGGVTLQAQAGVDFKVTERWSTFVEYKGTYSRIDVP
IDSGVDLKTNIFTNAVNVGVSFHW

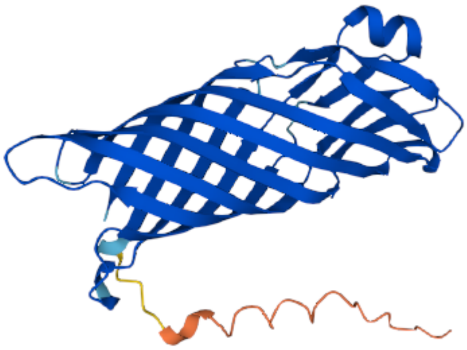

>RL2752 OmpA family protein Q1MFN3_RHIL3
MGMKSRLFASAAFPLLSLSLALQPATAMAAVRDVATQASAVQQAEQGSFEVAQDAPSEEELLKKKRKQQEEAPAEKPAQQEAPAEKPKAERKEAPAPEPK
AEPEPPKAEAAPKEEPAPQPESKPARKAKSEAQPEAKPEAEQPVTQEKPKKPKKPEAQQAEPEQQPAAKEAQPEAEQAQPEAKPEGGKRDKGQDKAQGRD
KGKGKAEKEAQPAAPDAVTPTAESAKPEATPEAKPAAEAPAEKKPAKGETAAPADKAPTDKATEGKATEGKATEDKAAAPEAAPAEKPKDGTAAKPAGEQ
PAGAQPAAPATDTAQPLPDASGGQQVEQAIPAPEKVSPEELERRKKIAADPAKSSETVVLPVENGAAVLDSDKDADRSKGREGRRDRDRQRADSQEVKVP
TSDADAQALSGAKAPAPVKLEAVTREKGRKLDERPRFVRPDGARFDDRGSDDSRVIIQYDNRTIVRGDDDRRFLRDGERPSYEELSGDRYRETITRPEGY
RIVTIRNRYGDIIQRSRVDARGREDVLYYSQDLYDDPDRDYFEDPGADLPPMRLRVPLSDYIIDTRSDPNRDYYEFLSEPPVEPVERVYSLDEVKYSARI
RDKVRRIDLDTITFATGSADIPMTQARTLRKVADAISQVLEKDPSETFLIEGHTDAVGSDQSNLILSDQRAESVANVLSDVYGIAPENLATQGYGESYLK
VNTSAPEQENRRVTIRRVTALVRPVAANK*

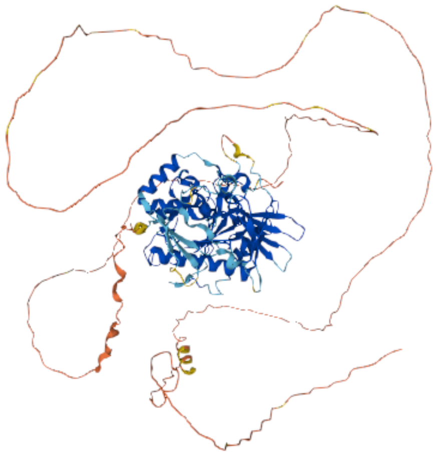

>RL3165 Q1MEH4_RHIL3 Conserved hypothetical TPR repeat receptor protein
MLGRWKNICRVGMALAFAVPGFGLPAMADPVPRATPVAGSVIARKIGEEVRFIDVSNWRVVDINQDLLTGDVLRTNANGQLAIVF
SDHTQVRLGRNSSLQVKKMAAGGDTVLNLQSGTIWARAERGGQGLTVETPAAAAAIRGTDWTMTVEGAKTSMVVLEGRVALSNPQ
GSVEVNEGEGAVATIGQAPSKIISVNPDDREQMLFYLDLRDGFDLMPTSPLRADRMATERRRLLGLPPERRTTEDWLELAEVQSA
FDGRQAAAATLQNIRGRKLTTAQQARVDLIDATIAGSEKRYGDAAKLFQKALPHLDATRRNMAQYGGYFARSLADPAHAEPPPAG
TTGPYGAIMQAYTAGFLENPRAAIDIIRKAEQRYPDDPTLPAVRAQLAQLTDDREQMKEAIERSLSLDPDHPMALSARAGYKAIY
ESDIDGALADLNRAIALAPGASGTLNSLGLLQSSRDANGEAEQAFKKAIELDPQDPLLRANLSILYLDQGRMKEAKHEIDTAIAL
DPSFDIALLARGRYYLQTGERDRALQDLLAASTANPAHSQSQLMLAAAHYEKGDRIPSQQALDNADRLDKNDPVISAFRTAVDID
DYDADGAIRNAQEFLRRSRARGGDYSGLGANASAGSTLNDAFRLQGLDAWGRYYGDAVFDPFNGTGYIDQSIKGSIFPFVNATSF
SDDNIIQNRGNASSYSSFIQGLLLSPHMLSGRSRSATLFDVPFIEGSLGGGINSVDGHTRRIGEADIQGYSNETIPISFYGNLTW
EELALDRDYQDFGGVQTDNKLLSANGYLTATVTPDDRVVAFVNHGKNDGTLNALSSNTGFMELLFRVPIPLPLYTTEETERESTY
AGIGWSHTFAYENVLNGALLYSGSKSNTNSALDVDLDPVFIGRGVPFIIPFTNVTQETESQTYIGALSHSIGAGPLTFRYGIEGG

WMDASSTVDATLLGLTAPTDRTENTIDIGRGYIDVLHEITPDLKGEYALFATRLEGDGIDISRLEPRFGLAWAPVQNHWLRAAFM
RQSFDIGIPTLAPIGVLGLQANQFSANPQGYTDTVALQWDAEWTDRFFTSVEYQHQELHDFAIDFPLISLPSDTSLPISRGSIDR
AAVTANVVLGHGFGLSATYAYMDSENRDPLEPIYGGPLPFIPQNSGQIALTWVNEAKVKATVAANYIGERDGDRFGTKLDDYWSL
DAHLVWEPFDKRIELEAAAYNLLDEDFEITPGVPGWGRAFKGTLKVRF

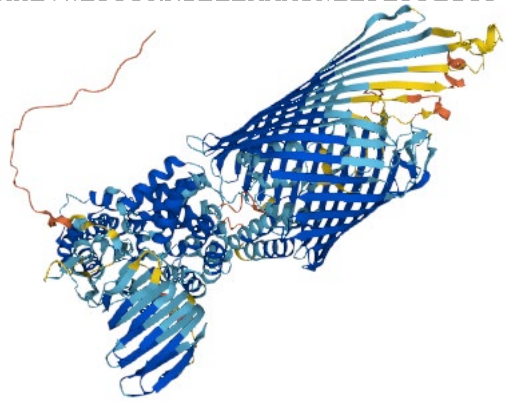

>RL3968 (Pal) Q1MC73_RHIL3 Peptidoglycan-associated protein
MSRIHTPAMSRMQNFARNPVMIALIAGLALASCAKKNVPNSAGDLGLGAGAGAATPGSAQDFTVNVGDRIFFDTDSSSIRADASQTLDRQAQWLGRYPNY
QITVEGHADERGTREYNLALGARRAAAAKDYLASRGVPAQRLKTISYGKERPVAVCDDISCWSQNRRAVTVLGGAGM*

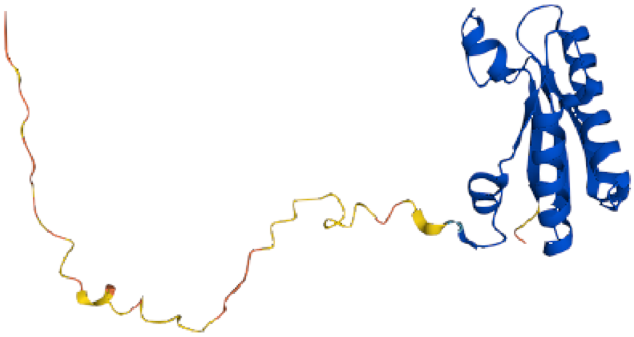

>RL4133 Q1MBR1_RHIL3 Conserved hypothetical exported protein
MFKQTMIAAAALTAAAWASPAGAENYVTLGRLVCGSDGGQGLIVTSQKNLICTYTPSAGGAKAVYAGKIEKFGLDIGQTGKSVMIWQVLAKTGTDIP
QFALAGEYYGIGADASIGAGAGAKVIAGGTDKAFMLQPLNVQAQEGLNLAIGVEKMTLVPGET

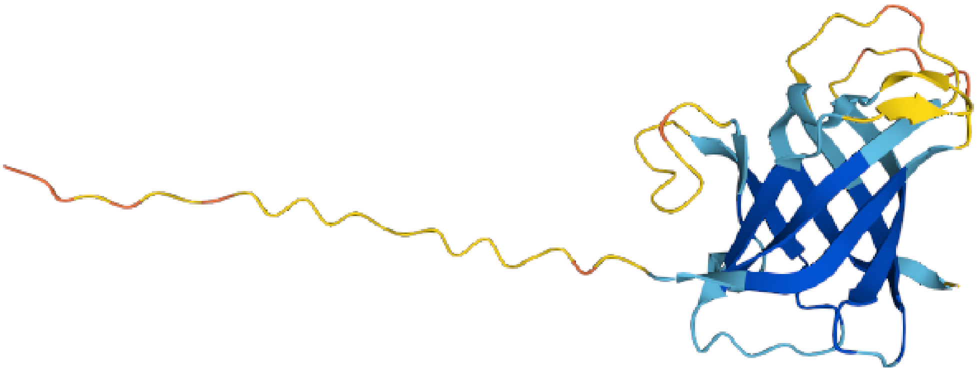

>RL (RopA1) Q1MFL0_RHIL3 Porin
MNIRMVLLASAAAFAASTPVLAADAIVAAEPEPVEYVRVCDAYGTGYFYIPGTETCLKIEGYIRFQVDVGDQPLNGSESNDSDWDARTRGQVQFTAKSDT
EYGPLTGVIVMQFNADNATDQDAILDSAYLDVAGFRAGLFYSWWDDGLSGETDDIGSIVTLHNSLRYQYESGTFYAGISVDELEDGFYKSDEEPNNVGVA
FGVGGTAGAFSYQITGGWDFDNEDGAIRAMGTVDIGPGTLGLAAVYSSGPNSYYSAAEWAVAAEYAIKATDKLKITPGVQYYGDYYVDGDDFSGGDAWKV
GLTVDYQIVDNFYAKASVQYLDPEDEDDSTAGYFRLQRSF

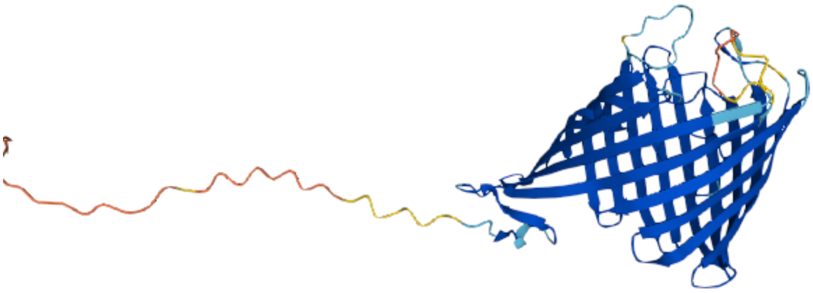

>RL (RopA2) Q1MJ66_RHIL3 Porin
MNIKSLLLGSAAALAVVSGAQAADAIVAAEPEPVEYVRVCDAYGTGYFYIPGTETCLKINGYIRFQVDVGDQPLNGSESNDSDWDARTRGQVQFTAKSDT
EYGPLTGVIVMQFNADNATDQSAKLDSAYLDIAGFRAGLFYSWWDDGLSGETDDIGSPVTLHNSIRYQYETDAFYAGISVDELEDGYYKADEEPNNVGVA

VGLGGKAGAFSYQITAGYDVDNEDGAVRAMGTVDIGPGTLGLAAVYATGPSSYYTKAEWAVAAEYAIKATDKLKITPGVQYYSNYGITDDDFDDGDAWKV
GLTVDYQIVDNFYAKASVQYLDPEDDDDSTSGYFRLQRSF

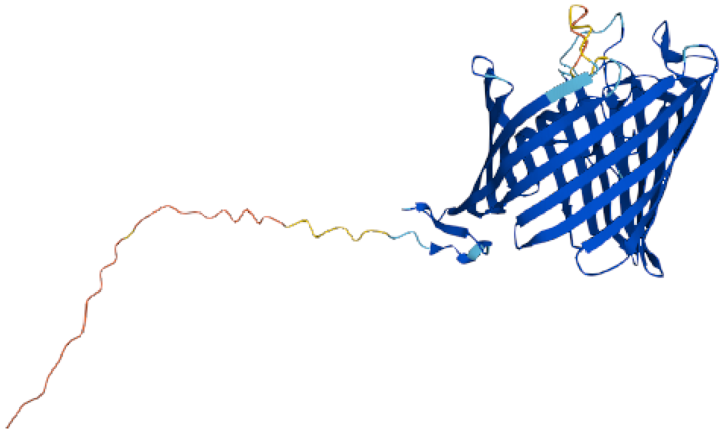

>RL (RopA3) Q1M611_RHIL3 Porin
MNIRTILFASVAALAAASGARAADAIVAAEPEPVEYVRVCDAYGTGYFYIPGTETCLSIGGYIRTEVRFGEQISGDSDVNFWTRGQVTFQTKNDTEYGTL
TGVITLRYNVDNASDQEALLDEGYLDIAGFRAGKLYSWWDDDMSGETDTLASNETTHNSIRYQYENGAFAAGISVDELEEDYDTKPGEGPNNFGVAGQVS
YKAGAISAYLLAGYDTDTSEVAVRGIVYADIGPGTLGIAGVWASGANYYYEESEWTIAAEYALKVNDKWSVTPGFQYFENIALEADGNGFTGGSAYTTGV
TIDYQIVEDLRSKLSVQYHDEDEGDDEVFGFLRFQRDF

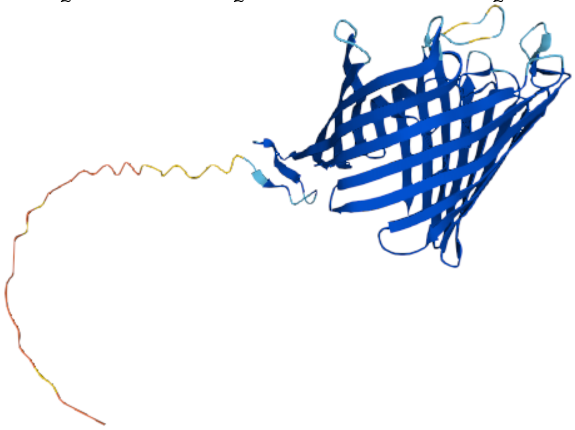

>RL1589 (RopB) Q1MIX6_RHIL3 Putative RopB outer membrane protein
MRVLIAGLMASVFAIAGVSAAQAADAVDQVPEAPVAQEAPVKPAGNWEGFYLGGAGTYNMGDFGSDRHTYGFGGQVFTGYNWQQGQIVYGVESDLGYSGD
DVSSGGVKNKYGWNGSVRGRVGYDMNPFLLYGTAGLAIGDVKVSDDTSDESKTNFGYTVGAGVEAFVTNNITTRLEYRYTDYQSKDYDLDSGSFSRGYDE
NSVKLGIGVKF

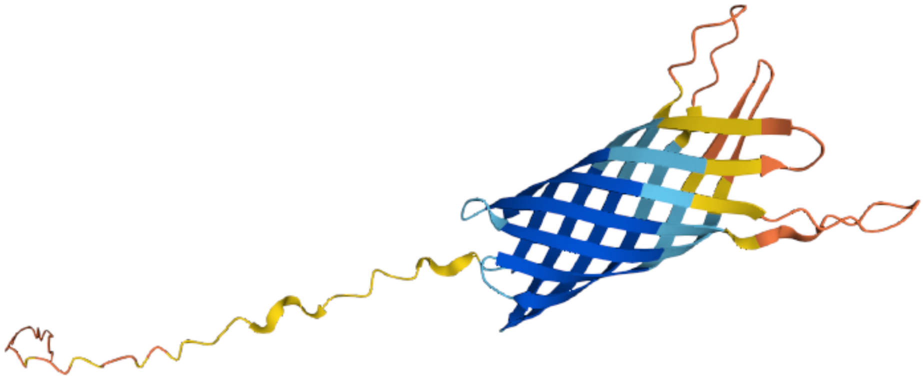

A

RopA1	MNIRMVLLASAAAFAASTPVLA	ADAIVAA
RopA2	MNIKSLLLGSAAALAVVSGAQA	ADAIVAA
RopA3	MNIRTILFASVAALAAASGARA	ADAIVAA
RopB	MRVLIAGLMASVFAIAGVSAAQA	ADAVDQVPEAPVAQ
pRL90069	MKKILATAFAAVSLTFVGAAAVNA	ADLGTR

B

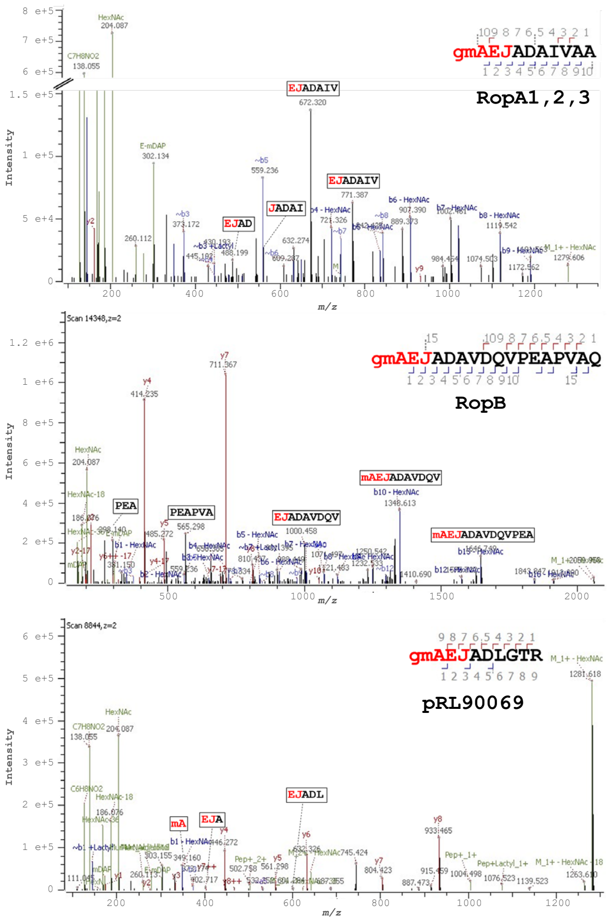

Figure S5. The existence of β-barrel anchoring scars can be confirmed via MS/MS. (A) N-terminal sequences of five putative peptidoglycan-anchored β-barrel proteins; the arrow shows the predicted signal peptide cleavage site. **(B)** Example MS/MS spectra confirm the N-terminal anchoring of these β-barrel proteins. Ions corresponding to internal fragments that were manually annotated are boxed.

Table S1: Residues built into PGFinder.

Symbol	Name	Formula	Monoisotopic Mass
g	*N*-Acetylglucosamine	$C_8H_{15}NO_6$	221.089937
m	*N*-Acetylmuramic Acid	$C_{11}H_{19}NO_8$	293.111067
x	Unknown Monosaccharide		0
A	Alanine	$C_3H_7NO_2$	89.047678
B	Diaminobutyric Acid	$C_4H_{10}N_2O_2$	118.074228
C	Cysteine	$C_3H_7NO_2S$	121.019750
D	Aspartic Acid	$C_4H_7NO_4$	133.037508
E	Glutamic Acid	$C_5H_9NO_4$	147.053158
F	Phenylalanine	$C_9H_{11}NO_2$	165.078979
G	Glycine	$C_2H_5NO_2$	75.0320028
H	Histidine	$C_6H_9N_3O_2$	155.069477
I	Isoleucine	$C_6H_{13}NO_2$	131.094629
J	Diaminopimelic Acid	$C_7H_{14}N_2O_4$	190.095357
K	Lysine	$C_6H_{14}N_2O_2$	146.105528
L	Leucine	$C_6H_{13}NO_2$	131.094629
M	Methionine	$C_5H_{11}NO_2S$	149.051050
N	Asparagine	$C_4H_8N_2O_3$	132.053492
O	Ornithine	$C_5H_{12}N_2O_2$	132.089878
P	Proline	$C_5H_9NO_2$	115.063329
Q	Glutamine	$C_5H_{10}N_2O_3$	146.069142
R	Arginine	$C_6H_{14}N_4O_2$	174.111676
S	Serine	$C_3H_7NO_3$	105.042593
T	Threonine	$C_4H_9NO_3$	119.058243
U	Homoserine	$C_4H_9NO_3$	119.058243
V	Valine	$C_5H_{11}NO_2$	117.078979
W	Tryptophan	$C_{11}H_{12}N_2O_2$	204.089878
X	Unknown Amino Acid		0
Y	Tyrosine	$C_9H_{11}NO_3$	181.073893
Z	Threo-3-Hydroxyglutamic	$C_5H_9NO_5$	163.048072

Both “x” and “X” can be used as wild-cards with the PGLang offset syntax — e.g. “X(+C5H12N4O3)”.

Table S2: Modifications built into PGFinder.

Symbol	Name	Lost Atoms	Gained Atoms	Net Monoisotopic Mass	Targeted Functional Groups
Ac	*O*-Acetylation	H	C_2H_3O	42.010565	"Hydroxyl" at="6-Position"
Am	Amidation	OH	NH_2	-0.984016	"Carboxyl" at="Sidechain"
Anh	1,6-Anhydro	H_2O	-	-18.010565	"Hydroxyl" at="Reducing End" of="*N*-Acetylmuramic Acid"
DeAc	De-*N*-Acetylation	C_2H_3O	H	-42.010565	"Acetyl" at="Secondary Amide"
Glyc	Glycolylation	CH_3	CH_2OH	15.994915	"Acetyl" at="Secondary Amide" of="*N*-Acetylmuramic Acid"
Poly	Wall Polymer Linkage	H	PO_3	77.950681	"Hydroxyl" at="6-Position"
Red	Reduced	-	H_2	2.015650	"Hydroxyl" at="Reducing End"

Note that specific modifications can only target specific residues / functional groups. The groups each modification targets are described in the last column.

Table S3. Database 1 (DB_1)

Structure	Monoisotopicmass
gm-AE|1	698.28590
gm-AEJ|1	870.37069
gm-AEJA|1	941.40783
gm-AEJAA|1	1012.44497
gm-AEJC|1	973.37988
gm-AEJCA|1	1044.41702
gm-AEJCC|1	1076.38907
gm-AEJCE|1	1102.42247
gm-AEJCG|1	1030.40134
gm-AEJCH|1	1110.43879
gm-AEJCK|1	1101.47484
gm-AEJCN|1	1087.42281
gm-AEJCP|1	1070.43264
gm-AEJCS|1	1060.41191
gm-AEJD|1	985.39763
gm-AEJDA|1	1056.43477
gm-AEJDC|1	1088.40682
gm-AEJDD|1	1100.42457
gm-AEJDE|1	1114.44022
gm-AEJDG|1	1042.41909
gm-AEJDH|1	1122.45654
gm-AEJDN|1	1099.44056
gm-AEJDP|1	1082.45039
gm-AEJDQ|1	1113.45621
gm-AEJDT|1	1086.44531
gm-AEJDW|1	1171.47694
gm-AEJE|1	999.41328
gm-AEJEA|1	1070.45042
gm-AEJED|1	1114.44022
gm-AEJEE|1	1128.45587
gm-AEJEG|1	1056.43474
gm-AEJEH|1	1136.47219
gm-AEJEI|1	1112.49734
gm-AEJEN|1	1113.45621
gm-AEJES|1	1086.44531
gm-AEJEV|1	1098.48169
gm-AEJF|1	1017.43910
gm-AEJFA|1	1088.47624
gm-AEJFC|1	1120.44829
gm-AEJFD|1	1132.46604
gm-AEJFE|1	1146.48169
gm-AEJFF|1	1164.50751
gm-AEJFG|1	1074.46056
gm-AEJFH|1	1154.49801
gm-AEJFI|1	1130.52316
gm-AEJFK|1	1145.53406
gm-AEJFN|1	1131.48203
gm-AEJFP|1	1114.49186
gm-AEJFQ|1	1145.49768
gm-AEJFR|1	1173.54021
gm-AEJFS|1	1104.47113
gm-AEJFT|1	1118.48678
gm-AEJFV|1	1116.50751
gm-AEJFW|1	1203.51841
gm-AEJFY|1	1180.50243
gm-AEJG|1	927.39215
gm-AEJGA|1	998.42917
gm-AEJGG|1	984.41361
gm-AEJGH|1	1064.45106
gm-AEJGM|1	1058.43264
gm-AEJGP|1	1024.44491
gm-AEJGQ|1	1055.45073
gm-AEJGR|1	1083.49326
gm-AEJGS|1	1014.42418
gm-AEJGV|1	1026.46056
gm-AEJGW|1	1113.47146
gm-AEJH|1	1007.42960
gm-AEJHA|1	1078.46674

gm-AEJHE|1	1136.47219
gm-AEJHH|1	1144.48851
gm-AEJHP|1	1104.48236
gm-AEJHQ|1	1135.48818
gm-AEJHS|1	1094.46163
gm-AEJHT|1	1108.47728
gm-AEJHW|1	1193.50891
gm-AEJI|1	983.45475
gm-AEJIA|1	1054.49189
gm-AEJID|1	1098.48169
gm-AEJIE|1	1112.49734
gm-AEJIH|1	1120.51366
gm-AEJII|1	1096.53881
gm-AEJIK|1	1111.54971
gm-AEJIN|1	1097.49768
gm-AEJIP|1	1080.50751
gm-AEJIQ|1	1111.51333
gm-AEJIS|1	1070.48678
gm-AEJIT|1	1084.50243
gm-AEJIW|1	1169.53406
gm-AEJK|1	998.46565
gm-AEJKA|1	1069.50279
gm-AEJKD|1	1113.49259
gm-AEJKE|1	1127.50824
gm-AEJKG|1	1055.48711
gm-AEJKH|1	1135.52456
gm-AEJKI|1	1111.54971
gm-AEJKK|1	1126.56061
gm-AEJKN|1	1112.50858
gm-AEJKP|1	1095.51841
gm-AEJKQ|1	1126.52423
gm-AEJKR|1	1154.56676
gm-AEJKT|1	1099.51333
gm-AEJKW|1	1184.54496
gm-AEJL|1	983.45475
gm-AEJLC|1	1086.46394
gm-AEJLG|1	1040.47621
gm-AEJLV|1	1082.52316
gm-AEJM|1	1001.41118
gm-AEJMA|1	1072.44832
gm-AEJMC|1	1104.42037
gm-AEJMD|1	1116.43812
gm-AEJME|1	1130.45377
gm-AEJMF|1	1148.47959
gm-AEJMH|1	1138.47009
gm-AEJMI|1	1114.49524
gm-AEJMK|1	1129.50614
gm-AEJMM|1	1132.45167
gm-AEJMN|1	1115.45411
gm-AEJMP|1	1098.46394
gm-AEJMQ|1	1129.46976
gm-AEJMS|1	1088.44321
gm-AEJMT|1	1102.45886
gm-AEJMV|1	1100.47959
gm-AEJMW|1	1187.49049
gm-AEJMY|1	1164.47451
gm-AEJN|1	984.41362
gm-AEJNA|1	1055.45076
gm-AEJNE|1	1113.45621
gm-AEJNG|1	1041.43508
gm-AEJNH|1	1121.47253
gm-AEJNN|1	1098.45655
gm-AEJNP|1	1081.46638
gm-AEJNQ|1	1112.47220
gm-AEJNT|1	1085.46130
gm-AEJNW|1	1170.49293
gm-AEJP|1	967.42345
gm-AEJPA|1	1038.46059
gm-AEJPE|1	1096.46604
gm-AEJPP|1	1064.47621
gm-AEJPQ|1	1095.48203

gm-AEJPT|1	1068.47113
gm-AEJPW|1	1153.50276
gm-AEJQ|1	998.42917
gm-AEJQA|1	1069.46641
gm-AEJQC|1	1101.43846
gm-AEJQE|1	1127.47186
gm-AEJQG|1	1055.45073
gm-AEJQQ|1	1126.48785
gm-AEJQS|1	1085.46130
gm-AEJQT|1	1099.47695
gm-AEJQW|1	1184.50858
gm-AEJR|1	1026.47180
gm-AEJRA|1	1097.50894
gm-AEJRC|1	1129.48099
gm-AEJRD|1	1141.49874
gm-AEJRE|1	1155.51439
gm-AEJRH|1	1163.53071
gm-AEJRI|1	1139.55586
gm-AEJRM|1	1157.51229
gm-AEJRN|1	1140.51473
gm-AEJRP|1	1123.52456
gm-AEJRQ|1	1154.53038
gm-AEJRR|1	1182.57291
gm-AEJRT|1	1127.51948
gm-AEJRV|1	1125.54021
gm-AEJRW|1	1212.55111
gm-AEJS|1	957.40272
gm-AEJSA|1	1028.43986
gm-AEJSD|1	1072.42966
gm-AEJSE|1	1086.44531
gm-AEJSK|1	1085.49768
gm-AEJSN|1	1071.44565
gm-AEJSP|1	1054.45548
gm-AEJSQ|1	1085.46130
gm-AEJSR|1	1113.50383
gm-AEJSS|1	1044.43475
gm-AEJST|1	1058.45040
gm-AEJSW|1	1143.48203
gm-AEJT|1	971.41837
gm-AEJTA|1	1042.45551
gm-AEJTC|1	1074.42756
gm-AEJTE|1	1100.46096
gm-AEJTG|1	1028.43983
gm-AEJTK|1	1099.51333
gm-AEJTT|1	1072.46605
gm-AEJTV|1	1070.48678
gm-AEJV|1	969.43910
gm-AEJVA|1	1040.47624
gm-AEJVC|1	1072.44829
gm-AEJVD|1	1084.46604
gm-AEJVE|1	1098.48169
gm-AEJVH|1	1106.49801
gm-AEJVK|1	1097.53406
gm-AEJVN|1	1083.48203
gm-AEJVP|1	1066.49186
gm-AEJVQ|1	1097.49768
gm-AEJVS|1	1056.47113
gm-AEJVT|1	1070.48678
gm-AEJVV|1	1068.50751
gm-AEJVW|1	1155.51841
gm-AEJW|1	1056.45000
gm-AEJWA|1	1127.48714
gm-AEJWC|1	1159.45919
gm-AEJWE|1	1185.49259
gm-AEJWT|1	1157.49768
gm-AEJWW|1	1242.52931
gm-AEJY|1	1033.43402
gm-AEJYA|1	1104.47116
gm-AEJYC|1	1136.44321
gm-AEJYD|1	1148.46096
gm-AEJYE|1	1162.47661

gm-AEJYG|1	1090.45548
gm-AEJYH|1	1170.49293
gm-AEJYI|1	1146.51808
gm-AEJYK|1	1161.52898
gm-AEJYN|1	1147.47695
gm-AEJYP|1	1130.48678
gm-AEJYQ|1	1161.49260
gm-AEJYR|1	1189.53513
gm-AEJYS|1	1120.46605
gm-AEJYT|1	1134.48170
gm-AEJYV|1	1132.50243
gm-AEJYW|1	1219.51333
gm-AEJYY|1	1196.49735

Table S4. *Rhizobium leguminosarum* unbiased search with DB_1

	Muropeptide [a]	TY1	TY2	TY3		RT (min)	Theoretica mass (Da)	Δpp	Prese t in
1	gm-AEJA|1	34.34	43.678	45.233	9.	0.	941.40	0.2	3
2	gm-AEJG|1	9.361	8.946	9.535	6.	0.	927.39	0.7	3
3	gm-AEJQE|1	8.833	6.551	4.017	1	0.	1127.47	1.2	3
4	gm-AEJ|1	6.344	5.207	7.248	5.	0.	870.37	0.5	3
5	gm-AEJIA|1	5.847	6.038	5.729	2	0.	1054.49	0.9	3
6	gm-AEJF|1	5.423	5.917	5.617	2	0.	1017.43	2.0	3
7	gm-AEJAA|1	1.862	2.891	2.772	1	0.	1012.44	1.2	3
8	gm-AEJFA|1	2.248	2.039	1.456	1	0.	1088.47	0.7	3
9	gm-AEJAG|1, gm-AEJQ|1	1.605	1.697	1.979	8.	0.	998.42	1.2	3
1	gm-AEJK|1	2.314	1.576	1.371	7.	0.	998.46	1.6	3
1	gm-AEJPA|1	3.017	1.059	0.695	2	0.	1038.46	9.1	3
1	gm-AEJY|1	1.251	1.432	1.202	1	0.	1033.43	1.4	3
1	gm-AEJWW|1	2.392	0.508	0.898	9.	0.	1242.52	6.2	3
1	gm-AEJVC|1, gm-AEJMA|	1.066	1.216	1.017	1	0.	1072.44	1.8	3
1	gm-AEJRC|1	1.004	1.288	0.984	2	0.	1129.48	7.5	3
1	gm-AEJGG|1, gm-AEJN|1	0.955	0.961	1.178	5.	0.	984.41	0.8	3
1	gm-AEJDA|1, gm-AEJEG|1	1.376	0.807	0.701	9.	3.	1056.43	1.4	3
1	gm-AEJM|1	0.790	0.897	0.648	1	0.	1001.41	0.5	3
1	gm-AEJI|1	0.745	0.722	0.611	2	0.	983.45	0.8	3
2	gm-AE|1	0.875	0.423	0.680	8.	0.	698.28	0.3	3
2	gm-AEJS|1	0.643	0.403	0.753	5.	5.	957.40	0.4	3
2	gm-AEJWA|1	0.541	0.641	0.510	2	0.	1127.48	1.3	3
2	gm-AEJH|1	0.427	0.343	0.334	5.	0.	1007.42	1.2	3
2	gm-AEJCC|1	0.619	0.235	0.150	2	0.	1076.38	8.9	3
2	gm-AEJR|1	0.292	0.270	0.272	6.	0.	1026.47	0.6	3
2	gm-AEJW|1	0.244	0.276	0.217	2	0.	1056.45	1.6	3
2	gm-AEJV|1	0.230	0.255	0.237	1	0.	969.43	0.9	3
2	gm-AEJQA|1	0.206	0.198	0.215	1	0.	1069.46	1.9	3
2	gm-AEJMY|1	0.403	0.131	0.054	2	0.	1164.47	5.1	3
3	gm-AEJHA|1	0.209	0.197	0.169	7.	0.	1078.46	1.9	3
3	gm-AEJFS|1, gm-AEJHP|1, gm-AEJYA|1	0.187	0.179	0.196	1	0.	1104.47	1.1	3
3	gm-AEJT|1	0.169	0.184	0.165	7.	8.	971.41	1.9	3
3	gm-AEJEA|1	0.143	0.147	0.226	1	2.	1070.45	1.9	3
3	gm-AEJKD|1, gm-AEJSR|1	0.235	0.130	0.147	9.	0.	1113.49	1.4	3
3	gm-AEJKP|1	0.166	0.168	0.148	3	0.	1095.51	4.3	3
3	gm-AEJIQ|1	0.064	0.165	0.239	2	0.	1111.51	4.2	3
3	gm-AEJST|1	0.219	0.065	0.086	2	0.	1058.45	2.7	3
3	gm-AEJMS|1	0.125	0.114	0.108	1	0.	1088.44	2.0	3
3	gm-AEJEV|1, gm-AEJID|1	0.109	0.103	0.095	1	0.	1098.48	0.7	3
4	gm-AEJRA|1	0.092	0.113	0.099	9.	6.	1097.50	1.9	3
4	gm-AEJKA|1	0.135	0.082	0.071	7.	0.	1069.50	2.2	3
4	gm-AEJVP|1	0.123	0.076	0.066	3	0.	1066.49	2.1	3
4	gm-AEJCG|1	0.119	0.096	0.050	1	1	1030.40	1.3	3
4	gm-AEJD|1	0.104	0.030	0.117	7.	0.	985.39	0.3	3
4	gm-AEJTA|1	0.095	0.098	0.046	2	0.	1042.45	2.7	3
4	gm-AEJTC|1	0.082	0.050	0.059	2	0.	1074.42	3.2	3
4	gm-AEJKQ|1	0.058	0.059	0.051	7.	0.	1126.52	1.4	3
4	gm-AEJEI|1, gm-AEJKN|1	0.050	0.058	0.059	2	1.	1112.49	2.9	3
4	gm-AEJMC|1	0.078	0.036	0.051	1	0.	1104.42	5.5	3
5	gm-AEJR|1, gm-AEJGV|1	0.044	0.055	0.064	3	2.	1026.47	1.3	3
5	gm-AEJCH|1	0.080	0.051	0.026	2	0.	1110.43	5.6	3
5	gm-AEJIN|1, gm-AEJVQ|1 gm-AEJRA|1	0.046	0.050	0.061	1	7.	1097.49	1.1	3
5	gm-AEJKG|1	0.049	0.069	0.038	1	0.	1055.48	4.0	3
5	gm-AEJCK|1	0.062	0.043	0.041	2	8.	1101.47	5.9	3
5	gm-AEJDT|1, gm-AEJES|1	0.072	0.034	0.036	2	0.	1086.44	2.6	3
5	gm-AEJHT|1	0.049	0.054	0.034	1	0.	1108.47	2.1	3
5	gm-AEJTE|1	0.093	0.013	0.026	1	0.	1100.46	7.6	3
5	gm-AEJNA|1, gm-AEJGQ|	0.044	0.046	0.044	7.	1.	1055.45	1.3	3
5	gm-AEJFR|1	0.052	0.036	0.042	1	0.	1173.54	1.4	3
6	gm-AEJYT|1	0.075	0.029	0.026	8.	0.	1134.48	1.5	3
6	gm-AEJP|1	0.089	0.016	0.021	2	0.	967.42	8.0	3
6	gm-AEJRH|1	0.050	0.031	0.029	2	0.	1163.53	6.8	3
6	gm-AEJYC|1	0.040	0.040	0.025	1	0.	1136.44	1.5	3
6	gm-AEJSA|1, gm-AEJTG|1	0.025	0.030	0.044	8.	0.	1028.43	0.8	3
6	gm-AEJYI|1	0.068	0.012	0.015	2	0.	1146.51	4.5	3
6	gm-AEJIP|1	0.033	0.021	0.039	1	0.	1080.50	2.6	3
6	gm-AEJQT|1	0.031	0.024	0.026	4.	0.	1099.47	1.0	3
6	gm-AEJRV|1	0.035	0.023	0.021	2	3.	1125.54	7.4	3
6	gm-AEJIH|1	0.045	0.013	0.015	1	0.	1120.51	2.7	3
7	gm-AEJDN|1	0.026	0.025	0.021	1	0.	1099.44	3.0	3
7	gm-AEJFD|1	0.017	0.026	0.028	2	0.	1132.46	2.9	3

**7**	gm-AEJHP|1	0.031	0.018	0.013	2	0.	1104.48	8.8	3
**7**	gm-AEJFE|1	0.037	0.008	0.014	2	0.	1146.48	5.1	3
**7**	gm-AEJYS|1	0.020	0.023	0.014	1	0.	1120.46	9.1	3
**7**	gm-AEJFY|1	0.023	0.018	0.013	1	0.	1180.50	2.3	3
**7**	gm-AEJRD|1	0.022	0.018	0.009	2	0.	1141.49	4.9	3
**7**	gm-AEJVS|1	0.031	0.009	0.010	1	0.	1056.47	2.1	3
**7**	gm-AEJPW|1	0.019	0.011	0.016	8.	0.	1153.50	6.0	3
**7**	gm-AEJSR|1	ND	0.009	0.230	2	0.	1113.50	7.9	2
**8**	gm-AEJMD|1	0.075	0.058	ND	1	8.	1116.43	0.4	2
**8**	gm-AEJFP|1	ND	0.039	0.050	3	0.	1114.49	8.3	2
**8**	gm-AEJYP|1	0.053	ND	0.022	2	0.	1130.48	2.1	2
**8**	gm-AEJNH|1	0.041	ND	0.030	2	0.	1121.47	1.5	2
**8**	gm-AEJIW|1	0.051	ND	0.012	2	0.	1169.53	8.0	2
**8**	gm-AEJE|1	0.016	0.047	ND	1	0.	999.41	2.0	2
**8**	gm-AEJLC|1	0.043	ND	0.017	2	0.	1086.46	8.0	2
**8**	gm-AEJVN|1, gm-AEJGR|	0.034	0.026	ND	2	0.	1083.48	3.0	2
**8**	gm-AEJGR|1	0.042	0.017	ND	2	0.	1083.49	4.9	2
**8**	gm-AEJKD|1	ND	0.043	0.015	2	0.	1113.49	6.5	2
**9**	gm-AEJRW|1	ND	0.015	0.032	2	0.	1212.55	9.9	2
**9**	gm-AEJYD|1	0.031	ND	0.009	2	0.	1148.46	3.6	2
**9**	gm-AEJKE|1, gm-AEJRT|1	ND	0.023	0.015	1	0.	1127.50	3.6	2
**9**	gm-AEJRI|1	0.022	ND	0.009	2	0.	1139.55	8.4	2
**9**	gm-AEJED|1	ND	0.008	0.023	8.	0.	1114.44	0.2	2
**9**	gm-AEJKH|1	0.014	0.013	ND	1	0.	1135.52	2.3	2
**9**	gm-AEJFS|1, gm-AEJYA|1	ND	0.012	0.010	2	0.	1104.47	3.2	2
**9**	gm-AEJGH|1	ND	0.012	0.010	2	0.	1064.45	5.5	2
**9**	gm-AEJMT|1	0.010	ND	0.009	2	0.	1102.45	5.5	2
**9**	gm-AEJRN|1	ND	0.010	0.009	2	0.	1140.51	4.7	2
**1**	gm-AEJMF|1, gm-AEJYD|	ND	0.005	0.006	9.	0.	1148.47	6.9	2
**1**	gm-AEJNP|1	0.064	ND	ND	3	9.	1081.46	8.8	1
**1**	gm-AEJSD|1	0.054	ND	ND	2	0.	1072.42	7.1	1
**1**	gm-AEJFH|1	0.051	ND	ND	1	0.	1154.49	7.6	1
**1**	gm-AEJMP|1	0.041	ND	ND	1	0.	1098.46	3.8	1
**1**	gm-AEJFP|1, gm-AEJMI|1	0.032	ND	ND	2	0.	1114.49	1.7	1
**1**	gm-AEJQQ|1	0.031	ND	ND	3	0.	1126.48	0.5	1
**1**	gm-AEJGS|1	0.030	ND	ND	2	9.	1014.42	9.8	1
**1**	gm-AEJRM|1	0.027	ND	ND	2	0.	1157.51	7.5	1
**1**	gm-AEJYN|1	0.024	ND	ND	1	0.	1147.47	5.0	1
**1**	gm-AEJDG|1	ND	ND	0.023	7.	0.	1042.41	9.3	1
**1**	gm-AEJYQ|1	ND	ND	0.022	1	0.	1161.49	1.1	1
**1**	gm-AEJNN|1	0.022	ND	ND	1	1	1098.45	8.5	1
**1**	gm-AEJMV|1	ND	0.021	ND	3	0.	1100.47	8.3	1
**1**	gm-AEJEH|1	ND	ND	0.019	2	0.	1136.47	5.6	1
**1**	gm-AEJFT|1	0.016	ND	ND	2	0.	1118.48	1.8	1
**1**	gm-AEJGP|1	ND	0.013	ND	2	0.	1024.44	9.1	1
**1**	gm-AEJWE|1	0.011	ND	ND	2	0.	1185.49	3.6	1
**1**	gm-AEJKR|1	ND	0.011	ND	2	0.	1154.56	9.5	1
**1**	gm-AEJHH|1	ND	ND	0.010	1	0.	1144.48	0.2	1
**1**	gm-AEJGM|1	ND	ND	0.010	1	0.	1058.43	4.5	1
**1**	gm-AEJRA|1, gm-AEJIN|1, gm-AEJVQ|1	ND	ND	0.010	2	0.	1097.50	0.2	1
**1**	gm-AEJST|1, gm-AEJGM|	ND	0.010	ND	1	0.	1058.45	7.3	1
**1**	gm-AEJMF|1	0.010	ND	ND	9.	0.	1148.47	5.9	1
**1**	gm-AEJMN|1	0.010	ND	ND	2	0.	1115.45	9.7	1
**1**	gm-AEJHQ|1	ND	ND	0.010	2	0.	1135.48	5.0	1
**1**	gm-AEJSN|1	0.009	ND	ND	2	0.	1071.44	9.6	1
**1**	gm-AEJKT|1	0.009	ND	ND	1	0.	1099.51	3.5	1
**1**	gm-AEJMP|1, gm-AEJNN|	ND	ND	0.008	8.	0.	1098.46	0.9	1
**1**	gm-AEJKN|1	ND	ND	0.007	2	0.	1112.50	8.9	1
**1**	gm-AEJKW|1	ND	0.007	ND	1	0.	1184.54	6.5	1
**1**	gm-AEJRT|1	ND	0.005	ND	4.	0.	1127.5195	4.9	1

[a] g, GlcNAc; m, MurNAc

[b] ND, Not Detected

Table S5. Database 2 (DB_2)

Structure	Monoisotopicmass
gm-AE|1	698.28580
gm-AEJ|1	870.37059
gm-AEJA|1	941.40770
gm-AEJAA|1	1012.44482
gm-AEJAD|1	1056.43465
gm-AEJAE|1	1070.45030
gm-AEJAF|1	1088.47612
gm-AEJAG|1	998.42917
gm-AEJAH|1	1078.46661
gm-AEJAI|1	1054.49177
gm-AEJAK|1	1069.50267
gm-AEJAM|1	1072.44819
gm-AEJAQ|1	1069.46628
gm-AEJAR|1	1097.50881
gm-AEJAW|1	1127.48702
gm-AEJD|1	985.39753
gm-AEJF|1	1017.43900
gm-AEJG|1	927.39205
gm-AEJH|1	1007.42950
gm-AEJI|1	983.45465
gm-AEJK|1	998.46555
gm-AEJM|1	1001.41107
gm-AEJN|1	984.41352
gm-AEJQ|1	998.42917
gm-AEJS|1	957.40262
gm-AEJT|1	971.41827
gm-AEJV|1	969.43900
gm-AEJW|1	1056.44990
gm-AEJY|1	1033.43392

Table S6. Cumulated abundance of monomers validated by MS/MS

	Structure	Average		Cumulated abundance
		Intensity	Abundance	
**1**	gm-AEJA|1	1.07E+09	47.90%	47.90%
**2**	gm-AEJG|1	2.43E+08	10.84%	58.74%
**3**	gm-AEJ|1	1.64E+08	7.33%	66.07%
**4**	gm-AEJA[I/L]|1	1.54E+08	6.85%	72.92%
**5**	gm-AEJF|1	1.48E+08	6.59%	79.51%
**6**	gm-AEJAA|1	6.55E+07	2.92%	82.43%
**7**	gm-AEJAF|1	5.00E+07	2.23%	84.67%
**8**	gm-AEJAG|1,gm-AEJQ|1	4.61E+07	2.06%	86.72%
**9**	gm-AEJK|1	4.60E+07	2.05%	88.77%
**10**	gm-AEJY|1	3.38E+07	1.51%	90.28%
**11**	gm-AEJAM|1	2.87E+07	1.28%	91.56%
**12**	gm-AEJN|1	2.70E+07	1.20%	92.77%
**13**	gm-AEJAD|1	2.52E+07	1.13%	93.89%
**14**	gm-AEJM|1	2.03E+07	0.91%	94.80%
**15**	gm-AEJ[I/L]|1	1.81E+07	0.81%	95.61%
**16**	gm-AE|1	1.73E+07	0.77%	96.38%
**17**	gm-AEJS|1	1.58E+07	0.70%	97.09%
**18**	gm-AEJAW|1	1.47E+07	0.66%	97.74%
**19**	gm-AEJH|1	9.64E+06	0.43%	98.17%
**20**	gm-AEJW|1	6.41E+06	0.29%	98.46%
**21**	gm-AEJV|1	6.29E+06	0.28%	98.74%
**22**	gm-AEJAQ|1	5.40E+06	0.24%	98.98%
**23**	gm-AEJAH|1	5.02E+06	0.22%	99.20%
**24**	gm-AEJT|1	4.51E+06	0.20%	99.40%
**25**	gm-AEJAE|1	4.51E+06	0.20%	99.61%
**26**	gm-AEJAR|1	4.11E+06	0.18%	99.79%
**27**	gm-AEJAK|1	2.53E+06	0.11%	99.90%
**28**	gm-AEJD|1	2.21E+06	0.10%	100.00%
	**Total**	2.24E+09		

Table S7. Database 3 (DB_3)

Structure	Monoisotopicmass
gm-AEJ|1	870.37059
gm-AEJA|1	941.40770
gm-AEJAA|1	1012.44482
gm-AEJAF|1	1088.47612
gm-AEJAI|1	1054.49177
gm-AEJF|1	1017.43900
gm-AEJG|1	927.39205
gm-AEJK|1	998.46555
gm-AEJAG|1, gm-AEJQ|1	998.42917
gm-AEJY|1	1033.43392

Table S8. Database 4 (DB_4)

Structure	Monoisotopicmass
gm-AEJADL|1	1169.51871
gm-AEJADLG|1	1226.54017
gm-AEJADLGT|1	1327.58785
gm-AEJADLGTR|1	1483.68896
gm-AEJADLGTRT|1	1584.73664
gm-AEJADLGTRTY|1	1747.79997
gm-AEJADLGTRTYE|1	1876.84256
gm-AEJADLGTRTYEE|1	2005.88516
gm-AEJADLGTRTYEEP|1	2102.93792
gm-AEJADLGTRTYEEPD|1	2217.96486
gm-AEJADLGTRTYEEPDL|1	2331.04893
gm-AEJADLGTRTYEEPDLR|1	2487.15004
gm-AEJADLGTRTYEEPDLRN|1	2601.19296
gm-AEJADLGTRTYEEPDLRNG|1	2658.21443
gm-AEJADLGTRTYEEPDLRNGV|1	2757.28284
gm-AEJADLGTRTYEEPDLRNGVK|1	2885.37781
gm-AEJED|1	1114.44012
gm-AEJEDL|1	1227.52419
gm-AEJEDLQ|1	1355.58277
gm-AEJEDLQF|1	1502.65118
gm-AEJEDLQFS|1	1589.68321
gm-AEJEDLQFSI|1	1702.76727
gm-AEJEDLQFSIY|1	1865.83060
gm-AEJEDLQFSIYG|1	1922.85206
gm-AEJEDLQFSIYGG|1	1979.87353
gm-AEJEDLQFSIYGGY|1	2142.93686
gm-AEJEDLQFSIYGGYQ|1	2270.99543
gm-AEJEDLQFSIYGGYQT|1	2372.04311
gm-AEJEDLQFSIYGGYQTA|1	2443.08023
gm-AEJEDLQFSIYGGYQTAP|1	2540.13299
gm-AEJEDLQFSIYGGYQTAPH|1	2677.19190
gm-AEJEDLQFSIYGGYQTAPHS|1	2764.22393
gm-AEJEDLQFSIYGGYQTAPHSG|1	2821.24539
gm-AEJAV|1	1040.47612
gm-AEJAVR|1	1196.57723
gm-AEJAVRD|1	1311.60417
gm-AEJAVRDV|1	1410.67258
gm-AEJAVRDVA|1	1481.70970
gm-AEJAVRDVAT|1	1582.75738
gm-AEJAVRDVATQ|1	1710.81595
gm-AEJAVRDVATQA|1	1781.85307
gm-AEJAVRDVATQAS|1	1868.88510
gm-AEJAVRDVATQASA|1	1939.92221
gm-AEJAVRDVATQASAV|1	2038.99062
gm-AEJAVRDVATQASAVQ|1	2167.04920
gm-AEJAVRDVATQASAVQQ|1	2295.10778
gm-AEJAVRDVATQASAVQQA|1	2366.14489
gm-AEJAVRDVATQASAVQQAE|1	2495.18749
gm-AEJAVRDVATQASAVQQAEQ|1	2623.24606
gm-AEJAVRDVATQASAVQQAEQG|1	2680.26753
gm-AEJDP|1	1082.45030
gm-AEJDPV|1	1181.51871
gm-AEJDPVP|1	1278.57147
gm-AEJDPVPR|1	1434.67258
gm-AEJDPVPRA|1	1505.70970
gm-AEJDPVPRAT|1	1606.75738
gm-AEJDPVPRATP|1	1703.81014
gm-AEJDPVPRATPV|1	1802.87855
gm-AEJDPVPRATPVA|1	1873.91567
gm-AEJDPVPRATPVAG|1	1930.93713
gm-AEJDPVPRATPVAGS|1	2017.96916
gm-AEJDPVPRATPVAGSV|1	2117.03757
gm-AEJDPVPRATPVAGSVI|1	2230.12164
gm-AEJDPVPRATPVAGSVIA|1	2301.15875
gm-AEJDPVPRATPVAGSVIAR|1	2457.25986
gm-AEJDPVPRATPVAGSVIARK|1	2585.35483
gm-AEJDPVPRATPVAGSVIARKI|1	2698.43889
gm-AEJCA|1	1044.41689
gm-AEJCAK|1	1172.51185
gm-AEJCAKK|1	1300.60681
gm-AEJCAKKN|1	1414.64974
gm-AEJCAKKNV|1	1513.71815
gm-AEJCAKKNVP|1	1610.77092
gm-AEJCAKKNVPN|1	1724.81385
gm-AEJCAKKNVPNS|1	1811.84587
gm-AEJCAKKNVPNSA|1	1882.88299
gm-AEJCAKKNVPNSAG|1	1939.90445
gm-AEJCAKKNVPNSAGD|1	2054.93139

gm-AEJCAKKNVPNSAGDL|1	2168.01546
gm-AEJCAKKNVPNSAGDLG|1	2225.03692
gm-AEJCAKKNVPNSAGDLGL|1	2338.12099
gm-AEJCAKKNVPNSAGDLGLG|1	2395.14245
gm-AEJCAKKNVPNSAGDLGLGA|1	2466.17956
gm-AEJCAKKNVPNSAGDLGLGAG|1	2523.20103
gm-AEJEN|1	1113.45611
gm-AEJENY|1	1276.51944
gm-AEJENYV|1	1375.58785
gm-AEJENYVT|1	1476.63553
gm-AEJENYVTL|1	1589.71959
gm-AEJENYVTLG|1	1646.74106
gm-AEJENYVTLGR|1	1802.84217
gm-AEJENYVTLGRL|1	1915.92623
gm-AEJENYVTLGRLV|1	2014.99465
gm-AEJENYVTLGRLVC|1	2118.00383
gm-AEJENYVTLGRLVCG|1	2175.02530
gm-AEJENYVTLGRLVCGS|1	2262.05732
gm-AEJENYVTLGRLVCGSD|1	2377.08427
gm-AEJENYVTLGRLVCGSDG|1	2434.10573
gm-AEJENYVTLGRLVCGSDGG|1	2491.12719
gm-AEJENYVTLGRLVCGSDGGQ|1	2619.18577
gm-AEJENYVTLGRLVCGSDGGQG|1	2676.20724
gm-AEJADA|1	1127.47176
gm-AEJADAI|1	1240.55582
gm-AEJADAIV|1	1339.62424
gm-AEJADAIVA|1	1410.66135
gm-AEJADAIVAA|1	1481.69846
gm-AEJADAIVAAE|1	1610.74106
gm-AEJADAIVAAEP|1	1707.79382
gm-AEJADAIVAAEPE|1	1836.83641
gm-AEJADAIVAAEPEP|1	1933.88918
gm-AEJADAIVAAEPEPV|1	2032.95759
gm-AEJADAIVAAEPEPVE|1	2162.00019
gm-AEJADAIVAAEPEPVEY|1	2325.06351
gm-AEJADAIVAAEPEPVEYV|1	2424.13193
gm-AEJADAIVAAEPEPVEYVR|1	2580.23304
gm-AEJADAIVAAEPEPVEYVRV|1	2679.30145
gm-AEJADAIVAAEPEPVEYVRVC|1	2782.31064
gm-AEJADAV|1	1226.54017
gm-AEJADAVD|1	1341.56712
gm-AEJADAVDQ|1	1469.62569
gm-AEJADAVDQV|1	1568.69411
gm-AEJADAVDQVP|1	1665.74687
gm-AEJADAVDQVPE|1	1794.78946
gm-AEJADAVDQVPEA|1	1865.82658
gm-AEJADAVDQVPEAP|1	1962.87934
gm-AEJADAVDQVPEAPV|1	2061.94776
gm-AEJADAVDQVPEAPVA|1	2132.98487
gm-AEJADAVDQVPEAPVAQ|1	2261.04345
gm-AEJADAVDQVPEAPVAQE|1	2390.08604
gm-AEJADAVDQVPEAPVAQEA|1	2461.12315
gm-AEJADAVDQVPEAPVAQEAP|1	2558.17592
gm-AEJADAVDQVPEAPVAQEAPV|1	2657.24433

Table S9. PGFinder identification of dimers and trimers

	Structure	Theo mass (Da)	Abundance (%)	Cumul. %	RT (min)
**DIMERS**					
1	gm-AEJAA-gm-AEJ|2, gm-AEJA-gm-AEJA|2	1864.805	24.40%	24.40%	15.7 ± 0.0
2	gm-AEJ-gm-AEJA|2, gm-AEJA-gm-AEJ|2	1793.768	23.04%	47.44%	14.9 ± 0.0
3	gm-AEJG-gm-AEJ|2	1779.752	16.18%	63.62%	13.6 ± 0.0
4	gm-AEJ-gm-AEJ|2	1722.731	12.82%	76.44%	13.9 ± 0.0
5	gm-AEJG-gm-AEJA|2, gm-AEJQ|1, gm-AEJAG-gm-AEJ|2	1850.789	7.79%	84.23%	14.4 ± 0.0
6	gm-AEJF-gm-AEJ|2	1869.799	2.40%	86.63%	23.9 ± 0.1
7	gm-AEJN-gm-AEJ|2	1836.773	1.84%	88.47%	13.0 ± 0.0
8	gm-AEJ(AG/Q)-gm-AEJA|2	1921.826	1.57%	90.03%	15.0 ± 0.0
9	gm-AEJS-gm-AEJ|2	1809.763	1.18%	91.22%	13.3 ± 0.0
10	gm-AEJF-gm-AEJA|2, gm-AEJAF-gm-AEJ|2	1940.836	0.94%	92.16%	24.7 ± 0.0
11	gm-AEJAI-gm-AEJ|2, gm-AEJI-gm-AEJA|2	1906.852	0.92%	93.08%	23.4 ± 0.0
12	gm-AEJAI-gm-AEJA|2	1977.889	0.90%	93.98%	24.2 ± 0.0
13	gm-AEJN-gm-AEJA|2	1907.811	0.57%	94.55%	13.6 ± 0.0
14	gm-AEJS-gm-AEJA|2	1880.800	0.49%	95.04%	14.1 ± 0.0
15	gm-AEJY-gm-AEJ|2	1885.794	0.47%	95.51%	19.6 ± 0.0
16	gm-AEJAD-gm-AEJA|2, gm-AEJW-gm-AEJA|2, gm-AEJAW-gm-AEJ|2	1979.832	0.42%	95.93%	16.8 ± 0.0
17	gm-AEJK-gm-AEJ|2	1850.825	0.40%	96.34%	12.6 ± 0.0
18	gm-AEJM-gm-AEJ|2	1853.771	0.37%	96.71%	19.0 ± 0.0
19	gm-AEJAA-gm-AEJA|2	1935.842	0.35%	97.06%	16.3 ± 0.0
20	gm-AEJI-gm-AEJ|2	1835.815	0.34%	97.40%	22.8 ± 0.0
21	gm-AEJH-gm-AEJ|2	1859.789	0.33%	97.74%	12.6 ± 0.0
22	gm-AEJH-gm-AEJA|2, gm-AEJAH-gm-AEJ|2	1930.827	0.21%	97.95%	13.3 ± 0.0
23	gm-AEJT-gm-AEJ|2	1823.778	0.20%	98.15%	13.9 ± 0.0
24	gm-AEJM-gm-AEJA|2, gm-AEJAM-gm-AEJ|2	1924.808	0.19%	98.34%	19.7 ± 0.0
25	gm-AEJY-gm-AEJA|2	1956.831	0.18%	98.52%	20.4 ± 0.0
26	gm-AEJW-gm-AEJA|2, gm-AEJAW-gm-AEJ|2, gm-AEJAD-gm-AEJA|2	1979.847	0.16%	98.68%	26.5 ± 0.0
27	gm-AEJAM-gm-AEJA|2	1995.845	0.15%	98.83%	20.4 ± 0.0
28	gm-AEJAK-gm-AEJ|2, gm-AEJK-gm-AEJA|2	1921.863	0.14%	98.97%	13.2 ± 0.0
29	gm-AEJAD-gm-AEJ|2, gm-AEJD-gm-AEJA|2, gm-AEJW-gm-AEJ|2	1908.795	0.14%	99.11%	14.5 ± 0.0
30	gm-AEJAW-gm-AEJA|2	2050.884	0.14%	99.25%	27.0 ± 0.0
31	gm-AEJV-gm-AEJ|2	1821.799	0.11%	99.36%	19.1 ± 0.0
32	gm-AEJD-gm-AEJ|2	1837.757	0.10%	99.46%	13.7 ± 0.0
33	gm-AEJT-gm-AEJA|2	1894.815	0.10%	99.56%	14.7 ± 0.0
34	gm-AEJAR-gm-AEJ|2	1949.869	0.09%	99.65%	13.9 ± 0.1
35	gm-AEJAF-gm-AEJA|2	2011.873	0.08%	99.73%	25.9 ± 0.0
36	gm-AEJW-gm-AEJ|2, gm-AEJD-gm-AEJA|2, gm-AEJAD-gm-AEJ|2	1908.810	0.08%	99.80%	25.4 ± 0.0
37	gm-AEJAH-gm-AEJA|2	2001.864	0.06%	99.86%	13.9 ± 0.0
38	gm-AEJV-gm-AEJA|2	1892.836	0.05%	99.92%	19.9 ± 0.0
39	gm-AEJAE-gm-AEJA|2	1993.847	0.03%	99.95%	15.8 ± 0.0
40	gm-AEJAK-gm-AEJA|2	1992.900	0.03%	99.98%	13.8 ± 0.0

41	gm-AEJAQ-gm-AEJA|2	1992.863	0.02%	100.00%	16.4 ± 0.0
**TRIMERS**					
1	gm-AEJAA-gm-AEJ-gm-AEJA|3, gm-AEJA-gm-AEJA-gm-AEJA|3	2788.202	18.26%	18.26%	18.6 ± 0.0
2	gm-AEJAA-gm-AEJ-gm-AEJ|3, gm-AEJA-gm-AEJ-gm-AEJA|3, gm-AEJ-gm-AEJA-gm-AEJA|3	2717.165	18.09%	36.35%	18.1 ± 0.0
3	gm-AEJA-gm-AEJ-gm-AEJ|3, gm-AEJ-gm-AEJ-gm-AEJA|3	2646.127	15.90%	52.25%	17.6 ± 0.0
4	gm-AEJG-gm-AEJ-gm-AEJ|3	2632.112	12.08%	64.32%	16.5 ± 0.0
5	gm-AEJ-gm-AEJ-gm-AEJ|3	2575.090	9.34%	73.66%	16.7 ± 0.0
6	gm-AEJQ|1, gm-AEJAG-gm-AEJ-gm-AEJ|3, gm-AEJG-gm-AEJ-gm-AEJA|3	2703.149	7.47%	81.13%	17.0 ± 0.0
7	gm-AEJAQ-gm-AEJ-gm-AEJ|3, gm-AEJQ|1, gm-AEJAG-gm-AEJ-gm-AEJA|3, gm-AEJG-gm-AEJA-gm-AEJA|3	2774.186	5.39%	86.52%	17.5 ± 0.0
8	gm-AEJF-gm-AEJ-gm-AEJ|3	2722.159	2.02%	88.54%	25.0 ± 0.0
9	gm-AEJAQ-gm-AEJ-gm-AEJA|3, gm-AEJQ|1, gm-AEJAG-gm-AEJA-gm-AEJA|3	2845.223	1.34%	89.88%	18.0 ± 0.0
10	gm-AEJN-gm-AEJ-gm-AEJ|3	2689.133	1.29%	91.17%	15.8 ± 0.0
11	gm-AEJS-gm-AEJ-gm-AEJ|3	2662.122	0.90%	92.08%	16.2 ± 0.0
12	gm-AEJAF-gm-AEJ-gm-AEJ|3, gm-AEJF-gm-AEJ-gm-AEJA|3	2793.196	0.77%	92.85%	25.6 ± 0.0
13	gm-AEJN-gm-AEJ-gm-AEJA|3	2760.170	0.72%	93.57%	16.3 ± 0.0
14	gm-AEJN-gm-AEJA-gm-AEJA|3	2831.207	0.53%	94.10%	16.9 ± 0.0
15	gm-AEJAF-gm-AEJ-gm-AEJA|3, gm-AEJF-gm-AEJA-gm-AEJA|3	2864.233	0.50%	94.60%	26.0 ± 0.0
16	gm-AEJS-gm-AEJ-gm-AEJA|3	2733.159	0.44%	95.04%	16.7 ± 0.0
17	gm-AEJAI-gm-AEJ-gm-AEJ|3, gm-AEJI-gm-AEJ-gm-AEJA|3	2759.212	0.40%	95.43%	24.6 ± 0.0
18	gm-AEJH-gm-AEJ-gm-AEJ|3	2712.149	0.35%	95.79%	17.0 ± 1.1
19	gm-AEJAI-gm-AEJA-gm-AEJA|3	2901.286	0.35%	96.14%	25.6 ± 0.0
20	gm-AEJS-gm-AEJA-gm-AEJA|3	2804.197	0.35%	96.49%	17.2 ± 0.0
21	gm-AEJAI-gm-AEJ-gm-AEJA|3, gm-AEJI-gm-AEJA-gm-AEJA|3	2830.249	0.32%	96.81%	25.0 ± 0.0
22	gm-AEJY-gm-AEJ-gm-AEJ|3	2738.154	0.30%	97.12%	21.3 ± 0.0
23	gm-AEJM-gm-AEJ-gm-AEJ|3	2706.131	0.28%	97.40%	21.4 ± 0.7
24	gm-AEJK-gm-AEJ-gm-AEJ|3	2703.185	0.24%	97.64%	15.4 ± 0.0
25	gm-AEJAA-gm-AEJA-gm-AEJA|3	2859.239	0.24%	97.88%	19.0 ± 0.0
26	gm-AEJI-gm-AEJ-gm-AEJ|3	2688.174	0.22%	98.10%	24.0 ± 0.0
27	gm-AEJAD-gm-AEJ-gm-AEJA|3, gm-AEJAW-gm-AEJ-gm-AEJ|3, gm-AEJW-gm-AEJ-gm-AEJA|3, gm-AEJD-gm-AEJA-gm-AEJA|3	2832.192	0.20%	98.30%	18.9 ± 0.0
28	gm-AEJAM-gm-AEJ-gm-AEJ|3, gm-AEJM-gm-AEJ-gm-AEJA|3	2777.168	0.15%	98.45%	21.5 ± 0.0
29	gm-AEJT-gm-AEJ-gm-AEJ|3	2676.138	0.15%	98.60%	16.7 ± 0.0
30	gm-AEJAH-gm-AEJ-gm-AEJ|3, gm-AEJH-gm-AEJ-gm-AEJA|3	2783.186	0.13%	98.73%	16.0 ± 0.0
31	gm-AEJAM-gm-AEJ-gm-AEJA|3, gm-AEJM-gm-AEJA-gm-AEJA|3	2848.205	0.12%	98.85%	22.0 ± 0.0
32	gm-AEJAW-gm-AEJ-gm-AEJ|3, gm-AEJW-gm-AEJ-gm-AEJA|3, gm-AEJAD-gm-AEJ-gm-AEJA|3, gm-AEJD-gm-AEJA-gm-AEJA|3	2832.207	0.12%	98.97%	27.1 ± 0.0
33	gm-AEJY-gm-AEJ-gm-AEJA|3	2809.191	0.12%	99.09%	21.8 ± 0.0
34	gm-AEJY-gm-AEJA-gm-AEJA|3	2880.228	0.12%	99.21%	22.5 ± 0.0
35	gm-AEJAW-gm-AEJA-gm-AEJA|3	2974.281	0.10%	99.31%	24.5 ± 4.8
36	gm-AEJAH-gm-AEJ-gm-AEJA|3, gm-AEJH-gm-AEJA-gm-AEJA|3	2854.223	0.10%	99.41%	16.5 ± 0.0
37	gm-AEJV-gm-AEJ-gm-AEJ|3	2674.159	0.08%	99.49%	21.4 ± 0.0
38	gm-AEJAM-gm-AEJA-gm-AEJA|3	2919.242	0.07%	99.56%	22.5 ± 0.0
39	gm-AEJAK-gm-AEJ-gm-AEJ|3, gm-AEJK-gm-AEJ-gm-AEJA|3	2774.222	0.07%	99.63%	15.9 ± 0.0
40	gm-AEJD-gm-AEJ-gm-AEJ|3	2690.117	0.06%	99.70%	17.2 ± 0.0
41	gm-AEJW-gm-AEJ-gm-AEJ|3, gm-AEJAD-gm-AEJ-gm-AEJ|3, gm-AEJD-gm-AEJ-gm-AEJA|3	2761.170	0.06%	99.76%	26.1 ± 0.0
42	gm-AEJAW-gm-AEJ-gm-AEJA|3, gm-AEJW-gm-AEJA-gm-AEJA|3, gm-AEJAD-gm-AEJA-gm-AEJA|3	2903.244	0.06%	99.82%	27.4 ± 0.0
43	gm-AEJT-gm-AEJ-gm-AEJA|3	2747.175	0.05%	99.87%	17.2 ± 0.0
44	gm-AEJAD-gm-AEJA-gm-AEJA|3, gm-AEJAW-gm-AEJ-gm-AEJA|3, gm-AEJW-gm-AEJA-gm-AEJA|3	2903.229	0.04%	99.91%	19.3 ± 0.0

45	gm-AEJV-gm-AEJ-gm-AEJA|3	2745.196	0.03%	99.95%	21.7 ±	0.0
46	gm-AEJV-gm-AEJA-gm-AEJA|3	2816.233	0.03%	99.98%	22.1 ±	0.0
47	gm-AEJAD-gm-AEJ-gm-AEJ|3, gm-AEJW-gm-AEJ-gm-AEJ|3, gm-AEJD-gm-AEJ-gm-AEJA|3	2761.154	0.02%	100.00%	17.8 ±	0.0

Table S10. *In silico* fragmentation of the two dimers (gm-AEJ=gm-AEJA (3-4) and gm-AEJA=gm-AEJ (3-3)) and consolidated list of expected ions

Ion Nb	Type	*m/z*	Parts	Ion Nb	Type	*m/z*	Parts
gm-AEJA=gm-AEJ (3-4) fragments				gm-AEJ=gm-AEJA (3-3) fragments			
**1**	C-Terminal	871.3779	gm(r)-AEJ	**1**	C-Terminal	90.0550	A*
**2**	C-Terminal	942.4150	A=gm(r)-AEJ (4-3)	**2**	C-Terminal	942.4150	gm(r)-AEJA
**3**	C-Terminal	1114.4998	gm(r)-AEJA=J (4-3)	**3**	C-Terminal	1114.4998	gm(r)-AEJ=JA (3-3)
**4**	C-Terminal	1114.4998	JA=gm(r)-AEJ (4-3)	**4**	C-Terminal	1114.4998	J=gm(r)-AEJA (3-3)
**5**	C-Terminal	1243.5424	gm(r)-AEJA=EJ (4-3)	**5**	C-Terminal	1243.5424	gm(r)-AEJ=EJA (3-3)
**6**	C-Terminal	1243.5424	EJA=gm(r)-AEJ (4-3)	**6**	C-Terminal	1243.5424	EJ=gm(r)-AEJA (3-3)
**7**	C-Terminal	1314.5795	gm(r)-AEJA=AEJ (4-3)	**7**	C-Terminal	1314.5795	gm(r)-AEJ=AEJA (3-3)
**8**	C-Terminal	1314.5795	AEJA=gm(r)-AEJ (4-3)	**8**	C-Terminal	1314.5795	AEJ=gm(r)-AEJA (3-3)
**9**	C-Terminal	1591.6956	m(r)-AEJA=gm(r)-AEJ (4-3)	**9**	C-Terminal	1591.6956	m(r)-AEJ=gm(r)-AEJA (3-3)
**10**	C-Terminal	1591.6956	gm(r)-AEJA=m(r)-AEJ (4-3)	**10**	C-Terminal	1591.6956	gm(r)-AEJ=m(r)-AEJA (3-3)
**11**	Internal	72.0444	A	**11**	Internal	72.0444	A
**12**	Internal	130.0499	E	**12**	Internal	130.0499	E
**13**	Internal	173.0921	J	**13**	Internal	173.0921	J
**14**	Internal	191.1026	J*	**14**	Internal	201.0870	AE
**15**	Internal	201.0870	AE	**15**	Internal	262.1397	JA
**16**	Internal	244.1292	JA	**16**	Internal	278.1234	m(r)
**17**	Internal	262.1397	A=J (4-3)	**17**	Internal	302.1347	EJ
**18**	Internal	278.1234	m(r)	**18**	Internal	345.1769	J=J (3-3)
**19**	Internal	302.1347	EJ	**19**	Internal	349.1605	m(r)-A
**20**	Internal	320.1452	EJ	**20**	Internal	373.1718	AEJ
**21**	Internal	349.1605	m(r)-A	**21**	Internal	391.1823	EJA
**22**	Internal	373.1718	EJA	**22**	Internal	434.2245	J=JA (3-3)
**23**	Internal	373.1718	AEJ	**23**	Internal	462.2195	AEJA
**24**	Internal	391.1823	AEJ	**24**	Internal	474.2195	J=EJ (3-3)
**25**	Internal	391.1823	A=EJ (4-3)	**25**	Internal	474.2195	EJ=J (3-3)
**26**	Internal	434.2245	JA=J (4-3)	**26**	Internal	478.2031	m(r)-AE
**27**	Internal	444.2089	AEJA	**27**	Internal	545.2566	J=AEJ (3-3)
**28**	Internal	462.2195	A=AEJ (4-3)	**28**	Internal	545.2566	AEJ=J (3-3)
**29**	Internal	478.2031	m(r)-AE	**29**	Internal	563.2671	J=EJA (3-3)
**30**	Internal	563.2671	JA=EJ (4-3)	**30**	Internal	563.2671	EJ=JA (3-3)
**31**	Internal	563.2671	EJA=J (4-3)	**31**	Internal	603.2620	EJ=EJ (3-3)
**32**	Internal	634.3042	JA=AEJ (4-3)	**32**	Internal	634.3042	J=AEJA (3-3)
**33**	Internal	634.3042	AEJA=J (4-3)	**33**	Internal	634.3042	AEJ=JA (3-3)

**34**	Internal	650.2879	m(r)-AEJ
**35**	Internal	668.2985	m(r)-AEJ
**36**	Internal	692.3097	EJA=EJ (4-3)
**37**	Internal	721.3250	m(r)-AEJA
**38**	Internal	739.3356	A=m(r)-AEJ (4-3)
**39**	Internal	763.3468	EJA=AEJ (4-3)
**40**	Internal	763.3468	AEJA=EJ (4-3)
**41**	Internal	834.3840	AEJA=AEJ (4-3)
**42**	Internal	911.4204	m(r)-AEJA=J (4-3)
**43**	Internal	911.4204	JA=m(r)-AEJ (4-3)
**44**	Internal	1040.4630	m(r)-AEJA=EJ (4-3)
**45**	Internal	1040.4630	EJA=m(r)-AEJ (4-3)
**46**	Internal	1111.5001	m(r)-AEJA=AEJ (4-3)
**47**	Internal	1111.5001	AEJA=m(r)-AEJ (4-3)
**48**	Internal	1388.6163	m(r)-AEJA=m(r)-AEJ (4-3)
**49**	N-Terminal	204.0866	g
**50**	N-Terminal	481.2028	gm(r)
**51**	N-Terminal	552.2399	gm(r)-A
**52**	N-Terminal	681.2825	gm(r)-AE
**53**	N-Terminal	853.3673	gm(r)-AEJ
**54**	N-Terminal	924.4044	gm(r)-AEJA
**55**		1794.7750	gm(r)-AEJA=gm(r)-AEJ (4-3)

*C-terminal residue (+H_2O)

**34**	Internal	650.2879	m(r)-AEJ
**35**	Internal	674.2992	EJ=AEJ (3-3)
**36**	Internal	674.2992	AEJ=EJ (3-3)
**37**	Internal	692.3097	EJ=EJA (3-3)
**38**	Internal	739.3356	m(r)-AEJA
**39**	Internal	745.3363	AEJ=AEJ (3-3)
**40**	Internal	763.3468	EJ=AEJA (3-3)
**41**	Internal	763.3468	AEJ=EJA (3-3)
**42**	Internal	822.3727	m(r)-AEJ=J (3-3)
**43**	Internal	822.3727	J=m(r)-AEJ (3-3)
**44**	Internal	834.3840	AEJ=AEJA (3-3)
**45**	Internal	911.4204	m(r)-AEJ=JA (3-3)
**46**	Internal	911.4204	J=m(r)-AEJA (3-3)
**47**	Internal	951.4153	m(r)-AEJ=EJ (3-3)
**48**	Internal	951.4153	EJ=m(r)-AEJ (3-3)
**49**	Internal	1022.4524	m(r)-AEJ=AEJ (3-3)
**50**	Internal	1022.4524	AEJ=m(r)-AEJ (3-3)
**51**	Internal	1025.4521	gm(r)-AEJ=J (3-3)
**52**	Internal	1025.4521	J=gm(r)-AEJ (3-3)
**53**	Internal	1040.4630	m(r)-AEJ=EJA (3-3)
**54**	Internal	1040.4630	EJ=m(r)-AEJA (3-3)
**55**	Internal	1111.5001	m(r)-AEJ=AEJA (3-3)
**56**	Internal	1111.5001	AEJ=m(r)-AEJA (3-3)
**57**	Internal	1154.4947	gm(r)-AEJ=EJ (3-3)
**58**	Internal	1154.4947	EJ=gm(r)-AEJ (3-3)
**59**	Internal	1225.5318	gm(r)-AEJ=AEJ (3-3)
**60**	Internal	1225.5318	AEJ=gm(r)-AEJ (3-3)
**61**	Internal	1299.5686	m(r)-AEJ=m(r)-AEJ (3-3)
**62**	Internal	1388.6163	m(r)-AEJ=m(r)-AEJA (3-3)
**63**	Internal	1502.6480	m(r)-AEJ=gm(r)-AEJ (3-3)
**64**	Internal	1502.6480	gm(r)-AEJ=m(r)-AEJ (3-3)
**65**	N-Terminal	204.0866	g
**66**	N-Terminal	481.2028	gm(r)
**67**	N-Terminal	552.2399	gm(r)-A
**68**	N-Terminal	681.2825	gm(r)-AE
**69**	N-Terminal	853.3673	gm(r)-AEJ
**70**	N-Terminal	1705.7273	gm(r)-AEJ=gm(r)-AEJ (3-3)
**71**		1794.7750	gm(r)-AEJ=gm(r)-AEJA (3-3)

*C-terminal residue (+H_2O)

Ion Nb	*m/z*	Parts	
		gm-AEJA=gm-AEJ (4-3)	gm-AEJ=gm-AEJA (3-3)
1	72.04439	A	A
2	90.054954	N/A*	A**
3	130.04987	E	E
4	173.09207	J	J
5	191.10263	J*	N/A
6	201.08698	AE	AE
7	204.08665	g	g
8	244.12918	JA	N/A
9	262.13975	A=J (4-3)	JA
10	278.12343	m(r)	m(r)
11	302.13466	EJ	EJ
12	320.14523	EJ	N/A
13	345.17686	N/A	J=J (3-3)
14	349.16054	m(r)-A	m(r)-A
15	373.17178	EJA	AEJ
16	391.18234	AEJ	EJA
17	434.22454	JA=J (4-3)	J=JA (3-3)
18	444.20889	AEJA	N/A
19	462.21945	A=AEJ (4-3)	AEJA
20	474.21945	N/A	J=EJ (3-3)
21	478.20314	m(r)-AE	m(r)-AE
22	481.2028	gm(r)	gm(r)
23	545.25657	N/A	J=AEJ (3-3)
24	552.23991	gm(r)-A	gm(r)-A
25	563.26713	JA=EJ (4-3)	J=EJA (3-3)
26	603.26205	N/A	EJ=EJ (3-3)
27	634.30425	JA=AEJ (4-3)	J=AEJA (3-3)
28	650.28793	m(r)-AEJ	m(r)-AEJ
29	668.29849	m(r)-AEJ	N/A
30	674.29916	N/A	EJ=AEJ (3-3)

**31**	681.28251	gm(r)-AE	gm(r)-AE
**32**	692.30973	EJA=EJ (4-3)	EJ=EJA (3-3)
**33**	721.32504	m(r)-AEJA	N/A
**34**	739.33561	A=m(r)-AEJ (4-3)	m(r)-AEJA
**35**	745.33627	N/A	AEJ=AEJ (3-3)
**36**	763.34684	EJA=AEJ (4-3)	EJ=AEJA (3-3)
**37**	822.37272	N/A	m(r)-AEJ=J (3-3)
**38**	834.38395	AEJA=AEJ (4-3)	AEJ=AEJA (3-3)
**39**	853.3673	gm(r)-AEJ	gm(r)-AEJ
**40**	871.37786	gm(r)-AEJ	N/A
**41**	911.4204	m(r)-AEJA=J (4-3)	m(r)-AEJ=JA (3-3)
**42**	924.40441	gm(r)-AEJA	N/A
**43**	942.41498	A=gm(r)-AEJ (4-3)	gm(r)-AEJA
**44**	951.41531	N/A	m(r)-AEJ=EJ (3-3)
**45**	1022.4524	N/A	m(r)-AEJ=AEJ (3-3)
**46**	1025.4521	N/A	gm(r)-AEJ=J (3-3)
**47**	1040.463	m(r)-AEJA=EJ (4-3)	m(r)-AEJ=EJA (3-3)
**48**	1111.5001	m(r)-AEJA=AEJ (4-3)	m(r)-AEJ=AEJA (3-3)
**49**	1114.4998	gm(r)-AEJA=J (4-3)	gm(r)-AEJ=JA (3-3)
**50**	1154.4947	N/A	gm(r)-AEJ=EJ (3-3)
**51**	1225.5318	N/A	gm(r)-AEJ=AEJ (3-3)
**52**	1243.5424	gm(r)-AEJA=EJ (4-3)	gm(r)-AEJ=EJA (3-3)
**53**	1299.5686	N/A	m(r)-AEJ=m(r)-AEJ (3-3)
**54**	1314.5795	gm(r)-AEJA=AEJ (4-3)	gm(r)-AEJ=AEJA (3-3)
**55**	1388.6163	m(r)-AEJA=m(r)-AEJ (4-3)	m(r)-AEJ=m(r)-AEJA (3-3)
**56**	1502.648	N/A	m(r)-AEJ=gm(r)-AEJ (3-3)
**57**	1591.6956	m(r)-AEJA=gm(r)-AEJ (4-3)	m(r)-AEJ=gm(r)-AEJA (3-3)
**58**	1705.7273	N/A	gm(r)-AEJ=gm(r)-AEJ (3-3)
**59**	1794.775	gm(r)-AEJA=gm(r)-AEJ (4-3)	gm(r)-AEJ=gm(r)-AEJA (3-3)

***** N/A, Not applicable (does not exist)

** C-terminal residue (+H_2O)

Table S11. List of validated dimers

	Structure	Crosslink	RT (min)	TheoMw	Intensity	Abundance	Acceptor	Donor
**1**	gm-AEJA=gm-AEJA|2	3-4	17.52	1864.8048	9.28E+08	12.41%	gm-AEJA	gm-AEJAX
**2**	gm-AEJA=gm-AEJ|2	3-3	15.90	1793.7676	8.75E+08	11.71%		gm-AEJX
**3**	gm-AEJG=gm-AEJ|2	3-3	27.88	1779.7520	6.16E+08	8.23%	gm-AEJG	gm-AEJX
**4**	gm-AEJ=gm-AEJ|2	3-3	14.01	1722.7305	4.88E+08	6.52%	gm-AEJ	gm-AEJX
**5**	gm-AEJG=gm-AEJA|2	3-4	14.82	1850.7891	2.96E+08	3.96%	gm-AEJG	gm-AEJAX
**6**	gm-AEJF=gm-AEJ|2	3-3	23.53	1869.7989	9.12E+07	1.22%	gm-AEJF	gm-AEJX
**7**	gm-AEJN=gm-AEJ|2	3-3	12.96	1836.7734	7.00E+07	0.94%	gm-AEJN	gm-AEJX
**8**	gm-AEJAG=gm-AEJA|2<br>gm-AEJQ=gm-AEJA|2	3-4	15.05	1921.8263	5.95E+07	0.80%	gm-AEJAG<br>gm-AEJQ	gm-AEJAX<br>gm-AEJAX
**9**	gm-AEJS=gm-AEJ|2	3-3	13.21	1809.7625	4.50E+07	0.60%	gm-AEJS	gm-AEJX
**10**	gm-AEJAI=gm-AEJA|2	3-4	24.15	1977.8888	3.42E+07	0.46%	gm-AEJAI	gm-AEJAX
**11**	gm-AEJAI=gm-AEJ|2	3-3	22.72	1906.8518	3.31E+07	0.44%	gm-AEJAI	gm-AEJX
**12**	gm-AEJAF=gm-AEJ|2	3-3	24.36	1940.8360	3.19E+07	0.43%	gm-AEJAF	gm-AEJX
**13**	gm-AEJN=gm-AEJA|2	3-4	10.54	1907.8105	2.19E+07	0.29%	gm-AEJN	gm-AEJAX
**14**	gm-AEJS=gm-AEJA|2	3-4	14.08	1880.7996	1.86E+07	0.25%	gm-AEJS	gm-AEJAX
**15**	gm-AEJY=gm-AEJ|2	3-3	19.61	1885.7938	1.79E+07	0.24%	gm-AEJY	gm-AEJX
**16**	gm-AEJAD=gm-AEJA|2	3-4	16.08	1979.8317	1.60E+07	0.21%	gm-AEJAD	gm-AEJAX
**17**	gm-AEJK=gm-AEJ|2	3-3	13.07	1850.8254	1.54E+07	0.21%	gm-AEJK	gm-AEJX
**18**	gm-AEJM=gm-AEJ|2	3-3	18.83	1853.7710	1.41E+07	0.19%	gm-AEJM	gm-AEJX
**19**	gm-AEJAA=gm-AEJA|2	3-4	16.33	1935.8419	1.34E+07	0.18%	gm-AEJAA	gm-AEJAX
**20**	gm-AEJI=gm-AEJ|2	3-3	22.38	1835.8145	1.31E+07	0.18%	gm-AEJI	gm-AEJX
**21**	gm-AEJH=gm-AEJ|2	3-3	12.63	1859.7894	1.27E+07	0.17%	gm-AEJH	gm-AEJX
**22**	gm-AEJH=gm-AEJA|2	3-4	13.27	1930.8265	8.00E+06	0.11%	gm-AEJH	gm-AEJAX
**23**	gm-AEJT=gm-AEJ|2	3-3	13.72	1823.7782	7.76E+06	0.10%	gm-AEJT	gm-AEJX
**24**	gm-AEJY=gm-AEJA|2	3-4	16.77	1956.8309	6.87E+06	0.09%	gm-AEJY	gm-AEJAX
**25**	gm-AEJAW=gm-AEJ|2	3-3	26.49	1979.8469	6.11E+06	0.08%	gm-AEJAW	gm-AEJX
**26**	gm-AEJAM=gm-AEJA|2	3-4	20.43	1995.8452	5.82E+06	0.08%	gm-AEJAM	gm-AEJAX
**27**	gm-AEJAM=gm-AEJ|2	3-3	19.41	1924.8081	5.68E+06	0.08%	gm-AEJAM	gm-AEJX
**28**	gm-AEJK=gm-AEJA|2	3-4	10.28	1921.8625	5.35E+06	0.07%	gm-AEJK	gm-AEJAX
**29**	gm-AEJD=gm-AEJA|2	3-4	14.49	1908.7946	5.32E+06	0.07%	gm-AEJD	gm-AEJAX
**30**	gm-AEJAW=gm-AEJA|2	3-4	27.00	2050.8840	5.24E+06	0.07%	gm-AEJAW	gm-AEJAX
**31**	gm-AEJV=gm-AEJ|2	3-3	19.14	1821.7989	4.19E+06	0.06%	gm-AEJV	gm-AEJX
**32**	gm-AEJF=gm-AEJA|2	3-4	25.26	1940.8360	3.89E+06	0.05%	gm-AEJF	gm-AEJAX
**33**	gm-AEJD=gm-AEJ|2	3-3	13.69	1837.7574	3.85E+06	0.05%	gm-AEJD	gm-AEJX
**34**	gm-AEJT=gm-AEJA|2	3-4	14.67	1894.8153	3.71E+06	0.05%	gm-AEJT	gm-AEJAX
**35**	gm-AEJAR=gm-AEJ|2	3-3	11.09	1949.8687	3.46E+06	0.05%	gm-AEJAR	gm-AEJX
**36**	gm-AEJAF=gm-AEJA|2	3-4	25.89	2011.8731	2.95E+06	0.04%	gm-AEJAF	gm-AEJAX
**37**	gm-AEJW=gm-AEJ|2	3-3	21.70	1908.8098	2.86E+06	0.04%	gm-AEJW	gm-AEJX
**38**	gm-AEJAH=gm-AEJA|2	3-4	11.02	2001.8636	2.26E+06	0.03%	gm-AEJAH	gm-AEJAX
**39**	gm-AEJV=gm-AEJA|2	3-4	19.89	1892.8360	2.08E+06	0.03%	gm-AEJV	gm-AEJAX
**40**	gm-AEJI=gm-AEJA|2	3-4	23.45	1906.8518	2.02E+06	0.03%	gm-AEJI	gm-AEJAX
**41**	gm-AEJM=gm-AEJA|2	3-4	19.76	1924.8081	1.53E+06	0.02%	gm-AEJM	gm-AEJAX
**42**	gm-AEJAE=gm-AEJA|2	3-4	15.81	1993.8473	1.14E+06	0.02%	gm-AEJAE	gm-AEJAX
**43**	gm-AEJ=gm-AEJA|2	3-4	14.30	1793.7676	1.09E+06	0.01%	gm-AEJ	gm-AEJAX
**44**	gm-AEJAK=gm-AEJA|2	3-4	13.82	1992.8997	1.01E+06	0.01%	gm-AEJAK	gm-AEJAX
**45**	gm-AEJAQ=gm-AEJA|2	3-4	16.36	1992.8633	9.37E+05	0.01%	gm-AEJAQ	gm-AEJAX
**46**	gm-AEJAA=gm-AEJ|2	3-3	17.60	1864.8048	6.93E+05	0.01%	gm-AEJAA	gm-AEJX

Table S12. List of unmodified muropeptides in TY1, TY2 and TY3 samples

Nb	Structure	Theo mass (Da)	Average Intensity	Average %	% mono- di-, trimers	Cumulated abundance	Average RT (min)	Intensity TY1	Intensity TY2	Intensity TY3	
1	gm-AEJA|1	941.4078	1.07E+09	14.361%	47.90%	47.90%	9.50 ± 0.03	9.1E+08	1.1E+09	1.2E+09	
2	gm-AEJG|1	927.3922	2.43E+08	3.249%	10.84%	58.74%	6.85 ± 0.04	2.5E+08	2.3E+08	2.5E+08	
3	gm-AEJ|1	870.3707	1.64E+08	2.199%	7.33%	66.07%	5.48 ± 0.03	1.7E+08	1.3E+08	1.9E+08	
4	gm-AEJAI|1	1054.4919	1.54E+08	2.054%	6.85%	72.92%	21.40 ± 0.00	1.6E+08	1.5E+08	1.5E+08	
5	gm-AEJF|1	1017.4391	1.48E+08	1.976%	6.59%	79.51%	21.62 ± 0.00	1.4E+08	1.5E+08	1.5E+08	
6	gm-AEJAA|1	1012.4450	6.55E+07	0.875%	2.92%	82.43%	10.86 ± 0.02	5.0E+07	7.3E+07	7.4E+07	
7	gm-AEJAF|1	1088.4762	5.00E+07	0.669%	2.23%	84.67%	15.38 ± 0.00	6.0E+07	5.2E+07	3.9E+07	
8	gm-AEJ[AG/Q]|1	998.4292	4.61E+07	0.616%	2.06%	86.72%	8.80 ± 0.05	4.3E+07	4.3E+07	5.3E+07	
9	gm-AEJK|1	998.4656	4.60E+07	0.615%	2.05%	88.77%	7.71 ± 0.06	6.2E+07	4.0E+07	3.6E+07	
10	gm-AEJY|1	1033.4340	3.38E+07	0.452%	1.51%	90.28%	16.84 ± 0.01	3.3E+07	3.6E+07	3.2E+07	
11	gm-AEJAM|1	1072.4483	2.87E+07	0.384%	1.28%	91.56%	16.56 ± 0.00	2.8E+07	3.1E+07	2.7E+07	
12	gm-AEJN|1	984.4136	2.70E+07	0.361%	1.20%	92.77%	5.03 ± 0.02	2.5E+07	2.4E+07	3.1E+07	
13	gm-AEJAD|1	1056.4348	2.52E+07	0.337%	1.13%	93.89%	9.61 ± 0.04	3.7E+07	2.0E+07	1.9E+07	
14	gm-AEJM|1	1001.4112	2.03E+07	0.272%	0.91%	94.80%	15.59 ± 0.01	2.1E+07	2.3E+07	1.7E+07	
15	gm-AEJI|1	983.4547	1.81E+07	0.242%	0.81%	95.61%	20.26 ± 0.01	2.0E+07	1.8E+07	1.6E+07	
16	gm-AE|1	698.2859	1.73E+07	0.232%	0.77%	96.38%	8.74 ± 0.05	2.3E+07	1.1E+07	1.8E+07	
17	gm-AEJS|1	957.4027	1.58E+07	0.211%	0.70%	97.09%	5.79 ± 0.13	1.7E+07	1.0E+07	2.0E+07	
18	gm-AEJAW|1	1127.4871	1.47E+07	0.197%	0.66%	97.74%	25.19 ± 0.00	1.4E+07	1.6E+07	1.4E+07	
19	gm-AEJH|1	1007.4296	9.64E+06	0.129%	0.43%	98.17%	5.93 ± 0.01	1.1E+07	8.7E+06	8.9E+06	
20	gm-AEJW|1	1056.4500	6.41E+06	0.086%	0.29%	98.46%	23.89 ± 0.00	6.5E+06	7.0E+06	5.8E+06	
21	gm-AEJV|1	969.4391	6.29E+06	0.084%	0.28%	98.74%	14.56 ± 0.01	6.1E+06	6.5E+06	6.3E+06	
22	gm-AEJAQ|1	1069.4664	5.40E+06	0.072%	0.24%	98.98%	11.03 ± 0.03	5.5E+06	5.0E+06	5.7E+06	
23	gm-AEJAH|1	1078.4667	5.02E+06	0.067%	0.22%	99.20%	7.25 ± 0.05	5.6E+06	5.0E+06	4.5E+06	
24	gm-AEJT|1	971.4184	4.51E+06	0.060%	0.20%	99.40%	7.63 ± 0.05	4.5E+06	4.7E+06	4.4E+06	
25	gm-AEJAE|1	1070.4504	4.51E+06	0.060%	0.20%	99.61%	10.67 ± 0.04	3.8E+06	3.7E+06	6.0E+06	
26	gm-AEJAR|1	1097.5089	4.11E+06	0.055%	0.18%	99.79%	9.07 ± 0.06	3.7E+06	4.1E+06	4.5E+06	
27	gm-AEJAK|1	1069.5028	2.53E+06	0.034%	0.11%	99.90%	7.30 ± 0.06	3.6E+06	2.1E+06	1.9E+06	
28	gm-AEJD|1	985.3976	2.21E+06	0.030%	0.10%	100.00%	7.03 ± 0.19	2.8E+06	7.6E+05	3.1E+06	**29.98%**
29	gm-AEJA=gm-AEJA|2	1864.8048	9.28E+08	12.411%	24.39%	24.39%	15.70 ± 0.01	9.5E+08	9.2E+08	9.2E+08	
30	gm-AEJA=gm-AEJ|2	1793.7676	8.75E+08	11.707%	23.01%	47.39%	14.91 ± 0.02	8.6E+08	8.9E+08	8.7E+08	
31	gm-AEJG=gm-AEJ|2	1779.7520	6.16E+08	8.234%	16.18%	63.57%	13.63 ± 0.01	6.3E+08	6.3E+08	5.9E+08	
32	gm-AEJ=gm-AEJ|2	1722.7305	4.88E+08	6.524%	12.82%	76.39%	13.93 ± 0.01	5.1E+08	4.9E+08	4.6E+08	

**33**	gm-AEJG=gm-AEJA|2	1850.7891	2.96E+08	3.964%	7.79%	84.18%	14.37 ± 0.02	2.9E+08	3.0E+08	3.0E+08
**34**	gm-AEJF=gm-AEJ|2	1869.7989	9.12E+07	1.220%	2.40%	86.58%	23.89 ± 0.07	1.1E+08	8.5E+07	7.5E+07
**35**	gm-AEJN=gm-AEJ|2	1836.7734	7.00E+07	0.936%	1.84%	88.42%	12.95 ± 0.01	7.2E+07	7.0E+07	6.7E+07
**36**	gm-AEJ[AG/Q]=gm-AEJA|2	1921.8262	5.95E+07	0.796%	1.56%	89.99%	15.02 ± 0.02	5.8E+07	6.2E+07	5.9E+07
**37**	gm-AEJS=gm-AEJ|2	1809.7625	4.50E+07	0.602%	1.18%	91.17%	13.31 ± 0.01	4.6E+07	4.9E+07	4.0E+07
**38**	gm-AEJAI=gm-AEJA|2	1977.8888	3.42E+07	0.457%	0.90%	92.07%	24.15 ± 0.00	3.2E+07	3.3E+07	3.7E+07
**39**	gm-AEJAI=gm-AEJ|2	1906.8517	3.31E+07	0.442%	0.87%	92.94%	22.72 ± 0.01	3.4E+07	3.3E+07	3.3E+07
**40**	gm-AEJAF=gm-AEJ|2	1940.8360	3.19E+07	0.426%	0.84%	93.77%	24.69 ± 0.01	3.2E+07	3.2E+07	3.1E+07
**41**	gm-AEJN=gm-AEJA|2	1907.8105	2.19E+07	0.292%	0.57%	94.35%	13.64 ± 0.01	2.4E+07	2.2E+07	1.9E+07
**42**	gm-AEJS=gm-AEJA|2	1880.7996	1.86E+07	0.249%	0.49%	94.84%	14.06 ± 0.02	1.8E+07	2.0E+07	1.8E+07
**43**	gm-AEJY=gm-AEJ|2	1885.7938	1.79E+07	0.240%	0.47%	95.31%	19.59 ± 0.01	2.0E+07	1.7E+07	1.7E+07
**44**	gm-AEJAD=gm-AEJA|2	1979.8317	1.60E+07	0.214%	0.42%	95.73%	16.77 ± 0.01	2.3E+07	1.6E+07	8.7E+06
**45**	gm-AEJK=gm-AEJ|2	1850.8254	1.54E+07	0.206%	0.40%	96.13%	12.57 ± 0.01	2.0E+07	1.5E+07	1.2E+07
**46**	gm-AEJM=gm-AEJ|2	1853.7710	1.41E+07	0.188%	0.37%	96.50%	19.04 ± 0.01	1.6E+07	1.5E+07	1.2E+07
**47**	gm-AEJAA=gm-AEJA|2	1935.8419	1.34E+07	0.179%	0.35%	96.86%	16.27 ± 0.05	1.2E+07	1.4E+07	1.4E+07
**48**	gm-AEJI=gm-AEJ|2	1835.8145	1.31E+07	0.175%	0.34%	97.20%	22.78 ± 0.01	1.3E+07	1.4E+07	1.2E+07
**49**	gm-AEJH=gm-AEJ|2	1859.7894	1.27E+07	0.170%	0.33%	97.53%	12.62 ± 0.01	1.3E+07	1.3E+07	1.2E+07
**50**	gm-AEJH=gm-AEJA|2	1930.8265	8.00E+06	0.107%	0.21%	97.74%	13.27 ± 0.01	8.5E+06	8.0E+06	7.5E+06
**51**	gm-AEJT=gm-AEJ|2	1823.7782	7.76E+06	0.104%	0.20%	97.95%	13.93 ± 0.01	8.4E+06	7.9E+06	7.0E+06
**52**	gm-AEJY=gm-AEJA|2	1956.8309	6.87E+06	0.092%	0.18%	98.13%	20.40 ± 0.01	7.2E+06	6.9E+06	6.5E+06
**53**	gm-AEJAW=gm-AEJ|2	1979.8469	6.11E+06	0.082%	0.16%	98.29%	26.49 ± 0.01	6.4E+06	6.3E+06	5.6E+06
**54**	gm-AEJAM=gm-AEJA|2	1995.8452	5.82E+06	0.078%	0.15%	98.44%	20.43 ± 0.01	5.8E+06	6.2E+06	5.5E+06
**55**	gm-AEJAM=gm-AEJ|2	1924.8081	5.68E+06	0.076%	0.15%	98.59%	19.39 ± 0.02	6.6E+06	5.8E+06	4.6E+06
**56**	gm-AEJK=gm-AEJA|2	1921.8626	5.35E+06	0.072%	0.14%	98.73%	13.22 ± 0.01	5.7E+06	5.6E+06	4.8E+06
**57**	gm-AEJD=gm-AEJA|2	1908.7946	5.32E+06	0.071%	0.14%	98.87%	14.49 ± 0.02	7.4E+06	4.8E+06	3.8E+06
**58**	gm-AEJAW=gm-AEJA|2	2050.8840	5.24E+06	0.070%	0.14%	99.01%	27.00 ± 0.01	6.2E+06	5.6E+06	3.9E+06
**59**	gm-AEJV=gm-AEJ|2	1821.7989	4.19E+06	0.056%	0.11%	99.12%	19.13 ± 0.01	4.7E+06	4.3E+06	3.6E+06
**60**	gm-AEJF=gm-AEJA|2	1940.8360	3.89E+06	0.052%	0.10%	99.22%	25.27 ± 0.00	4.0E+06	3.9E+06	3.8E+06
**61**	gm-AEJD=gm-AEJ|2	1837.7574	3.85E+06	0.051%	0.10%	99.32%	13.68 ± 0.01	3.9E+06	4.1E+06	3.5E+06
**62**	gm-AEJT=gm-AEJA|2	1894.8153	3.71E+06	0.050%	0.10%	99.42%	14.67 ± 0.02	3.6E+06	3.7E+06	3.8E+06
**63**	gm-AEJAR=gm-AEJ|2	1949.8687	3.46E+06	0.046%	0.09%	99.51%	13.95 ± 0.06	3.9E+06	3.5E+06	3.1E+06
**64**	gm-AEJAF=gm-AEJA|2	2011.8731	2.95E+06	0.039%	0.08%	99.59%	25.90 ± 0.01	3.1E+06	2.7E+06	3.0E+06
**65**	gm-AEJW=gm-AEJ|2	1908.8098	2.86E+06	0.038%	0.08%	99.66%	25.36 ± 0.01	3.2E+06	3.0E+06	2.4E+06
**66**	gm-AEJAH=gm-AEJA|2	2001.8636	2.26E+06	0.030%	0.06%	99.72%	13.94 ± 0.01	2.3E+06	2.3E+06	2.2E+06
**67**	gm-AEJV=gm-AEJA|2	1892.8360	2.08E+06	0.028%	0.05%	99.78%	19.89 ± 0.01	2.0E+06	2.9E+06	1.4E+06
**68**	gm-AEJI=gm-AEJA|2	1906.8517	2.02E+06	0.027%	0.05%	99.83%	23.45 ± 0.00	2.1E+06	2.1E+06	1.9E+06
**69**	gm-AEJM=gm-AEJA|2	1924.8081	1.53E+06	0.020%	0.04%	99.87%	19.74 ± 0.01	1.7E+06	1.8E+06	1.1E+06

**70**	gm-AEJAE=gm-AEJA|2	1993.8473	1.14E+06	0.015%	0.03%	99.90%	15.81 ± 0.01	1.0E+06	1.2E+06	1.2E+06	
**71**	gm-AEJ=gm-AEJA|2	1793.7676	1.09E+06	0.015%	0.03%	99.93%	14.29 ± 0.01	9.8E+05	1.1E+06	1.1E+06	
**72**	gm-AEJAK=gm-AEJA|2	1992.8997	1.01E+06	0.014%	0.03%	99.96%	13.82 ± 0.01	1.1E+06	1.1E+06	9.1E+05	
**73**	gm-AEJAQ=gm-AEJA|2	1992.8633	9.37E+05	0.013%	0.02%	99.98%	16.36 ± 0.01	8.7E+05	9.6E+05	9.8E+05	
**74**	gm-AEJAA=gm-AEJ|2	1864.8048	6.93E+05	0.009%	0.02%	100.00%	17.60 ± 0.01	7.2E+05	4.9E+05	9.0E+05	**50.89%**
**75**	gm-AEJA=gm-AEJA=gm-AEJA|3	2788.2017	2.61E+08	3.493%	18.26%	18.26%	18.62 ± 0.01	2.7E+08	2.6E+08	2.6E+08	
**76**	gm-AEJA=gm-AEJ=gm-AEJA|3	2717.1646	2.59E+08	3.461%	18.09%	36.35%	18.10 ± 0.01	2.7E+08	2.6E+08	2.5E+08	
**77**	gm-AEJA=gm-AEJ=gm-AEJ|3	2646.1274	2.27E+08	3.042%	15.90%	52.25%	17.61 ± 0.01	2.2E+08	2.4E+08	2.2E+08	
**78**	gm-AEJG=gm-AEJ=gm-AEJ|3	2632.1118	1.73E+08	2.311%	12.08%	64.32%	16.45 ± 0.01	1.7E+08	1.7E+08	1.8E+08	
**79**	gm-AEJ=gm-AEJ=gm-AEJ|3	2575.0903	1.34E+08	1.787%	9.34%	73.66%	16.74 ± 0.01	1.4E+08	1.4E+08	1.3E+08	
**80**	gm-AEJG=gm-AEJ=gm-AEJA|3	2703.1489	1.07E+08	1.429%	7.47%	81.13%	16.97 ± 0.01	1.0E+08	1.1E+08	1.1E+08	
**81**	gm-AEJG=gm-AEJA=gm-AEJA|3	2774.1860	7.71E+07	1.031%	5.39%	86.52%	17.48 ± 0.01	7.7E+07	7.5E+07	7.9E+07	
**82**	gm-AEJF=gm-AEJ=gm-AEJ|3	2722.1587	2.89E+07	0.387%	2.02%	88.54%	25.04 ± 0.00	3.2E+07	2.9E+07	2.6E+07	
**83**	gm-AEJ[AG/Q]=gm-AEJA=gm-AEJA|3	2845.2231	1.92E+07	0.257%	1.34%	89.88%	18.00 ± 0.01	1.8E+07	2.0E+07	2.0E+07	
**84**	gm-AEJN=gm-AEJ=gm-AEJ|3	2689.1332	1.85E+07	0.247%	1.29%	91.17%	15.84 ± 0.01	1.8E+07	1.9E+07	1.8E+07	
**85**	gm-AEJS=gm-AEJ=gm-AEJ|3	2662.1223	1.29E+07	0.173%	0.90%	92.08%	16.15 ± 0.01	1.3E+07	1.4E+07	1.2E+07	
**86**	gm-AEJF=gm-AEJ=gm-AEJA|3	2793.1958	1.10E+07	0.148%	0.77%	92.85%	25.55 ± 0.01	1.1E+07	1.1E+07	1.1E+07	
**87**	gm-AEJN=gm-AEJ=gm-AEJA|3	2760.1703	1.03E+07	0.138%	0.72%	93.57%	16.34 ± 0.01	1.0E+07	1.0E+07	1.0E+07	
**88**	gm-AEJN=gm-AEJA=gm-AEJA|3	2831.2074	7.61E+06	0.102%	0.53%	94.10%	16.86 ± 0.01	7.4E+06	8.1E+06	7.3E+06	
**89**	gm-AEJF=gm-AEJA=gm-AEJA|3	2864.2329	7.16E+06	0.096%	0.50%	94.60%	26.00 ± 0.01	7.1E+06	7.6E+06	6.7E+06	
**90**	gm-AEJS=gm-AEJ=gm-AEJA|3	2733.1594	6.25E+06	0.084%	0.44%	95.04%	16.66 ± 0.00	6.5E+06	6.5E+06	5.7E+06	
**91**	gm-AEJAI=gm-AEJ=gm-AEJ|3	2759.2115	5.65E+06	0.076%	0.40%	95.43%	24.60 ± 0.01	5.1E+06	5.6E+06	6.2E+06	
**92**	gm-AEJH=gm-AEJ=gm-AEJ|3	2712.1492	5.07E+06	0.068%	0.35%	95.79%	17.02 ± 1.06	5.3E+06	4.8E+06	5.0E+06	
**93**	gm-AEJAI=gm-AEJA=gm-AEJA|3	2901.2857	5.06E+06	0.068%	0.35%	96.14%	25.57 ± 0.01	4.8E+06	5.0E+06	5.4E+06	
**94**	gm-AEJS=gm-AEJA=gm-AEJA|3	2804.1965	4.96E+06	0.066%	0.35%	96.49%	17.19 ± 0.01	5.1E+06	5.1E+06	4.7E+06	
**95**	gm-AEJAI=gm-AEJ=gm-AEJA|3	2830.2486	4.63E+06	0.062%	0.32%	96.81%	25.05 ± 0.01	4.9E+06	4.5E+06	4.5E+06	
**96**	gm-AEJY=gm-AEJ=gm-AEJ|3	2738.1536	4.36E+06	0.058%	0.30%	97.12%	21.34 ± 0.01	4.4E+06	4.8E+06	3.9E+06	
**97**	gm-AEJM=gm-AEJ=gm-AEJ|3	2706.1308	4.04E+06	0.054%	0.28%	97.40%	21.39 ± 0.70	4.2E+06	4.3E+06	3.6E+06	
**98**	gm-AEJK=gm-AEJ=gm-AEJ|3	2703.1852	3.43E+06	0.046%	0.24%	97.64%	15.44 ± 0.02	4.4E+06	3.3E+06	2.6E+06	
**99**	gm-AEJAA=gm-AEJA=gm-AEJA|3	2859.2388	3.39E+06	0.045%	0.24%	97.88%	18.99 ± 0.02	3.3E+06	3.2E+06	3.6E+06	
**##**	gm-AEJI=gm-AEJ=gm-AEJ|3	2688.1743	3.21E+06	0.043%	0.22%	98.10%	23.98 ± 0.01	3.5E+06	3.4E+06	2.8E+06	
**##**	gm-AEJAD=gm-AEJ=gm-AEJA|3	2832.1915	2.83E+06	0.038%	0.20%	98.30%	18.86 ± 0.01	3.9E+06	3.1E+06	1.5E+06	
**##**	gm-AEJM=gm-AEJ=gm-AEJA|3	2777.1679	2.13E+06	0.028%	0.15%	98.45%	21.52 ± 0.00	2.8E+06	2.4E+06	1.3E+06	
**##**	gm-AEJT=gm-AEJ=gm-AEJ|3	2676.1380	2.12E+06	0.028%	0.15%	98.60%	16.72 ± 0.01	2.2E+06	2.0E+06	2.2E+06	
**##**	gm-AEJH=gm-AEJ=gm-AEJA|3	2783.1863	1.89E+06	0.025%	0.13%	98.73%	16.03 ± 0.01	2.0E+06	1.8E+06	1.9E+06	
**##**	gm-AEJAM=gm-AEJ=gm-AEJA|3	2848.2050	1.77E+06	0.024%	0.12%	98.85%	22.00 ± 0.00	2.0E+06	1.9E+06	1.4E+06	
**##**	gm-AEJAW=gm-AEJ=gm-AEJ|3	2832.2067	1.74E+06	0.023%	0.12%	98.97%	27.07 ± 0.01	1.6E+06	1.7E+06	1.9E+06	

##	gm-AEJY=gm-AEJ=gm-AEJA|3	2809.1907	1.70E+06	0.023%	0.12%	99.09%	21.83 ±	0.00	1.5E+06	1.8E+06	1.7E+06	
##	gm-AEJY=gm-AEJA=gm-AEJA|3	2880.2278	1.68E+06	0.023%	0.12%	99.21%	22.54 ±	0.00	1.5E+06	1.8E+06	1.8E+06	
##	gm-AEJAW=gm-AEJA=gm-AEJA|3	2974.2809	1.48E+06	0.020%	0.10%	99.31%	24.47 ±	4.82	3.3E+06	6.0E+05	6.0E+05	
##	gm-AEJH=gm-AEJA=gm-AEJA|3	2854.2234	1.38E+06	0.018%	0.10%	99.41%	16.49 ±	0.03	2.0E+06	1.1E+06	9.9E+05	
##	gm-AEJV=gm-AEJ=gm-AEJ|3	2674.1587	1.19E+06	0.016%	0.08%	99.49%	21.35 ±	0.01	1.3E+06	1.2E+06	1.1E+06	
##	gm-AEJAM=gm-AEJA=gm-AEJA|3	2919.2421	1.02E+06	0.014%	0.07%	99.56%	22.46 ±	0.01	1.2E+06	1.1E+06	7.7E+05	
##	gm-AEJK=gm-AEJ=gm-AEJA|3	2774.2224	9.90E+05	0.013%	0.07%	99.63%	15.94 ±	0.01	1.1E+06	8.8E+05	1.0E+06	
##	gm-AEJD=gm-AEJ=gm-AEJ|3	2690.1172	9.18E+05	0.012%	0.06%	99.70%	17.22 ±	0.01	6.5E+05	6.7E+05	1.4E+06	
##	gm-AEJW=gm-AEJ=gm-AEJ|3	2761.1696	8.96E+05	0.012%	0.06%	99.76%	26.15 ±	0.01	1.1E+06	9.1E+05	7.1E+05	
##	gm-AEJW=gm-AEJA=gm-AEJA|3	2903.2438	8.42E+05	0.011%	0.06%	99.82%	27.44 ±	0.02	8.3E+05	6.0E+05	1.1E+06	
##	gm-AEJT=gm-AEJ=gm-AEJA|3	2747.1751	7.13E+05	0.010%	0.05%	99.87%	17.21 ±	0.01	7.3E+05	7.4E+05	6.6E+05	
##	gm-AEJAD=gm-AEJA=gm-AEJA|3	2903.2286	6.36E+05	0.009%	0.04%	99.91%	19.31 ±	0.02	9.1E+05	6.6E+05	3.4E+05	
##	gm-AEJV=gm-AEJ=gm-AEJA|3	2745.1958	4.90E+05	0.007%	0.03%	99.95%	21.71 ±	0.00	4.3E+05	5.5E+05	4.9E+05	
##	gm-AEJV=gm-AEJA=gm-AEJA|3	2816.2329	4.31E+05	0.006%	0.03%	99.98%	22.10 ±	0.01	4.4E+05	4.2E+05	4.4E+05	
##	gm-AEJAD=gm-AEJ=gm-AEJ|3	2761.1544	3.08E+05	0.004%	0.02%	100.00%	17.83 ±	0.01	4.3E+05	2.5E+05	2.4E+05	**19.13%**
			7.48E+09	100.000%								

Table S13. Database 5 (DB_5)

Structure	Monoisotopicmass
gm-AE|1	698.28580
gm-AEJ|1	870.37059
gm-AEJA|1	941.40770
gm-AEJAA|1	1012.44482
gm-AEJAD|1	1056.43465
gm-AEJAE|1	1070.45030
gm-AEJAF|1	1088.47612
gm-AEJAG|1	998.42917
gm-AEJAH|1	1078.46661
gm-AEJAI|1	1054.49177
gm-AEJAK|1	1069.50267
gm-AEJAM|1	1072.44819
gm-AEJAQ|1	1069.46628
gm-AEJAR|1	1097.50881
gm-AEJAW|1	1127.48702
gm-AEJD|1	985.39753
gm-AEJF|1	1017.43900
gm-AEJG|1	927.39205
gm-AEJH|1	1007.42950
gm-AEJI|1	983.45465
gm-AEJK|1	998.46555
gm-AEJM|1	1001.41107
gm-AEJN|1	984.41352
gm-AEJQ|1	998.42917
gm-AEJS|1	957.40262
gm-AEJT|1	971.41827
gm-AEJV|1	969.43900
gm-AEJW|1	1056.44990
gm-AEJY|1	1033.43392
gm-AEJAV|1	1040.47612
gm-AEJADA|1	1127.47176
gm-AEJADAI|1	1240.55582
gm-AEJADAIV|1	1339.62424
gm-AEJADAIVA|1	1410.66135
gm-AEJADAIVAA|1	1481.69846
gm-AEJADAIVAAE|1	1610.74106
gm-AEJADAIVAAEP|1	1707.79382
gm-AEJADAIVAAEPE|1	1836.83641
gm-AEJADAIVAAEPEP|1	1933.88918
gm-AEJADAIVAAEPEPV|1	2032.95759
gm-AEJADAIVAAEPEPVE|1	2162.00019
gm-AEJADAIVAAEPEPVEY|1	2325.06351
gm-AEJADAIVAAEPEPVEYV|1	2424.13193
gm-AEJADAIVAAEPEPVEYVR|1	2580.23304
gm-AEJADAIVAAEPEPVEYVRV|1	2679.30145
gm-AEJADAIVAAEPEPVEYVRVC|1	2782.31064
gm-AEJADAIVAAEPEPVEYVRVCD|1	2897.33758
gm-AEJADAV|1	1226.54017
gm-AEJADAVD|1	1341.56712
gm-AEJADAVDQ|1	1469.62569
gm-AEJADAVDQV|1	1568.69411
gm-AEJADAVDQVP|1	1665.74687
gm-AEJADAVDQVPE|1	1794.78946
gm-AEJADAVDQVPEA|1	1865.82658
gm-AEJADAVDQVPEAP|1	1962.87934
gm-AEJADAVDQVPEAPV|1	2061.94776
gm-AEJADAVDQVPEAPVA|1	2132.98487
gm-AEJADAVDQVPEAPVAQ|1	2261.04345
gm-AEJADAVDQVPEAPVAQE|1	2390.08604
gm-AEJADAVDQVPEAPVAQEA|1	2461.12315
gm-AEJADAVDQVPEAPVAQEAP|1	2558.17592
gm-AEJADAVDQVPEAPVAQEAPV|1	2657.24433
gm-AEJADAVDQVPEAPVAQEAPVK|1	2785.33929
gm-AEJADL|1	1169.51871
gm-AEJADLG|1	1226.54017
gm-AEJADLGT|1	1327.58785
gm-AEJADLGTR|1	1483.68896
gm-AEJADLGTRT|1	1584.73664
gm-AEJADLGTRTY|1	1747.79997
gm-AEJADLGTRTYE|1	1876.84256
gm-AEJADLGTRTYEE|1	2005.88516
gm-AEJADLGTRTYEEP|1	2102.93792
gm-AEJADLGTRTYEEPD|1	2217.96486
gm-AEJADLGTRTYEEPDL|1	2331.04893
gm-AEJADLGTRTYEEPDLR|1	2487.15004
gm-AEJADLGTRTYEEPDLRN|1	2601.19296
gm-AEJADLGTRTYEEPDLRNG|1	2658.21443
gm-AEJADLGTRTYEEPDLRNGV|1	2757.28284
gm-AEJADLGTRTYEEPDLRNGVK|1	2885.37781
gm-AEJADLGTRTYEEPDLRNGVKI|1	2998.46187
