# Supplementary material for: A software tool and strategy for peptidoglycomics, the high-resolution analysis of bacterial peptidoglycans via LC-MS/MS
Source: Commun Chem. 2025 Mar 26;8:91. doi: 10.1038/s42004-025-01490-6 (PMC11937551; doi:10.1038/s42004-025-01490-6)
Supplement: Supplementary file 3 — Description of Additional Supplementary Files [file 42004_2025_1490_MOESM3_ESM.pdf]

Description of Additional Supplementary Files

File name: Supplementary Data 1

Description: DB_1 search TY1,2,3 all monomers v1.2.1

File name: Supplementary Data 2

Description: Byos fasta file

File name: Supplementary Data 3

Description: DB_2 search TY1,2,3 Cumulated Monomers abundance v1.2.1

File name: Supplementary Data 4

Description: DB_4 search TY1,2,3 all porins v1.2.1

File name: Supplementary Data 5

Description: DB_2 search TY1,2,3 multimers v1.2.1

File name: Supplementary Data 6

Description: DB_5 search TY123 MM123 Anh_Multimers v1.2.1
